# Genomic evidence of prevalent hybridization throughout the evolutionary history of the fig-wasp pollination mutualism

**DOI:** 10.1038/s41467-021-20957-3

**Published:** 2021-02-02

**Authors:** Gang Wang, Xingtan Zhang, Edward Allen Herre, Doyle McKey, Carlos A. Machado, Wen-Bin Yu, Charles H. Cannon, Michael L. Arnold, Rodrigo A. S. Pereira, Ray Ming, Yi-Fei Liu, Yibin Wang, Dongna Ma, Jin Chen

**Affiliations:** 1 CAS Key Laboratory of Tropical Forest Ecology, Xishuangbanna Tropical Botanical Garden, Chinese Academy of Sciences, Mengla, Yunnan China; 2 Shenzhen Branch, Guangdong Laboratory for Lingnan Modern Agriculture, Genome Analysis Laboratory of the Ministry of Agriculture, Agricultural Genomics Institute at Shenzhen, Chinese Academy of Agricultural Sciences, Shenzhen, China; 3 Center for Plant Ecology, Core Botanical Gardens, Chinese Academy of Sciences, Mengla, Yunnan China; 4 Smithsonian Tropical Research Institute, Balboa, Ancon Republic of Panama; 5 CEFE, University of Montpellier, CNRS, University Paul Valery Montpellier 3, EPHE, IRD, Montpellier, France; 6 Department of Biology, University of Maryland, College Park, MD USA; 7 Center for Integrative Conservation, Xishuangbanna Tropical Botanical Garden, Chinese Academy of Sciences, Mengla, Yunnan China; 8 Center for Tree Science, The Morton Arboretum, Lisle, IL USA; 9 Department of Genetics, University of Georgia, Athens, GA USA; 10 Department of Biology, FFCLRP, University of São Paulo, Ribeirao Preto, São Paulo Brazil; 11 Department of Plant Biology, University of Illinois at Urbana-Champaign, Urbana, IL USA; 12 College of Pharmacy, Hubei University of Chinese Medicine, Wuhan, Hubei China; 13 Center for Genomics and Biotechnology, Fujian Agriculture and Forestry University, Fuzhou, Fujian China; 14 Key Laboratory of the Ministry of Education for Coastal and Wetland Ecosystems, College of the Environment and Ecology, Xiamen University, Xiamen, Fujian China

**Keywords:** Evolutionary ecology, Coevolution, Speciation, Plant evolution

## Abstract

*Ficus* (figs) and their agaonid wasp pollinators present an ecologically important mutualism that also provides a rich comparative system for studying functional co-diversification throughout its coevolutionary history (~75 million years). We obtained entire nuclear, mitochondrial, and chloroplast genomes for 15 species representing all major clades of *Ficus*. Multiple analyses of these genomic data suggest that hybridization events have occurred throughout *Ficus* evolutionary history. Furthermore, cophylogenetic reconciliation analyses detect significant incongruence among all nuclear, chloroplast, and mitochondrial-based phylogenies, none of which correspond with any published phylogenies of the associated pollinator wasps. These findings are most consistent with frequent host-switching by the pollinators, leading to fig hybridization, even between distantly related clades. Here, we suggest that these pollinator host-switches and fig hybridization events are a dominant feature of fig/wasp coevolutionary history, and by generating novel genomic combinations in the figs have likely contributed to the remarkable diversity exhibited by this mutualism.

## Abstract (web summary)

Figs and their wasp pollinators are a classic example of coevolution. By assembling and analysing genomes from across the *Ficus* clade, authors suggest that fig hybridization driven by pollinator host-switching in this obligate pollination system, is more common than previously thought.

## Introduction

Hybridization is increasingly recognized as making important contributions to adaptation and diversification^1–4^. For example, polyploid hybrid diversification is widely accepted in plants, as polyploidy results in rapid reproductive isolation from parental lineages^4,5^. However, the degree to which homoploid hybridization and adaptive introgression contribute to diversification is still actively debated, owing in part to the lack of direct, identifiable reproductive isolation mechanisms^4,6,7^. Animal pollination also is thought to contribute to the diversification of flowering plants, primarily by facilitating reproductive isolation via diversifying pollinator niches and pollinator selection^8–12^. Additionally, animal pollination also facilitates (homoploid) hybrid diversification of flowering plants by generating pollinator-mediated hybrids, followed by the subsequent establishment of pollinator-mediated isolation^6,13–15^. However, empirical studies that focus on the potential for interaction between animal pollination and hybridization to affect and contribute to evolutionary diversification are rare.

Obligate pollination systems, in which plants of a specific taxonomic group are exclusively pollinated by a specific taxonomic group of pollinators, provide an opportunity for detailed examination of these evolutionary interactions^16–20^. These extreme obligate-pollination plants are often more diverse than their non-obligate sister groups^16,18,20,21^. It has been generally accepted that tight pollinator specificity has accelerated diversification, primarily through a mechanism of enhanced reproductive isolation^9,16–18^. However, over the last few decades, an increasing number of empirical studies have demonstrated that, even in extreme obligate pollination systems, pollinator sharing, host switches, and interspecific hybridization occur^14–16,22^, suggesting that strict one-to-one coevolution is not the only evolutionary process occurring in these systems. Thus, a more diffuse model involving pollinator switches and interspecific hybridization is potentially more appropriate for describing speciation and diversification in these “strict-sense” obligate pollination systems^14,15^.

The genus *Ficus* (figs, Moraceae) is one of the most diverse woody plant genera (~800 described species)^21^. With their pollinating fig wasps (Hymenoptera: Chalcidoidea: Agaonidae), they represent perhaps the most extreme and ancient (~75 million years) obligate pollination mutualisms known^20,23^. Figs are distributed in tropical and subtropical regions around the world, with the highest diversity of subgenera and species occurring in South-East Asia^21,24^. Given the observation that morphologically defined taxa (subgenera and sections) of figs are usually pollinated by distinct genera of pollinators, early speculation on the mode of fig and wasp co-evolution emphasized the role of extreme host-specificity of the pollinator wasps coupled with strict-sense coevolution between individual species of figs and their pollinators^17,20,25,26^.

However, broader species sampling combined with increasingly detailed and comprehensive genetic analyses of both host and pollinator species have identified ever larger numbers of exceptions to the previously assumed extreme host specificity. Cases in which multiple wasp species are associated with a single host, as well as cases in which wasp species are shared among two or more hosts, have not only been discovered but also appear to be relatively common^27–31^. Further, in studies of sympatric, closely related figs (within a section) and their respective pollinator species (within a genus)^32,33^, clear cases of phylogenetic incongruence have been documented and are consistent with host-switching that can lead to the apparent hybridization and introgression found among some closely related fig species^32,34–37^. Host switching leading to hybridization is also suggested by studies of distantly related fig and wasp taxa (across sections of figs and genera of pollinators). Specifically, apparent incongruence of both the fig–fig wasp phylogenetic relationships and the nuclear–chloroplast associations of figs across sections of the genus is consistent with the possibility of hybridization at even these deeper taxonomic scales^15,20,32,38^. Collectively, the available data suggest that a more diffuse model of coevolution that incorporates frequent host switching followed by hybridization and introgression might better explain the evolutionary dynamics in this system than strict-sense co-speciation^15,22,33,34^. However, the phylogenetic inferences of much of the existing (suggestive) work are based on data from relatively few, marginally informative genes that only weakly support crucial basal nodes. Further, these studies lack both genetic depth (e.g., entire nuclear and organelle genomes) and a comprehensive sampling design (e.g., taxa that represent what are thought to be the major evolutionary transitions in the genus *Ficus*). Finally, rigorous tests to distinguish true genetic introgression from the alternative process of incomplete lineage sorting (ILS) have generally been lacking.

In this work, we collect complete sequences for nuclear, chloroplast, and mitochondrial genomes from 15 fig species representing all major recognized *Ficus* clades. Previous studies suggest that these clades have been diverging from each other for roughly 75 million years^20^. Integrating complete sequences for two other Moraceae species used as outgroups, we apply multiple analytical approaches to reconstruct phylogenetic relationships among these clades and identify cases of hybridization potentially recorded in the nuclear genomes throughout the evolutionary history of figs. We then compare the inferred evolutionary histories of the nuclear, chloroplast, and mitochondrial genomes across these 15 fig species. Finally, we use published phylogenies of the corresponding pollinator species^20,23^ to assess the evidence for pollinator switches throughout the course of *Ficus* and pollinator wasp evolution. Here, we find direct evidence indicating frequent hybridization and introgression events in fig nuclear genomes. These events are detected even among distantly related, currently non-sympatric lineages of figs, suggesting a process that has been acting throughout the evolutionary history of figs. Specific inferred hybridization events are corroborated by multiple analytical approaches (ABBA-BABA *D*-statistics, PhyloNetworks, and Bayesian Concordance). Furthermore, this inference is also supported by the significant lack of concordance of the topologies of the chloroplast and mitochondrial phylogenies of the 15 *Ficus* species with any supported nuclear phylogenies, or, indeed, with each other. Finally, we show that no currently supported published wasp phylogeny corresponds to any fig phylogeny based on nuclear, chloroplast, or mitochondrial data. Significantly, most hybridization events detected among *Ficus* main clades exhibit associated pollinator host-switch events. We conclude that the evolutionary history of figs and wasps has likely been characterized by frequent host switches of the pollinators and that this process has regularly produced novel genetic combinations within and among host fig genomes.

## Results

### Sequencing and reconstruction of *Ficus* evolution

We used the 13 chromosomes of the *Ficus microcarpa* genome as a reference template^39^ (Supplementary Note 1) to identify the nuclear single nucleotide polymorphisms (SNPs) from 17 Moraceae samples, including 15 fig species representing all main *Ficus* clades (Supplementary Tables 1 and 2). We identified 1,813,332 SNPs from 24,188 genes (including 848,552 SNPs in exons and 964,780 in introns) and 2,660,312 SNPs from intergenic regions, from a total of 196.7 Gbp of raw sequencing data. The average sequencing depth was 26.91× per sample, and 72.76× per SNP locus. The sequenced reads of each *Ficus* sample covered most of the reference genome (*F. microcarpa*), ranging from 88% to 99%. We assembled the circular genomes of both the mitochondria and chloroplasts from the same set of samples for all species. Chloroplast genome size in *Ficus* is consistently around 160 kb (kilobase), while that of mitochondria varies from 430 kb to 711 kb (Supplementary Table 2).

**Fig. 1:**
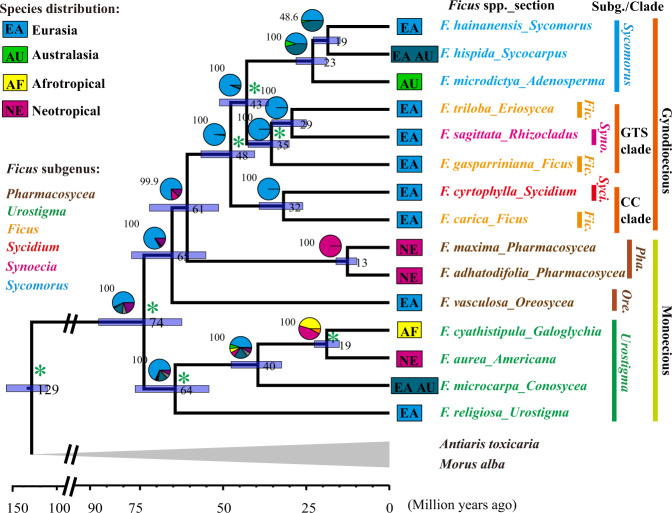
*Ficus* Astral species tree (based exclusively on nuclear data) with inferred divergence times and ancestral geographic ranges of *Ficus*. The Astral *Ficus* species tree was inferred based on non-overlapping 100-kb genomic windows, with almost all nodes being highly supported with bootstrap values >99, excepting one (48.6). The bootstrap values are indicated above the pie chart at each node. Values and bars on the nodes indicate the mean and 95% credibility intervals of node age. The pie charts indicate the relative probability of possible geographic ranges estimated. Current distribution range of related section (tip symbols) and the subgenus information (tip name color) of sampled species are also shown. Green asterisks indicate calibrated nodes used for inferring divergence times. Ranges that are combinations of these four distributions are indicated by colors formed by mixing of the four component distribution colors. Both species distribution and taxonomic classification follow Cruaud et al.^20^. *Ficus* is inferred to have originated in Eurasia and all main clades have more than 10 million years history of coexistence in Eurasia, before dispersal to other areas. This ancestral sympatry could have permitted ancient hybridization among them. Refer to Supplementary Figs. 1–3, Supplementary Table 3, and Appendices 1, 3, and 4 for details.

**Fig. 2:**
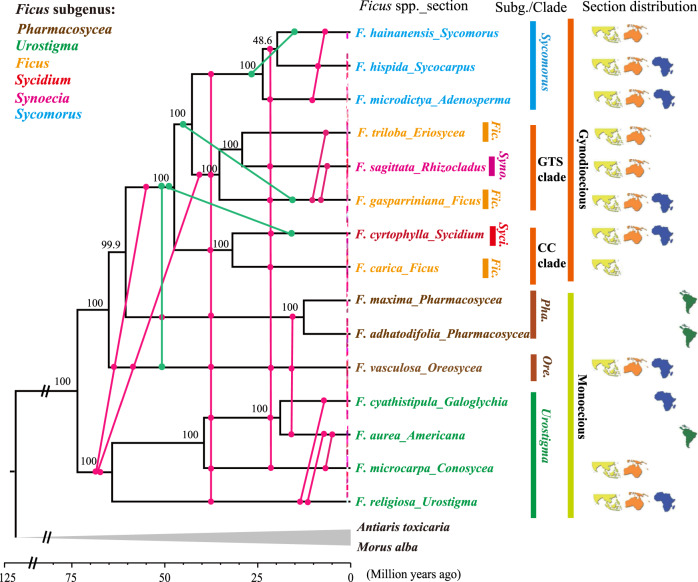
Hybridization events within *Ficus* inferred with ABBA-BABA *D*-statistics and PhyloNetworks exhibited on the Astral tree. The Astral *Ficus* species tree (indicated with horizontal lines) inferred based on the non-overlapping 100-kb-windows nuclear genome datasets, with bootstrap values at nodes. The vertical lines in the tree represent the hybridization events inferred with ABBA-BABA *D*-statistics alone (rose-red lines and dots) or with both the *D*-statistics and PhyloNetworks (green lines and dots). For all pairs of species or clades with a dot where a vertical line crosses the (horizontal) branch indicating the species or clade, hybridization was detected involving taxa connected by the same vertical line. The dashed rose-red line near the tips of branches indicates that signals of hybridization could be detected among almost all species across different subgenera with *D*-statistics. Points where vertical lines contact branches do not reflect the precise estimated time of hybridization, but rather a time interval (between stem and crown age of related taxa) during which hybridization occurred. Current distribution range of related section (map symbols) and the subgenera information (tip name color) of sampled species are also shown. *Ficus* classification and current distribution at the section level are also shown. Refer to Supplementary Data 1 and 2 and Appendices 2 and 6 for details.

Using the nuclear genomic level dataset, coalescent-based Astral species trees^40^ were inferred for *Ficus* based on four types of nuclear genomic sliding windows generated with partition strategies of non-overlapping windows of size 500, 100, and 50 kb, and a SNPs-fixed window of 1000 SNPs (see “Methods” and Supplementary Table 3 for details). The four Astral species trees corresponding to each window size generated similar *Ficus* topologies with high bootstrap values at almost all nodes (Fig. 1, Supplementary Fig. 1A–D, and Appendix 1). All four Astral species trees supported the monophyletic subgenus *Urostigma* (monoecious, pantropical, with both active and passive pollination, Supplementary Note 2) as sister to all other *Ficus* species. After this basal split, the next split supports the two sections of subgenus *Pharmacosycea* (monoecious, with passive pollination for section *Pharmacosycea* and active for section *Oreosycea*) appearing as sister taxa to the monophyletic gynodioecious figs, within which the monophyletic subgenus *Sycomorus* is nested. Similar to results of all previous studies^20,38,39,41^, the two sections of subgenus *Pharmacosycea* did not constitute a monophyletic group. Within the gynodioecious clade, subgenus *Ficus* (part of the gynodioecious clade) also emerged as polyphyletic, while subgenus *Sycomorus*, subgenera *Ficus* + *Synoecia* group (“GTS-clade” in Figs. 1 and 2) and the subgenus *Sycidium* + *F. carica* group (“CC-clade” in Figs. 1 and 2) were recovered as monophyletic with strong support (Fig. 1 and Supplementary Fig. 1).

Consistent with a previous study that included 200 species^20^, our dating and ancestral area reconstructions using the Astral tree based on the complete nuclear genomic data suggest that *Ficus* originated in Eurasia roughly 73.83 (62.39–87.41) million years ago (Mya) (Supplementary Figs. 2A, 3A, Supplementary Table 4, and Appendices 3, 4). The Astral tree-based reconstructions further suggest that all main fig clades coexisted in Eurasia for more than 10 million years. This period of sympatric coexistence appears to have been followed by three major events of expansion out of Eurasia: (1) at ~60.81 (51.35–72.12) Mya, section *Pharmacosycea* colonized the Neotropics, (2) at ~39.52 (32.38–47.60) Mya, the common ancestor of sections *Americana* and *Galoglychia* colonized the Neotropics and Afrotropics, respectively, and (3) at ~23.05 (18.83–28.06) Mya, section *Adenosperma* colonized Australasia (Supplementary Fig. 3).

However, using the same nuclear genome dataset, analyses using a Bayesian concordance approach (BCA)^42^ suggest an alternative topology for the deepest node of *Ficus* (Supplementary Fig. 1E and Appendix 2). The primary concordance tree (PCT tree, inferred with the program BUCKy^43^) instead suggests section *Pharmacosycea* is best supported as sister to all other figs^39^ (concordance factor (CF) = 0.406, meaning that 40.6% of genomic windows support this topology^44^), compared to any other single clade (Supplementary Table 5). Apart from the placement of the basal section, the remaining topological structure of the PCT tree is similar to those of the Astral trees (Supplementary Fig. 1). Divergence times and ancient biogeography inferred based on the PCT tree suggest that *Ficus* originated in either Eurasia or the Neotropics roughly 63.20 (58.70–71.15) Mya (Supplementary Figs. 2B, 3B and Appendices 3, 4), followed by a rapid (~1 My) divergence between the Neotropical section *Pharmacosycea* and all other figs (which dispersed to or originated in Eurasia). Machado et al.^23^ found support for a similar scenario for pollinating wasps.

Maximum likelihood (ML) trees of both chloroplast and mitochondrial genomes based on full genome sequences were highly supported at almost all nodes. However, both showed striking and significant differences from the nuclear phylogenies (Fig. 2, Supplementary Fig. 4, and Appendix 5), as well as from each other. Consistent with the PCT tree based on nuclear genomes, and with most previous *Ficus* phylogenies^20,26,38,39,41^, both organelle-based phylogenies strongly supported section *Pharmacosycea* (monoecious, Neotropical, passive pollination) as being sister to all other *Ficus*. Further, none of the six currently recognized subgenera of *Ficus* (subgenera *Urostigma*, *Pharmacosycea*, *Ficus*, *Sycidium*, *Synoecia*, and *Sycomorus*)^21^ was supported as a monophyletic clade by either of the two organelle-based phylogenies^38^.

Analyses based on the Astral tree and PCT tree both support the interpretation that all main clades of *Ficus* co-occurred sympatrically during portions of their biogeographical histories. However, Astral and PCT trees suggest different clades as being the sister group to the rest of *Ficus* (subgenus *Urostigma* and section *Pharmacosycea*, respectively). Given its strong agreement with PhyloNetworks^45^ analyses (see below) (Supplementary Fig. 6), we will primarily focus on the inferences based on the Astral tree topology for the remaining analyses. However, similar analyses were also conducted based on the alternative PCT tree topology and yielded similar results that suggest prevalent hybridization events.

### Detecting hybridization and distinguishing it from ILS

In order to detect potential hybridization events, and distinguish these events from other processes such as ILS that potentially produce similar genetic signatures^44–48^, we first conducted ABBA-BABA *D*-statistics analysis^46,47^. We conducted these analyses for four-taxon groups hierarchically, from the species tips (fine scale) to the main clades (coarse scale) of the *Ficus* Astral species tree. These analyses yielded significant putative hybridization signals in most four-taxon groups across different phylogenetic/taxonomic levels (e.g., *Ficus* main clades, subgenus, sections, or species level) (Fig. 2 and Supplementary Data 1). All surveyed species, sections, subgenera, or main clades exhibited at least one hybridization event with other clades. Most inferred hybridization events occurred among groups that currently exhibit overlapping geographical distributions spanning Eurasia to Australasia. However, at least a few hybridization events were also identified between groups currently found on different continents: (i) Eurasian and Australasian sections and each of the exclusively New World sections *Pharmacosycea* and *Americana*; (ii) Eurasian and Australasian sections and the exclusively Afrotropical section *Galoglychia*; and (iii) *Galoglychia* and each of the New World sections (Fig.  2).

A second analytical approach, PhyloNetworks^45^, detected four hybridization events amogn *Ficus* clades (Supplementary Fig. 6 and Appendix 6). All four events were also supported by the ABBA-BABA *D*-statistics (Fig. 2, green lines). Notably, one ancient hybridization event was detected that suggested nearly 41% of the *Oreosycea* (monoecious) genome was transferred to the ancestor of the gynodioecious clade. The largest introgressive genomic portion of the gynodioecious clade detected with PhyloNetworks was also supported by the largest absolute *Z*-value (346.15) detected in ABBA-BABA *D*-statistics, from the hybridization event between the gynodioecious clade and the *Oreosycea* clade (Supplementary Data 1).

Similarly, Bayesian concordance analysis results with BUCKy^44,48^ also inferred frequent hybridization events (Supplementary Fig. 7 and Supplementary Data 2). Consistent with the other two methods, many of the hybridization events BUCKy detected appear to have occurred between clades from different subgenera, or even between different sexual systems (monoecious and gynodioecious) (Supplementary Fig. 7, Supplementary Data 2, and Appendix 2). We note that all 12 hybridization events detected by BUCKy were corroborated by results of ABBA-BABA *D*-statistics analyses (Supplementary Fig. 7 and Supplementary Data 1). In the BUCKy analyses, all *Ficus* main clades were inferred to be involved in at least one hybridization event, with the exception of section *Pharmacosycea*. We note that section *Pharmacosycea* (which the PCT tree suggests it sister to all other figs) currently exhibits an exclusively New World distribution.

### Phylogenetic incongruence among the three genomic compartments of *Ficus*

**Fig. 3:**
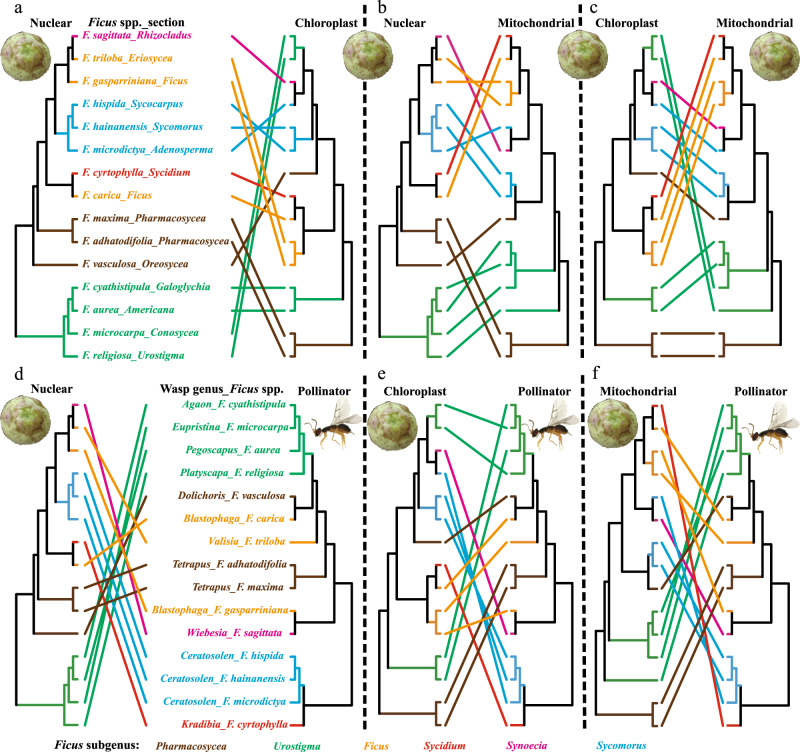
Phylogenetic incongrugence among fig genomic compartments and pollinator. Cophylogenetic comparison of each pair of phylogenies of three genomic compartments (**a**–**c**) and of the pollinators associated with the 15 fig species studied (**d**–**f**). *Ficus* and pollinator phylogenies used were indicated with symbol of figs and wasp separately. Tip-name colors indicate subgeneric classification. The pollinator phylogeny extracted from Cruaud et al.^20^ was used here. Extensive phylogenetic incongruence is shown for each pair of phylogenies. Refer to Supplementary Figs. 1, 4, and 5a and Appendices 1 and 5 for details on the phylogenies used here.

**Fig. 4:**
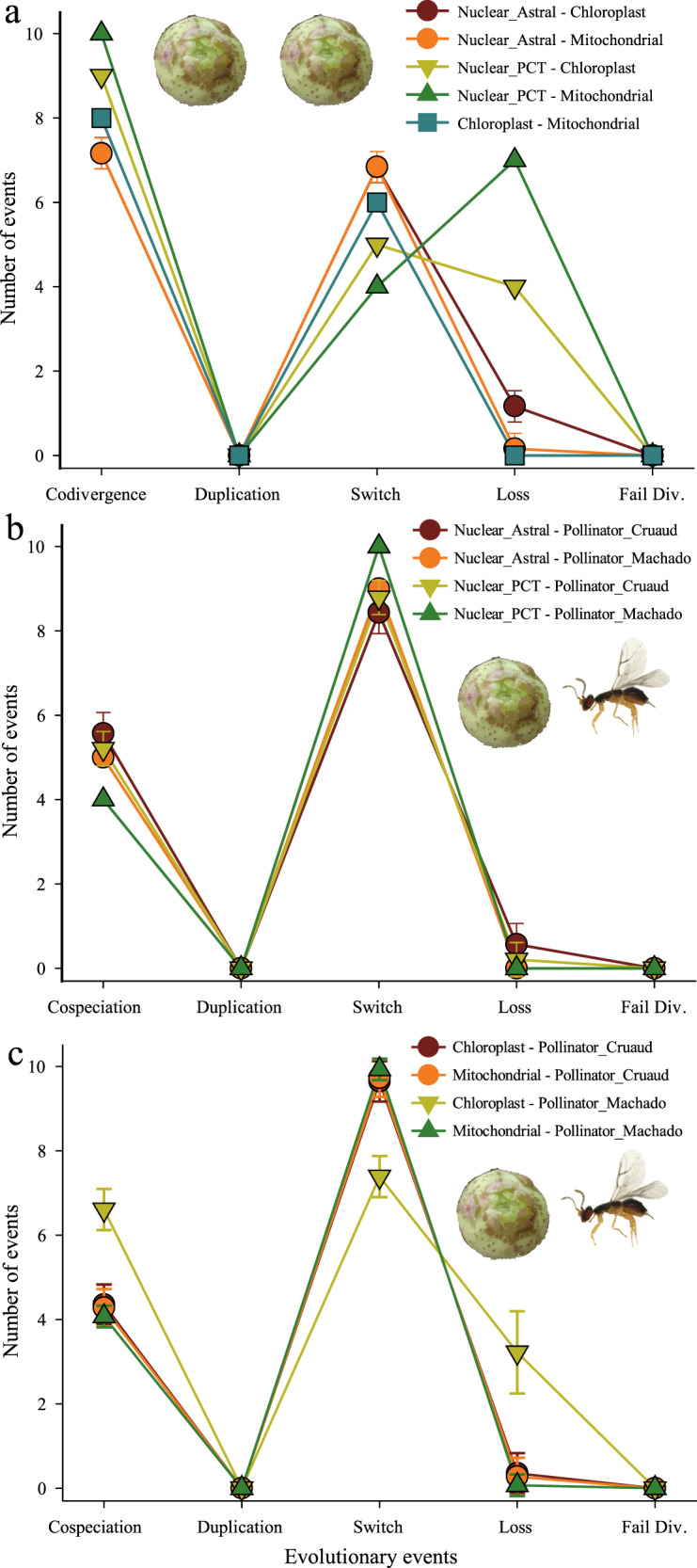
Frequency of co-phylogenetic evolutionary events inferred by JANE. Cophylogenetic analyses were conducted among phylogenies of the three genomic compartments (nuclear, chloroplast, and mitochondria) of *Ficus* (**a**) and then between these and genus-level phylogenies of the associated pollinators (**b**, **c**). *Ficus* and pollinator phylogenies used were indicated with symbol of figs and wasp separately. JANE takes the first tree as the “host tree”, and the second as the “parasite tree” shown in legends for cophylogenetic analysis. Means and standard errors were calculated with outcomes of all sets of solutions inferred with JANE under the same optimal cost. Lines linking numbers of different kinds of events are included for easy visualization; they add no quantitative information. Two *Ficus* nuclear phylogenies (Astral tree and primary concordance tree (PCT) in BUCKy) and two pollinator phylogenies^20,23^ were used. Five kinds of events were inferred: codivergence/cospeciation, duplication, associate switch (genomic switches or pollinator switches), associate loss (organellar genome loss or pollinator extinction), and failure to diverge. Refer to Source Data 2, Supplementary Figs. 5, 8–10, 12–15, Supplementary Table 7, and Appendix 7 for details.

**Fig. 5:**
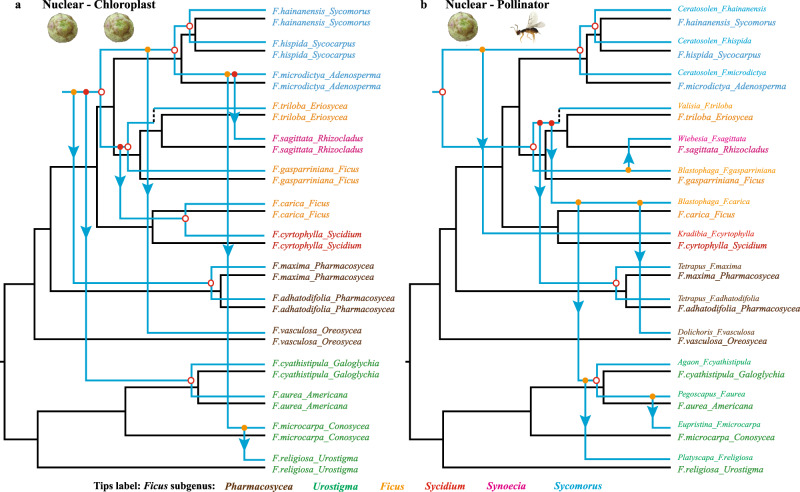
Co-phylogenetic reconciliations and detail evolutionary events inferred among main fig clades. One of the equally most parsimonious co-phylogenetic reconciliations inferred with JANE under optimal cost setting for each of two kinds of phylogeny pairs: between *Ficus* phylogenies based on nuclear and chloroplast genomes (**a**) and between *Ficus* nuclear-genome phylogeny and the phylogeny of associated pollinators (**b**). *Ficus* and pollinator phylogenies used were indicated with symbol of figs and wasp separately. The black tree represents the nuclear genomic phylogeny in both **a** and **b**, and the blue trees represent chloroplast phylogeny (**a**) or pollinator phylogeny extracted from Cruaud et al.^20^ (**b**). Tip labels indicate fig species and section in *Ficus* phylogeny, wasp genus, and associated host species in the pollinator phylogeny. Subgenus information (tip name color) of sampled species are also shown. Hollow red circles represent codivergence events in **a** and cospeciation events in **b**; solid colored circles and arrow lines represent events of associate switch (chloroplast switch) in **a** and of pollinator switch in **b**, while filled orange dots indicate existence of another potential switch location in the phylogeny that has equal cost, and a filled red dot means that all other potential switch locations have higher cost; dashed lines represent associate events of chloroplast loss (in **a**) or of pollinator extinction (in **b**). Refer to Supplementary Table 8 and Supplementary Fig. 5a for details.

Regardless of whether the Astral or PCT topology was used, we repeatedly detected significant phylogenetic incongruence among the nuclear, chloroplast, and mitochondrial genomes of *Ficus*. Therefore, the hypothesis of strict codivergence (i.e. that pairs of genomic compartments diverge at the same time and are continually associated) among them was clearly rejected (Fig. 3a–c). We then ran event-based cophylogenetic analysis using JANE 4.0 (ref. ^49^) to infer the relative contributions of different potential evolutionary events in generating phylogenetic incongruence (e.g., duplication, associate switches, associate losses, and failure to diverge). Notably, JANE 4.0 inferred high frequencies of both associate switch (suggestive of hybridization^50^, as organellar genome lineages switch among different background nuclear genome lineages) and associate loss events (“parasite lineage” [e.g., organellar genome] is associated with only one of two divergent “host lineages” [e.g., nuclear genomes], while another “host lineage” was associated with a different “parasite lineage”) (Fig.  4a, Supplementary Tables 6, 7, and Appendix 7). Furthermore, detailed analyses of the nuclear and the two organelle-based phylogenies uncovered frequent, relatively independent switching of both organelle genomes across all *Ficus* subgenera (Fig. 5a and Supplementary Figs. 8–10). This finding is consistent with previous demonstrations of biparental inheritance and heteroplasmy in plant organelles^50–52^. Finally, our analyses of the geographic distribution of each of the 59 fig species (Supplementary Data 3) included in Bruun-Lund et al.’s chloroplast genomic phylogeny^38^ showed an intriguing pattern. The chloroplast genomes of what appeared to be distantly related fig species from clearly different subgenera were frequently clustered together in the chloroplast genomic phylogeny. This unexpected pattern is particularly true for taxa that currently share the same geographic region. All of these findings are consistent with genetic exchange of organelles among lineages more likely to have experienced some level of sympatry (Supplementary Fig. 11).

### Pollinator host-switches and the evolutionary co-divergence of *Ficus* and fig wasps

Analogously to the incongruence found among fig nuclear and both organelle-based phylogenies, clear phylogenetic incongruences were also detected between the host figs and their associated pollinators (Fig. 3d–f). We conducted co-phylogenetic analyses using all combinations of the best supported Astral tree, the PCT tree and the two organellar phylogenies generated here for *Ficus* and the two most widely accepted (but substantially different) published fig wasp phylogenies^20,23^ (Supplementary Fig. 5 and Supplementary Note 3). Regardless of which of the two wasp phylogenies was tested against the various nuclear and organelle-based *Ficus* phylogenies, pollinator host-switch events were always inferred at higher frequency than co-speciation events (Fig. 4b–c, Supplementary Table 7, and Appendix 7). Further, reconciliation between any *Ficus* phylogeny and any pollinator phylogeny revealed that almost all *Ficus* main clades have been repeatedly involved in pollinator host-switching events (Fig. 5b and Supplementary Figs. 12–15). Meanwhile, almost all of the inferred pollinator host-switching events correspond to the hybridization events identified above (Fig. 5b, Supplementary Table 7, and Supplementary Note 4), and vice versa, most hybridization events among main clades (18 of 22 listed in Supplementary Table 8) also corresponded to one or more pollinator host-switching events.

## Discussion

The complete nuclear, mitochondrial, and chloroplast genomes for 15 species of figs (*Ficus* spp.) that we collected represent the most comprehensive genetic sampling thus far conducted for key taxa of the plant hosts in what is arguably the most extreme case of obligate pollinator mutualism known. Despite the depth of genetic sampling, analyses of these data do not unambiguously resolve the phylogenetic relationships among the recognized *Ficus* clades, especially at important basal nodes. What these complete genomic data do unambiguously indicate is that hybridization and introgression of components of the nuclear genome, and exchange of entire mitochondrial and chloroplast genomes, have likely characterized *Ficus* throughout its evolutionary history. Moreover, no published phylogeny of the corresponding pollinator wasp species (Hymenoptera: Chalcidoidea: Agaonidae) corresponds to any strongly supported nuclear or organelle-based phylogeny of their hosts. Frequent pollinator host-switching, rather than strict-sense cospeciation, was inferred as the predominant event along fig–fig wasp coevolution. Collectively, the existing data suggest evolutionarily frequent host switches of the pollinators that introduce new combinations of both nuclear and organelle genes. This process appears to take place even among distantly related hosts and throughout fig–pollinator wasp coevolutionary history (Fig. 2 and Supplementary Table 8).

Multiple evidences support prevalent hybridization has occurred throughout the evolutionary history of *Ficus*. Frequent hybridization events were inferred to have taken place within and across all six *Ficus* subgenera, based on all three different types of analyses applied to nuclear genome data: ABBA-BABA *D*-statistics^46,47^, PhyloNetworks^45^, and BUCKy^43,44^ (Fig. 2, Supplementary Fig. 7, and Supplementary Table 8). In particular, ABBA-BABA *D*-statistics^46–48^ suggest extensive hybridization events in all 15 sampled species (14 sections) (Supplementary Data 1). Moreover, all hybridization events inferred with PhyloNetworks and BUCKy were also corroborated by results of ABBA-BABA *D*-statistics (Fig. 2 and Supplementary Table 8). Interestingly, several hybridization events suggest the possibility of ancient “ghost” hybridization events involving an extant taxon and a now-extinct clade^47^ (Fig. 2 and Supplementary Figs. 6 and 7).

Further, neither the mitochondrial nor the chloroplast phylogenies are congruent with the nuclear species trees inferred by either ASTRAL or Bayesian approaches (BUCKy). They are not even congruent with each other (Figs. 3–5 and Supplementary Figs. 1 and 4). Both *Ficus* organelle genomes have clearly been switching nuclear backgrounds throughout their history (Fig. 4 and Supplementary Table 6), consistent with the results of Bruun-Lund et al.^38^, and in ways that suggest that hybridization can result in both maternal and paternal inheritance of the organellar genomes in *Ficus*. We note that biparental and paternal inheritance of organellar genomes, as well as organelle heteroplasmy, have been shown in an increasing number of angiosperms^50–52^. In addition, our analyses of the published chloroplast genomic phylogeny of 59 fig species^38^ revealed a geographic clustering pattern that often overrode expected similarity based on what subgenus the figs belonged to (Supplementary Fig. 11). This pattern suggests that hybridization among co-occurring species, even if they are otherwise distantly related, is the most likely mechanism accounting for the significant cyto-nuclear incongruence demonstrated in previous studies^32,38^. The simplest explanation consistent with all the above findings is that hybridization has occurred routinely throughout the evolutionary history of *Ficus*, and that its effects have been prevalent.

It is difficult to reconcile the complete genomic data for these 15 fig species with a standard biological species concept^53^, which assumes that species represent distinct gene pools through time, once speciation has occurred. In contrast, *Ficus* species may better fit the genic view of species, in which “species” exchange potentially large regions of the genome, except possibly those regions controlling adaptive traits important for reproductive fitness^54^. Meanwhile, our study also provides evidence of hybridization at the deepest nodes (50–80 Mya) of a species-rich group. Such demonstrations are rare, perhaps because of what were previously methodological limitations^55,56^. The *Ficus* hybridization events inferred here also help reconcile the previously encountered inconsistent phylogenetic reconstructions of *Ficus* that have been based on different morphological or molecular datasets, and analyzed with different methods that nonetheless assume separate gene pools^20,57^. Accordingly, this study highlights the utility of using multiple genomic compartments and multiple alternative analytical methods to distinguish between alternative phylogenetic hypotheses, and to corroborate inferences.

Pollinator host-switches could be a direct mechanism underlying *Ficus* hybridization events. Currently there are two main published and widely accepted molecular-based phylogenies of fig pollinating wasps^20,23^ that correspond to our sampled host fig taxa. Although neither of these phylogenies is based on data with comparable depth to the whole genome data generated here for key *Ficus* taxa, they represent the two currently most plausible, if alternative, views of fig wasp evolutionary history (Supplementary Fig. 5 and Supplementary Note 3). In each case, the data for both partners are sufficiently resolved to reject the hypothesis that either of the wasp phylogenies corresponds to any of the fig nuclear or organellar-based phylogenies produced by our analyses (Figs. 4b, c and  5b and Supplementary Figs. 11–15). This observation strongly suggests that host switching on the part of the associated pollinator wasp species, even between distantly related host taxa, has occurred on a regular basis^33^ (see Supplementary Notes 3 and 4 for details). Further, each phylogenetic hypothesis makes specific predictions concerning the evolutionary history of sections of *Ficus* and their associations with corresponding genera of pollinator wasps. For example, specific hypothesized host-switching and hybridization events potentially provide explanations for recent findings of cyto-nuclear incongruence^32–34,38,58^.

Importantly, the frequent pollinator host switches detected throughout *Ficus* evolution potentially provide the mechanism behind the prevalent inferred hybridization. All pollinator host-switching events throughout the coevolution of *Ficus* and agaonid wasps, as inferred through cophylogenetic analyses (between the most strongly supported Astral fig tree and the most recently published wasp tree^20^) have been matched to hybridization events detected between different host fig groups (Supplementary Table 7). Similarly, almost all (18 of 22 listed in Supplementary Table 8) identified hybridization events among main clades of *Ficus* have corresponding host-switching events. These results are also consistent with the inferred pollinator host-switching events and potential genetic exchange in studies of the relationships among closely related sympatric figs in Neotropical section *Americana*^33,34^ and in African section *Galoglychia*^32^.

Host-switching as a mechanism underlying hybridization at current ecological timescales is supported by experiments in which pollinator wasps are introduced to figs that are not their natural or most common hosts^37,59,60^. Even among distantly related hosts, hybridization is known to result in viable hybrid seeds, seedlings, and in some cases, adults^21,22,31–34,36,37^ (Fig. 2 and Supplementary Data 1). Notably, Ramirez^36^ documented hybridization events between introduced *F. religiosa* and native *F. aurea*. These species are included in our genomic and phylogenetic study and belong to different sections within subgenus *Urostigma*. Perhaps more remarkably, he documented viable hybrids between *F. religiosa* and *F. septica*, which belong to different subgenera (the latter belonging to subgenus *Sycomorus*), and exhibit different sexual systems (monoecious and dioecious, respectively). Both cases studied by Ramirez involved visits by pollinators of a native fig species into figs of another species introduced into its range by humans (*F. religiosa*)^36^. These and other^31,37^ examples support the plausibility of our phylogenetic and genomic inferences of hybridization between distinct figs from these same sections and subgenera (Fig. 2 and Supplementary Data 1).

Further exploration of the mechanisms of hybridization requires more evidence on how pollinators recognize the signaling of hosts. It is well established that the “appropriate” pollinator wasp species usually recognize and pollinate the “appropriate” host fig species (those in which they are able to reproduce)^61^. Increasingly, it is recognized that receptive figs produce distinctive blends of volatile compounds that mediate the degree of pollinator-specificity that is observed, usually contributing to reproductive isolation of *Ficus* species^25,39,62^. Determining what compounds (or combinations of compounds) of the host volatile chemical blends are recognized by the wasps and play important role in host recognition is critical^39,63,64^. What is known is that these blends differ in compound composition or in relative proportions of a large suite of shared compounds, many of which serve as well-characterized pollinator attractants across flowering plants^64,65^. What is also known is that fig wasps of a given species sometimes arrive, pollinate, and oviposit in the figs of hosts that are neither their most commonly associated host, nor even necessarily a particularly closely related one^22,28–31,36,37^.

Importantly, the interplay of the host chemical signals and the wasps’ recognition of these signals fundamentally directs gene flow among conspecific and sometimes hetero-specific fig individuals by determining what the wasp recognizes as an “appropriate” host^22,30,61^. One of our previous studies^22^ included *F. hainanensis* (one of the 15 species sampled in the present study) and four other figs of subgenus *Sycomorus*. We found that those fig species with the highest rates of “mistake visits” to their receptive figs by wasp species other than their most common pollinator, emitted volatile blends that were similar to those of the “common” hosts of those “mistaken” wasps. We also found that hybrid trees occurred most frequently between those fig species with the most similar chemical blends. Given that the genetics underlying both fig volatile production and wasp volatile recognition are directly affected by the wasps’ choice of host, the potential for hybrid figs to exhibit markedly different volatile chemical signatures is not only possible, but likely^66^. In addition, shifts in volatile chemistry produced by hybrids and resulting interactions with the wasps that they subsequently attract potentially initiate rapidly evolving feedbacks between the hosts and their pollinators. These feedbacks could isolate hybrids from parental lineages, and contribute to the generation of additional species diversity in both host and wasp^14,15,66–68^. How hybridization influences blends of volatile compounds, and how pollinator fidelity to a new host is established, as well as how chemical signaling by the host figs and recognition by the associated wasp pollinators vary over geographical scales, are critical gaps in our understanding of how hybridization might drive diversification of fig–fig wasp systems. As in a case reported in orchids^68^, hybrid figs likely create new volatile blends, with the subsequent establishment of new pollinator specificity via additional pollinator host-switches.

Our results also offered insights into the temporal and biogeographical reconstructions of the fig–wasp mutualism. All current evidence relevant to *Ficus* biogeographic history indicates that figs and wasps have been associated in a pollination mutualism for a long time (roughly 60–90 Mya)^20,23,69^. Some evidence suggests that the mutualism originated in Eurasia^20^, whereas other evidence suggests a Gondwanan origin^23,69^. All current genetic evidence and all analyses point to one or the other of the two monoecious clades of figs (subgenera *Urostigma* and *Pharmacosycea*) as being sister to the rest (mainly gynodioecious groups) and thus to a single derived origin of gynodioecy in figs^39^. The Astral species tree of figs based on nuclear genomes, and one of the wasp phylogenetic reconstructions^20^, are most consistent with a scenario of an Old World origin, with the extant subgenus *Urostigma* as the basal clade followed by divergence of subgenus *Pharmacosycea* (Old World section *Oreosycea* and New World section *Pharmacosycea*), and the subsequent dispersal of the latter section to what is now the New World. This scenario was also supported by the topology of *Ficus* inferred with PhyloNetworks (Supplementary Fig. 6). According to this scenario, the basal divergence of subgenera *Urostigma* and *Pharmacosycea* was followed by the divergence of the Old World gynodioecious species, and a colonization of the monoecious fig species by wasps that were associated with gynodioecious species (Figs. 1 and  5b).

Alternatively, the PCT tree, both organellar phylogenies, and one of the earlier wasp phylogenies^23^, are more consistent with section *Pharmacosycea* (currently New World) and its associated pollinators (the passively pollinating genus *Tetrapus*) being sister to the rest of the figs and the rest of the pollinator wasps, respectively. Under this scenario, a Gondwanan origin would have been followed by a separation of this currently New World group from the rest, with a later colonization of the New World by a group of strangler figs (section *Americana*) that are most closely related to the African section *Galoglychia*. This scenario also differs from the other in being compatible with the position of the passively pollinating *Tetrapus* wasps as being basal to all other pollinating wasps^23^. Importantly, both scenarios clearly indicate a single origin of gynodioecious species from the monoecious fig clades (subgenus *Urostigma* or *Pharmacosycea*)^39^.

Additional genomic data from subgenera *Urostigma* (particularly from the section *Malvanthera* from Australia) and *Pharmacosycea* and their associated pollinators, as well as fossil evidence (e.g., from South America, Antarctica, Australia, and India), will be needed to more clearly resolve the issue of which of these current taxa represent the basal groups of figs and wasps. Certainly, there is abundant evidence to support hybridization among even distantly related fig taxa that currently co-occur, and at least some evidence for hybridization among groups that currently do not. The latter is most likely to have occurred during geological periods when the different groups did co-occur. Considering the prevalent hybridization within genus *Ficus* inferred here and the challenges that widespread hybridization presents to current standard methods of species tree inference, future work on phylogenetic relationships and biogeographic history of *Ficus* should explicitly consider the potential effects of hybridization.

In conclusion, we suggest that future studies attempting to reconstruct the phylogenetic relationships between figs and their pollinators should not be constrained by the idea that species or even lineages of pollinators have strictly co-radiated with the currently corresponding species or sections of figs. The rampant hybridization that our complete nuclear, mitochondrial, and chloroplast genome data reveal and substantiate helps to explain why resolving fig phylogenies and fig–wasp co-radiation has been so difficult and controversial^20,21,23,38,57,69,70^. In conjunction with the need for improving wasp phylogenies at large taxonomic scales and for wider sampling from key portions of the fig lineages (e.g., the basal separation of *Urostigma* and *Pharmacosycea*), the most relevant questions shift from “Which lineages of wasps are associated with which lineages of figs?” to “What parts of the fig genome have descended together and what parts have been introduced during different hybridization events?” The timing, location, and genetic consequences of hybridization events, and their roles in adaptation, trait evolution and diversification of the two partners, should become the targets of more in-depth studies. In short, little in the evolution of figs and their wasps appears to make sense except in the light of hybridization and introgression (mediated by pollinator host-switches).

Similar diversification scenarios, with both pollinator specificity-mediated reproductive isolation and pollinator host switch-mediated hybridization, have potentially occurred in other “strict-sense” obligate pollination systems. For example, in Phyllanthaceae-*Epicephala* moth systems, sexually deceptive orchids, and oil-collecting bee–orchid systems, plants are also characterized by high, but not strict-sense, pollinator specificity and by relatively weak post-mating isolation mechanisms, as well as by frequent pollinator host-switching and hybridization^14,16,18^. We suggest that the genomic patterns of hybridization and introgression that we have documented in *Ficus* will help guide future studies of the processes that influence diversification in other obligate and generalist plant-pollinator systems.

## Methods

### Study design

The analytical frameworks applied included: (1) whole-genome short-read sequencing of 17 species representing all major *Ficus* clades and two outgroups, mapping the reads to the reference genome assembly (*F. microcarpa*)^39^, and de novo assembly of chloroplast and mitochondrial genomes based on the same set of samples; (2) inference of phylogenies of the three genomic compartments of *Ficus*, and reconstruction of biogeographic history; (3) detection of hybridization with ABBA-BABA *D*-statistics, PhyloNetworks and, BUCKy based on nuclear genomic data; (4) reconciliation of phylogenetic incongruence among nuclear, chloroplast, and mitochondrial genomes, and inference of the potential evolutionary events causing the incongruence by mapping of the geographic distribution of species and haplotypes based on a previously published 59-species chloroplast genomic phylogeny^38^; (5) reconciliation of phylogenetic incongruence between current fig phylogenies and the two most widely accepted published pollinator phylogenies^20,23^, to infer the relative contributions of pollinator host-switching events and cospeciation events to fig diversification.

Fifteen fig species representing all major *Ficus* lineages (14 sections of all six subgenera)^21^ and two outgroup species, *Morus alba* and *Antiaris toxicaria*, were sampled in this study (Supplementary Table 1). Samples were obtained from living adult trees from the outdoor plant collection (*Ficus* garden) of Xishuangbanna Tropical Botanical Garden, Chinese Academy of Sciences (XTBG, CAS), or from their natural habitats nearby (more than 50 *Ficus* species occur naturally in the area^71^) and in other countries (*F. maxima* and *F. adhatodifolia* from Brazil and *F. microdictya* from Papua New Guinea). One species, *F. carica*, was obtained from the Kunming Institute of Botany (KIB, CAS). All trees sampled from the XTBG plant collection originated from seeds or seedlings collected from their natural forest habitat nearby or introduced from other countries. All trees were carefully identified based on morphological traits before sampling. Meanwhile, our sample of species is also taxonomically and geographically well representative based on current classification and distribution (in species and section level)^20,21^. Pollinator phylogenies (Supplementary Fig. 5) were extracted from two published fig wasp phylogenies^20,23^.

### Genome sequencing and SNP genotyping

Whole genomic DNA was extracted from ~100 mg of silica gel-dried fresh leaf tissue using a modified cetyltrimethylammonium bromide method^72^. A minimum of 5 μg of total genomic DNA from each sample was used to construct an independent sequencing library following the manufacturer’s instructions, before sequencing using the Illumina HiSeq X Ten (Illumina Inc.). To avoid potential DNA contamination during sequencing, such as that via index-swapping, we constructed dual-indexed libraries with unique indexes for each sample. The dual indexes contained a total of 16 base pairs (bp) and were inserted into the flanking regions of the target DNA fragments. Raw reads that had any mismatch with index sequences were clustered as undetermined sequences and removed from our analysis. Meanwhile, the unique dual-index of each sample also ensured unambiguous separation of raw sequenced data and avoidance of potential sequences contamination among samples.

Adaptors and low-quality bases were removed from the raw pair-end reads using Trimmomatic-v0.39 (ref. ^73^) after performing quality control with FastQC-v0.11.9 (ref. ^74^). The reads were filtered with a sliding window of size 7, with an average Phred score = 20 within each window. The trimmed reads were mapped to a high-quality *F. microcarpa* reference genome^39^ assembly with 93.5% BUSCO completeness (submitted to GSA database (http://gsa.big.ac.cn/) under accession number GWHABKV00000000, Supplementary Note 1) using BWA-v0.7.17 (ref. ^75^) with default parameters. The mapped reads were sorted, and duplicated reads were removed using SAMtools-v1.9 (ref. ^76^). The Realigner Target Creator and Indel Realigner programs from the Genome Analysis Toolkit (GATK-v4) package^77^ were used for global realignment of reads around indels from the sorted BAM files.

To identify a high-quality SNP dataset, we applied the GATK pipeline following the suggested best practices workflow (https://gatk.broadinstitute.org/hc/en-us/articles/360036194592-Getting-started-with-GATK4). The GATK best practices included a number of steps to ensure variant accuracy. To remove erroneous mismatches around small indels, IndelRealigner was applied to process the alignment of BAM files. HaplotypeCaller was used to call variants for each sample and the program GenotypeGVCFs was used to merge all of the individual GVCF (Genomic Variant Call Format) files. The merged GVCF file was subject to quality control so that the minimum quality of each SNP was 30. To avoid false positives of read mapping in repetitive sequences, sequencing depth of each high-quality SNP ranged from 2 to 200. A total of 4,473,644 SNPs were used to construct three non-overlapping size-fixed windows, with window sizes of 50, 100, and 500 kb. A second, more conservative SNPs calling strategy was also used. In this strategy, samtools/bcftools was applied to call SNPs using the same data with default parameters. SNPs that were present in both the GATK and the samtools/bcftools datasets were used for further analysis. In addition, we allowed each SNP site with a maximum missing rate of 40% across all samples tested. This ensured that the two outgroup species (*M. alba* and *A. toxicaria*) supplied abundant information for inference of the phylogenetic tree. A total of 389,834 SNPs in non-repeat regions were extracted from the VCF data, using a home-made PERL script (https://github.com/tangerzhang/VCFplayer/blob/master/vcf2fasta.pl). Based on the second SNPs dataset (using samtools/bcftools), we used a sliding-window approach and constructed a SNPs-fixed window with window size of 1000 SNPs and a step size of 1100 SNPs, meaning a space of 100 SNPs between two successive windows. To maintain abundant information within each window in the above four windows datasets, we removed windows with fewer than 50 SNPs, and generated 351, 764, 3697, and 3459 windows for the 1000SNP, 500-kb, 100-kb, and 50-kb datasets, respectively (Supplementary Table 3). The physical location of each window in chromosomes of the reference genome was labeled (Source Data 1). All four datasets were used to infer species trees with ASTRAL-II (ref. ^40^); the 1000SNP dataset was also used for BUCKy-v1.4.4 analysis^43^; and the 500-kb datasets were further used for PhyloNetworks analysis^45^.

Both chloroplast and mitochondrial genomes were de novo assembled with the recommended pipeline of the GetOrganelle toolkit^78^. Chloroplast genomes of all 17 species and mitochondrial genomes of 14 species were assembled in circular sequences. For three species whose mitochondrial-genome assemblies were not circular, their assembly graphs were checked using Bandage program^79^, and non-target contigs were manually cleaned. The cleaned assembly graphs of circular genomes or contigs were exported to FASTA sequences using the GetOrganelle toolkit (Supplementary Table 2 and Appendix 5). SNPs in chloroplast and mitochondrial genomes from the same sequencing raw data were identified using the GATK pipeline as mentioned above. In total, we identified 5486 SNPs in chloroplast genomes and 4649 SNPs in mitochondrial genomes (Supplementary Table 2). All sampled species are inferred as diploid species based on ploidy estimation with nQuire program^80^ (Supplementary Table 9).

### Reconstruction of *Ficus* evolutionary history

We inferred *Ficus* phylogenies based on the nuclear, chloroplast, and mitochondrial genomes of the target figs with multiple methods separately. We first inferred the Astral species trees under a multi-species coalescent model with the four genomic-windows datasets, using the ASTRAL-II program^40^. For each of the four windows datasets, gene trees of all windows were inferred using the RAxML-v8 program^81^ with GTR+GAMMA substitution model and 1000 bootstrap replications. The Astral species tree was inferred with greedy consensus^40^ of the 1000 bootstrapped replicate trees of each gene tree, and both support values (showing the percentage of bootstrap replicates that contain a given branch) and branch length in coalescent units were inferred (Supplementary Fig. 1).

We also inferred the PCT tree of *Ficus* under the BCA-based^42^ BUCKy^43^ with the 1000SNP dataset. Whereas the coalescent-based Astral tree assumes that ILS is the sole reason for incongruence in gene trees, the BCA method makes no such assumption and allows both ILS and hybridization as causes of incongruence. The PCT tree with the highest CF value was reconstructed to summarize the main vertical phylogenetic signal (i.e., the best-supported tree branching pattern) of all gene trees based on all single windows. The CF value gives the proportion of genomic windows (or genes) supporting a given clade^44^. In brief, we first ran Bayesian analysis of each individual gene (window) with MrBayes-v.3.2.5 (ref. ^82^) to obtain a posterior distribution of gene trees. For each gene tree inference, under the GTR+GAMMA substitution model, two Markov chain Monte Carlo simulations were run with one cold chain and three heated chains for chain lengths of 1,000,000 (10% burn-ins) and sampling every 100 generations. Finally, we used BUCKy to estimate the PCT tree and CF values of different splits.

*Ficus* divergence time was estimated using the Bayesian inference in the MCMCTREE program of PAML 4.9a^83^, based on the topology of the Astral *Ficus* species tree using the 100-kb dataset, which was for the most part corroborated by the other Astral trees (Supplementary Fig. 1). MCMCTREE was used to estimate the branch length with datasets of 10 partitions of the whole aligned genome^84^ (Appendix 3). To decide the rate prior, we first ran BASEML to estimate the overall substitution rate under a strict molecular clock with the root age (Crown Moraceae) set to 93 Mya^85^ for a rough estimation. We set rgene_gamma = 1, 2, with average 0.5 × 10^−8^ substitutions per site per year. We then estimated the divergence times and 95% credible interval for each node with approximate likelihood principle^86^ under HKY85 model, and with independent rates model (clock = 2). Seven fossil calibrations following contemporary accepted standards on Moraceae and *Ficus*^20,85^ (Supplementary Table 4) were placed on the phylogeny using soft bounds^87^. The MCMC analyses ran 200,000,000 iterations with sampling every 10,000 cycles after a burn-in of 200,000. The analyses were run twice to ensure chain convergence with the effective sample size (ESS) larger than 350, using Tracer v.1.6 (http://beast.bio.ed.ac.uk/Tracer).

To investigate the general biogeographical history of *Ficus*, we further reconstructed the ancestral geographic distribution of *Ficus* lineages using the R package “BioGeoBEARS”^88^. We categorized current species distributions into four states, Eurasia, Afrotropics, Australasia, and Neotropics, following previous studies^20^. We inferred the ancestral distribution probabilities of *Ficus* lineages based on the present distribution states of the 15 fig species, and the Astral species tree with fossil calibration (Supplementary Fig. 2). Maximum number of regions (of the four listed above) in which a lineage could be distributed was set as two. Six biogeographical models were used, including three models allowing jump dispersal events^88^. Choice of the final pattern of *Ficus* biogeographical history retained for analysis was based on results of the best-fit model (Supplementary Table 10 and Appendix 4). Following the same methodology, divergence time and ancient biogeography of *Ficus* plants were also inferred based on the PCT tree.

Phylogenies of the two organellar genomes of *Ficus* were also inferred separately based on the concatenated full genomic sequences (Appendix 5). The full sequences were automatically aligned in MAFFT version 7.0 (ref. ^89^) using the default option. The ML tree of chloroplast genomes was reconstructed using RAxML^79^ with the most appropriate substitution model (GTR+GAMMA) inferred using JmodeTest2 (ref. ^90^) and 1000 bootstrap replications, which is the same strategy that was used in a similar previous study^39^. Mitochondrial genomes showed high rates of reversion and translocations. The sequence orders were adjusted in MULAN (https://mulan.dcode.org/) using *Ficus adhatodifolia* (WG105) as the reference, aligned in MAFFT, then manually adjusted using Geneious version 11 (https://www.geneious.com). The alignment was trimmed using 10% gapping option for sites. The ML tree of the mitochondrial genomes was reconstructed using FastTree-2 with the GTR substitution model and a Gamma20-based likelihood option under an approximately-maximum-likelihood method^91^.

### Distinguishing hybridization from ILS

First, we used ABBA-BABA *D*-statistics^46,47^ to detect hybridization along a specific branch of the phylogeny, based on the frequencies of discordant SNP genealogies in a four-taxon tree. The method is based on the relative abundance of two allelic configurations called “ABBA” or “BABA”. Given four taxa, P1, P2, P3, and an outgroup O, with the relationship “[(P1, P2), P3], O”, ABBA patterns are SNPs where P1 retains the outgroup allele and P2 and P3 share the derived allele. Similarly, BABA patterns are SNPs where P1 and P3 share the derived allele and P2 retains the outgroup allele. Under the null hypothesis of no gene flow, the ABBA and BABA patterns should be equally frequent via ILS and the value of *D* should be equal to zero. A significantly positive *D* value indicates gene flow between P2 and P3, whereas a significantly negative *D* value indicates gene flow between P1 and P3 (ref. ^47^). Here, an improved *D*-statistic^46^ method calculated with the program doAbbababa2 implemented in ANGSD^92^ was used. Briefly, BAM files that were used for variant detection of all 17 species sampled were recruited for this analysis. All samples underwent error correction and ABBA-BABA *D*-statistics analysis was conducted at different taxonomic/phylogenetic levels according to the instructions on the ANGSD website (http://www.popgen.dk/angsd/index.php/Abbababa2). With these capacities, the improved *D*-statistics can obtain robust results even when sequencing depth is very low (1–10X)^46^.

To detect hybridization events throughout *Ficus* evolution, we conducted ABBA-BABA *D*-statistics for four-taxon groups hierarchically, from the species tips to the main clades following the topology of the Astral species tree. When *D*-statistics analysis was conducted in four-taxon groups at higher taxonomic/phylogenetic levels, all species within each taxon (section, subgenus, or main clade) were treated as sample replications (Supplementary Data 1). Only monophyletic higher-level clades or subgenera in Astral species trees were used (Supplementary Fig. 1). A non-*Ficus* species (*Morus alba*) was taken as outgroup in all tested four-taxon groups finally shown. The doAbbababa2 program could calculate the *D*-statistics for all potential four-taxon combinations of species or groups. This means that for each species or clade, one could potentially infer multiple hybridization events with other taxa if the species was included in multiple four-taxon groups. However, only those four-taxon groups that followed the phylogenetic topology of the Astral species tree were considered. In a few cases, results of some alternative four-taxon groups were also kept when the phylogenetic topology among taxa was unstable (Supplementary Data 1).

In addition, we inferred *Ficus* hybridization events using PhyloNetworks^45^, which infers phylogenetic networks with maximum pseudolikelihood from gene trees or quartet CF value. It allows both vertical inheritance under the coalescent model, and horizontal inheritance (e.g. hybridization) with reticulation nodes in the network. Following the online pipeline (https://github.com/crsl4/PhyloNetworks.jl/wiki), PhyloNetworks analyses were conducted with the 500-kb windows dataset. *Ficus* networks were inferred based on the ML gene trees generated with the 500-kb windows dataset. We ran SNaQ to search for the best network with the maximum hybrid node number (hmax) increasing from zero to 15, each hmax being repeated ten times. The optimal number of hybridization events was chosen when the negative log pseudolikelihood (−logplik) score of an additional hmax did not significantly decrease.

Furthermore, we also inferred *Ficus* hybridization events based on the analysis of results under BUCKy on inference of species trees based on the 1000SNP dataset (Appendix 2). The BCA-based species tree inference using BUCKy makes no particular assumption about possible causes of gene tree incongruence (e.g., ILS, hybridization, horizontal gene transfer). With the similar function of above ABBA-BABA *D*-statistics and PhyloNetworks, BUCKy provides a third way to distinguish hybridization from ILS by testing whether the distribution of gene-tree frequencies is compatible with that expected under neutral coalescent processes (ILS), or whether other biological processes must also be invoked (e.g., hybridization, horizontal gene transfer)^44,48^. For example, under a coalescent process, for a three-taxa group ((a,b),c), the most probable topology is (a,b),c), i.e., the topology that matches the species tree and has the highest frequency; and the two non-matching alternative minor topologies, (a,c),b) and (b,c),a), are expected to be equally frequent (ref. ^48^). However, if the frequencies of the two non-matching minor topologies are significantly different, the coalescent model can be rejected and the contribution to gene-tree incongruence of other biological processes additional to ILS, such as hybridization or horizontal gene transfer, can be inferred^44,48^. Under BUCKy, the CF value and its credibility interval for each clade can be estimated, indicating the proportion of genes supporting the clade^44^. The clades with the largest CF values produce the PCT tree, and non-overlapping CF credibility intervals of two corresponding alternative minor clades can be used as evidence of hybridization in addition to ILS.

### Cophylogenetic analyses and detection of associate switches

Phylogenetic incongruences between nuclear and organellar genomes of *Ficus*, and between figs and their pollinating wasps, could result from hybridization and pollinator host switches. Event-based cophylogenetic reconciliation analyses were conducted using the program JANE 4.0 (ref. ^49^) to infer phylogenetic incongruences based on five types of evolutionary events: co-divergence/cospeciation, duplication, associate switches, associate losses, and failure to diverge. Incongruence between nuclear and organellar genomes, especially the associate (host-)switch events, indicates organellar genome exchange among *Ficus* taxa with different nuclear genomes, indirectly supporting the occurrence of hybridization between *Ficus* species. Host-switch events inferred between fig and pollinator phylogenies indicate pollinator host switches among *Ficus* taxa. Considering the close biological relationship between the fig/pollinator wasp interaction and *Ficus* hybridization, detection of corresponding host switches in both organellar genome and pollinator wasps between the same pair of *Ficus* taxa would strongly suggest that pollinator host-switches generate *Ficus* hybridization. The cophylogenetic reconciliations included those between all pairs of fig phylogenies based on nuclear, chloroplast, and mitochondrial genomes of the 15 fig species generated here, and phylogenies of their associated pollinating wasps extracted from previous studies^20,23^. JANE reconciles two phylogenies by minimizing the total costs with a certain cost setting for each of the five kinds of events listed above. Results of the cophylogenetic analyses depend on the cost weights assigned to different evolutionary events. We thus first selected the optimal cost setting by running both random tip mapping and random parasite tree permutation tests, with the cost range of each evolutionary event varying from 0 to 1 (except for associate switch events, which vary from 0 to 2). The genetic algorithm parameters were set following a previous study^20^, with generation number = 40, population size = 1000 and sample size = 100. The optimal cost settings, with *P*-value differing significantly from the random setting and with lowest observed cost value, were chosen for formal cophylogenetic analyses (Supplementary Table 8), which were then run in solve mode to find the best solutions to reconcile each pair of phylogenies under optimal cost settings and using the same genetic algorithm parameters.

Results of cophylogenetic analyses are highly dependent on the phylogenetic topology. To obtain more robust results, two alternative nuclear trees (Astral species tree and PCT tree) (Supplementary Fig. 1B, E), ML trees of the chloroplast and mitochondrial genomes (Supplementary Fig. 4), and two alternative ML trees of associated pollinator wasps (Supplementary Fig. 5) were used. Although neither of the phylogenies of pollinator wasps is as robust as those for *Ficus* generated here, they represent two alternative views of fig wasp evolution that are both considered plausible^20,23^. The most notable difference between the two pollinator phylogenies is the identity of the earliest-diverging wasp clade. The pollinator phylogeny of Machado et al.^23^ treated *Tetrapus* wasps (whose hosts are the Neotropical section *Pharmacosycea*) as sister to all other pollinating wasps, a placement that better matches the PCT tree of *Ficus*, which places section *Pharmacosycea* as sister to all other figs. This latter pair of phylogenies (Machado et al.^23^ pollinators tree, *Ficus* PCT tree) reflects a long-held view of the evolutionary history of fig–fig wasp mutualism, notably the hypothesis that passive pollination is the primitive condition in figs and wasps. In contrast, in the pollinator phylogeny published by Cruaud et al.^20^, *Tetrapus* was inferred to lie in the middle of the wasp phylogeny, while section *Pharmacosycea* was also inferred to lie near the middle of the Astral *Ficus* species tree (Supplementary Note 3).

### Geographic clustering pattern in the chloroplast phylogeny of 59 fig species

If hybridization is the main reason for nuclear–organellar genome incongruence, we could expect that fig species with long histories of geographic co-occurrence may have undergone exchange of organellar genomes during hybridization. This would lead to geographic clustering of similar chloroplast genomes. To test this hypothesis, we surveyed 59 fig species for which a chloroplast genome phylogeny^38^ and information on geographic distribution were both available (Supplementary Data 3 and Appendix 9). These represented all six subgenera and most main sections. Taxonomic data and the distribution area of each species represented in both nuclear and chloroplast phylogenies were collected^20,21,38^ and mapped onto the nuclear and chloroplast trees using the R package ape-v5.3^93^, to investigate the hypothesized geographic clustering patterns in the chloroplast phylogeny.

### Reporting summary

Further information on research design is available in the Nature Research Reporting Summary linked to this article.

## Supplementary information

Supplementary Information

Peer Review File

Reporting Summary

Description of Additional Supplementary Files

Supplementary Data 1-3

## Data Availability

All data needed to evaluate the conclusions in the paper are presented in the paper, and its Supplementary information files. Reporting summary for this article is available as a Supplementary information file. All raw resequencing data produced for this project have been deposited at the Genbank with the BioProject number as PRJNA684963, and the Genome Sequence Archive BIG Data Center (http://bigd.big.ac.cn/gsa) under the accession number of CRA001150 separately. All other raw results related to all figures or tables are supplied as Appendix files, which are available at Dryad (10.5061/dryad.zcrjdfn7m)^94^. Source data are provided with this paper.

## Source data

Source Data

## Code availability

All codes needed to evaluate the conclusions in the paper are presented in the paper, Source Data, and GitHub (10.5281/zenodo.4308886)^95^.
