# Supplementary material for: Genomic evidence of prevalent hybridization throughout the evolutionary history of the fig-wasp pollination mutualism
Source: Nat Commun. 2021 Feb 2;12:718. doi: 10.1038/s41467-021-20957-3 (PMC7854680; doi:10.1038/s41467-021-20957-3)
Supplement: Supplementary file 1 — Supplementary Information [file 41467_2021_20957_MOESM1_ESM.pdf]

Supplementary Information for

Genomic evidence of prevalent hybridization throughout the evolutionary history of the fig-wasp pollination mutualism

Gang Wang[1†*], Xingtan Zhang[2,3†], Edward Allen Herre[4], Doyle McKey[5], Carlos A. Machado[6], Wen-Bin Yu[7], Charles H. Cannon[8], Michael L. Arnold[9], Rodrigo A. S. Pereira[10], Ray Ming[11], Yi-Fei Liu[12], Yibin Wang[13], Dongna Ma[14], Jin Chen[1,3*]

Contents:

Supplementary Notes

Supplementary Note 1: Assessment of *F. microcarpa* reference genome assembly

Supplementary Note 2: Sex system and pollination mode of *Ficus* plants

Supplementary Note 3: Pollinator phylogenies used for cophylogenetic analysis in JANE

Supplementary Note 4: Detailed pattern of pollinator host-switching events inferred with JANE based on *Ficus* Astral tree and Cruaud et al.'s pollinator phylogeny

Supplementary Tables and Data (supplementary data are provided with this paper in separate file)

Supplementary Table 1: Sample information for the 17 Moraceae species selected for genomic resequencing

Supplementary Table 2: Sequencing summary for the 17 Moraceae species

Supplementary Table 3: Summary of genomic-windows datasets used for inference of species trees

Supplementary Table 4: Information on fossil calibrations for *Ficus* divergence time estimation in MCMCTree

Supplementary Table 5: All potential basal *Ficus* inferred with BUCKy based on the windows1000SNPs dataset

Supplementary Table 6: Frequency of evolutionary events inferred with co-phylogenetic analysis in JANE

Supplementary Table 7. Pollinator host-switch events inferred with JANE based on *Ficus* ASTRAL tree and Cruaud *et al.*'s pollinator phylogeny[11], and related evidence for *Ficus* hybridization based on different methods

Supplementary Table 8. Summary of evidence for hybridization events among main clades of *Ficus*

Supplementary Table 9: Genome ploidy estimated with nQuire

Supplementary Table 10: Model selection results for reconstruction of ancestral distributions with R package BioGeoBEARS

Supplementary Data 1: Detailed results of four-taxon ABBA-BABA *D*-statistics tests for hybridization detected at different phylogenetic levels in *Ficus*

Supplementary Data 2: Major discordance in splits groups and inference of hybridization with BUCKy

Supplementary Data 3: Geographic distribution of 59 figs studied by Bruun-Lund et al. 2017

Supplementary Figures

Supplementary Fig. 1: *Ficus* species trees estimated based on nuclear genomic datasets

Supplementary Fig. 2: Divergence times in *Ficus* inferred with MCMCTree using Astral tree (A) and primary concordance tree (B) based on nuclear genomic datasets

Supplementary Fig. 3: Estimation of ancestral distribution of *Ficus* following Astral tree (A) and primary concordance tree (B) under best models with BioGeoBEARS R package

Supplementary Fig. 4: ML trees based on *Ficus* chloroplast and mitochondrial genomes

Supplementary Fig. 5: Phylogenies of pollinating fig wasps associated with the studied fig species used for co-phylogenetic analysis in JANE

Supplementary Fig. 6. *Ficus* phylogenetic networks inferred by PhyloNetworks based on 500kb windows datasets

Supplementary Fig. 7. Hybridization events within *Ficus* inferred with BUCKy

Supplementary Fig. 8: One of the equally most parsimonious reconciliations between *Ficus* nuclear (Left: Astral tree; Right: Primary concordance tree) and chloroplast phylogenies inferred with JANE

Supplementary Fig. 9: One of the equally most parsimonious reconciliations between *Ficus* nuclear (Left: Astral tree; Right: Primary concordance tree) and mitochondrial phylogenies inferred with JANE

Supplementary Fig. 10: One of the equally most parsimonious reconciliations between *Ficus* chloroplast and mitochondrial phylogenies inferred with JANE

Supplementary Fig. 11: Geographic clustering pattern of *Ficus* chloroplast genomes on the 59-species chloroplast phylogeny

Supplementary Fig. 12: One of the equally most parsimonious reconciliations between *Ficus* nuclear phylogenies (Left: ASTRAL tree; Right: Primary concordance tree) and the pollinator phylogeny of Cruaud et al. (2012) inferred with JANE

Supplementary Fig. 13: One of the equally most parsimonious reconciliations between *Ficus* nuclear phylogenies (Left: ASTRAL tree; Right: Primary concordance tree) and the pollinator phylogeny of Machado et al. (2005) inferred with JANE

Supplementary Fig. 14: One of the equally most parsimonious reconciliations between *Ficus* chloroplast phylogeny and pollinator phylogenies (Left: Cruaud et al. phylogeny (2012); Right: Machado et al. phylogeny (2005)) inferred with JANE

Supplementary Fig. 15: One of the equally most parsimonious reconciliations between *Ficus* mitochondrial phylogeny and pollinator phylogenies (Left: Cruaud et al. phylogeny (2012); Right: Machado et al. phylogeny (2005)) inferred with JANE

Supplementary Fig. 16: One of the equally most parsimonious reconciliations between two pollinator phylogenies inferred with JANE

Source data (provided with online version of this paper)

Source Data 1: Physical distribution of genomic sliding windows in chromosomes of the *F. microcarpa* reference genome, supporting Supplementary Table 3

Source Data 2: Data points of cophylogenetic evolutionary events inferred by JANE, supporting Fig. 4

Appendix files (Freely available from Dryad: https://doi.org/10.5061/dryad.zcrjdfn7m)

Appendix 1: Results for tree construction under ASTRAL

Appendix 2: Results of analyses under BUCKy

Appendix 3: Results for *Ficus* divergence times under MCMCTree

Appendix 4: Results for reconstruction of ancestral distributions of *Ficus* lineages under all six models in BioGeoBEARS

Appendix 5: Chloroplast and mitochondrial genomic sequences and ML phylogenies

Appendix 6: Results for hybridization detection under PhyloNetworks

Appendix 7: JANE analyses data and phylogenies used

Appendix 8: Chloroplast phylogeny of 59 figs studied by Bruun-Lund et al. 2017

Supplementary References

Supplementary Note 1: Assessment of *Ficus microcarpa* reference genome assembly

Chromosome-level assembly of the *Ficus microcarpa* reference genome was conducted with PacBio Simple-Molecule, Real-Time (SMRT) technology and chromatin conformation capture (Hi-C)-based scaffolding methods, followed by Illumina short read-based polishing. Briefly, ~80 × coverage of subreads was generated with the PacBio RSII platform and ~50 × coverage by short reads on the Illumina HiSeq X10 platform.

The quality of the *F. microcarpa* reference genome we assembled was assessed using multiple standard approaches. BUSCO analysis showed 93.5% of genome completeness. PASA-assembled transcripts based on RNA-seq reads were aligned to the reference genome and the result showed that more than 99.9% of transcripts could be aligned to the assembly with 99.88% single base accuracy for our assembly. In addition, 97.98% of illumina reads could be mapped to the reference genome, covering 99.23% of the assembled sequences, indicating a high-quality reference assembly. Detailed information on the *F. microcarpa* genome is presented in our another newly published paper[1].

Supplementary Note 2: Sex system and pollination model of *Ficus* plants

Both sex system (monoecy vs. gynodioecy) and pollination mode (passive vs. active pollination) are important reproductive traits that are closely related to the interaction between figs and pollinator wasps. Given the complexity of adaptations required for transitions between both of these traits, especially for the host fig (but also for the pollinator wasp), these traits are viewed by many researchers as important traits, useful in resolving phylogenetic relationships throughout the radiation of fig-fig wasp mutualism[2-5]. Monoecious figs have both female function (seed production) and male function (production of pollen and the pollen vector, pollinator wasps) within the same fig syconium (fig for short). In contrast, gynodioecious figs have seed and pollen dispersal functions separated on female tree and male trees, respectively[6]. They are thus functionally dioecious. Sexual specialization in gynodioecious figs directly influences the fitness of pollinator wasps, as pollinators can only reproduce in male figs[7]. Pollinator wasps either have pollen pockets and coxal comb for collecting and depositing pollen grains actively (active pollination), or they show no such morphological traits and behavior (passive pollination). Meanwhile, the anther-to-ovule ratio in actively pollinated figs tends to be low (< 0.16, reflecting higher efficiency of pollen dispersal), and higher (> 0.21) in passively pollinated figs[4,6,8]. Pollinator wasps benefit from active pollination in terms of larval development[3,9].

The simpler mode of passive pollination and simpler sexual system of monoecy are likely basal traits, and active pollination and gynodioecy derived traits for figs and wasps. However, it is clear that during the course of

Ficus evolutionary history there have been at least one reversal in both traits. The question becomes whether any basal lineages exhibiting basal characteristics still exist in extant *Ficus* taxa. Currently, some studies show active pollination as basal traits for the extant fig and wasp taxa[4,10-12]. In this study, Astral species trees based on nuclear data supported the pantropical subgenus *Urostigma* (monoecious, with both active and passive pollination) as sister to all other *Ficus* species. In contrast, the primary concordance tree (PCT tree) based on nuclear data and both mitochondrial- and chloroplast-based phylogenies support the neotropical section *Pharmacosycea* (monoecious, passive pollination) as basal to all other figs, as other studies have concluded[13-15]. Our results thus strongly support monoecy as the basal state of the sexual system of all extant taxa, but we consider the basal state of pollination mode in extant figs to be still an open question.

Supplementary Note 3: Pollinator phylogenies used for cophylogenetic analysis in JANE

Two phylogenies of pollinator wasps associated with the 15 studied fig species, extracted from published wasp phylogenies[12,14], were used in cophylogenetic analysis in JANE. The Machado et al.[14] tree is based on only one mitochondrial protein-coding gene (COI) fully sequenced for all 15 wasp species that is completely sequenced for all species considered. In contrast, the Cruaud et al.[12] tree is based on six genes (two nuclear protein-coding genes (EF1a, Wg); two mitochondrial protein-coding genes (COI, Cyt b), and two ribosomal genes (28S rRNA, 18S rRNA)) although not all genes were successfully sequenced in all of the 200 wasp species. The denser species sample and bigger gene matrix in the study by Cruaud et al.[12] should make their phylogeny relative more robust. Although neither of these phylogenies of pollinator wasps is as strong as those for *Ficus* trees generated here, they represent two alternative views, both considered plausible, of fig wasp evolution (Supplementary Fig. 5, Supplementary Table 6 (last line)). The most notable difference between them is which wasp clade is placed as sister to all others. Machado et al.[14] treated *Tetrapus* wasps (whose hosts are section *Pharmacosycea* in Neotropics) as sister to all other agaonids, a placement that better matches the primary concordance tree of *Ficus*, which places section *Pharmacosycea* as sister to all other figs. This pair of phylogenies represents a long-held view on the evolutionary history of fig-fig wasp mutualisms. However, in the phylogeny of Cruaud et al.[12], *Tetrapus* is inferred to lie in the middle of the phylogeny. In the Astral species tree for *Ficus*, section *Pharmacosycea* is also inferred as being placed near the middle of the tree.

Pollinators of three of the fig species studied here (*F. gasparriniana*, *F. vasculosa*, *F. triloba*) were not included in Cruaud et al.'s phylogeny[12]. However, their study included pollinators of fig species closely related to these three (*F. chapaensis*, *F. nervosa*, and *F. fulva*, respectively), each pollinated by a wasp belonging to the same

genus as the pollinator of the studied fig (*Blastophaga*, *Dolichoris*, *Valisia*, respectively). As the three pollinator genera are all monophyletic[12], and only the genus-level phylogenetic topology was used in JANE, these replacements do not influence the analyses of phylogenetic discordance between figs and pollinators.

Two pollinator clades associated with fig species we studied were not included in the phylogeny reconstructed by Machado et al. We inserted them artificially into that phylogeny, following their placement in the phylogeny of Cruaud et al.[12]. These were (i) a *Ceratosolen* clade (whose hosts are section *Adenosperma*, represented by *F. microdictya* in our study), added as sister to another *Ceratosolen* clade (hosts section *Sycomorus*, represented by *F. hispida*); and (ii) a *Blastophaga* clade (hosts section *Ficus*, represented by *F. carica*), added as sister clade to *Dolichoris* (hosts section *Oreosycea*, represented by *F. vasculosa*).

Supplementary Note 4: Detailed pattern of pollinator host-switching events inferred with JANE based on *Ficus* Astral tree and Cruaud *et al.*'s pollinator phylogeny

Results of cophylogenetic reconciliation based on the Astral tree for *Ficus* and the most recently published pollinator tree[12] (Fig. 5B) inferred nine pollinator host-switch events. In detail, one pollinator host-switch event is inferred to have occurred near the root of the wasp phylogeny, from subgenus *Sycomorus* to subgenus *Sycidium*; four other switch events are inferred around the middle of wasp phylogeny. One is inferred from the Old-World gynodioecious section *Eriosycea* (subgenus *Ficus*) to the Neotropical monoecious section *Pharmacosycea* (subgenus *Pharmacosycea*), another from section *Eriosycea* (subgenus *Ficus*) to the *F. carica*-clade (subgenus *Ficus*), a third from the *F. carica*-clade to one clade of subgenus *Urostigma* (section *Americana* - *Galoglychia* clades), and a fourth from the *Americana* - *Galoglychia* clades to section *Urostigma*. Finally, three other switch events are inferred near the terminal branches of wasp phylogeny: (i) within gynodioecious clades (from subgenus *Ficus* to subgenus *Synoecia*), (ii) within monoecious clades (subgenus *Urostigma*, from section *Americana* to section *Conosycea*) and (iii) from the gynodioecious *F. carica*-clade (section *Ficus*) to the monoecious section *Oreosycea* (subgenus *Pharmacosycea*) (Fig. 5B). All those inferred host-switch events match with corresponding hybridization events (Supplementary Table 7).

Supplementary Table 1: Sample information for the 17 Moraceae species selected for genomic resequencing.

Species	Subgenus	Section	Pollination mode	Sexual system	Distribution	Sources*	Voucher**
F. adhatodifolia	*Pharmacosycea*	*Pharmacosycea*	Passive	Monoecious	Neotropics	Brazil	V000013
F. maxima	*Pharmacosycea*	*Pharmacosycea*	Passive	Monoecious	Neotropics	Brazil	V000009
F. vasculosa	*Pharmacosycea*	*Oreosycea*	Active	Monoecious	Eurasia	XTBG	HITBC161822
F. aurea	*Urostigma*	*Americana*	Active	Monoecious	Neotropics	XTBG	V0000018
F. microcarpa	*Urostigma*	*Conosycea*	Active	Monoecious	Eurasia + Australasia	XTBG	HITBC0021833
F. cyathistipula	*Urostigma*	*Galoglychia*	Active	Monoecious	Afrotropics	XTBG	V000002
F. religiosa	*Urostigma*	*Urostigma*	Active	Monoecious	Eurasia	XTBG	V0000012
F. microdictya	*Sycomorus*	*Adenosperma*	Active	Gynodioecious	Australasia	PNG	-
F. hainanensis	*Sycomorus*	*Neomorphe*	Active	Gynodioecious	Eurasia	XTBG	V000011
F. hispida	*Sycomorus*	*Sycocarpus*	Active	Gynodioecious	Eurasia + Australasia	XTBG	V000005
F. triloba	*Ficus*	*Eriosycea*	Active	Gynodioecious	Eurasia	XTBG	V000003
F. carica	*Ficus*	*Ficus*	Passive	Gynodioecious	Eurasia	KIB	V000017
F. gasparriniana	*Ficus*	*Ficus*	Passive	Gynodioecious	Eurasia	XTBG	HITBC0021807
F. cyrtophylla	*Sycidium*	*Sycidium*	Passive	Gynodioecious	Eurasia	XTBG	V000006
F. sagittata	*Synoecia*	*Rhizocladus*	Active	Gynodioecious	Eurasia + Australasia	XTBG	V000004
Morus alba	-	-	-	Dioecious	Eurasia	XTBG	V000008
Antiaris toxicaria	-	-	-	Dioecious	Eurasia	XTBG	HITBC0021773

Notes: *: samples were collected from living trees in natural forest (Bragança, Pará state, Brazil (-1°03'E, -46°45'E), by Rodrigo A. S. Pereira; PNG: from Papua New Guinea (145°16′E, -05°44′N), by Simon T. Segar and Daniel Souto-Vilarós); and from the outdoor plant collection garden and nearby natural forest by Gang Wang in XTBG (Xishuangbanna Tropical Botanical Garden, CAS, China, (101°16′E, 21°55′N)) and KIB (Kunming Institute of Botany, CAS, China, (102°43′E, 25°2′N)). Fig classification

and traits follow Cruaud et al.[12]. All trees sampled from the plant collection originated from seeds or seedlings collected from their natural forest habitat nearby or introduced from other countries. Voucher**: all herbarium specimens listed are stored in the Herbarium of Xishuangbanna Tropical Botanical Garden, CAS., with detail samples information and photos could be accessed online (http://hitbc.xtbg.ac.cn/) with voucher number.

Supplementary Table 2: Sequencing summary for the 17 Moraceae species.

Sample		No. of			Nuclear			Chloroplast				Mitochondrial			
ID	Species	reads	Data size (Gb)	Raw coverage (X)	Mapping proportion (%)	No. of SNPs	SNP depth	No. of SNPs (bp)	SNP depth	Sequence length (bp)	Genome coverage (X)	No. of SNPs	SNP depth	Sequence length (bp) (No.contigs)*	Genome coverage (X)
WG105	*F. adhatodifolia*	10367748	5.6	13.0	90.05	1532594	7.5	5471	1061	159113	870.2	4461	189	566874	243.6
WG112	*F. maxima*	12698721	6.9	16.1	88.25	1776381	8.9	5449	634	160560	822.1	4423	165	459564	200.2
WG90	*F. vasculosa*	4949091	5.6	13.0	98.24	1012428	10.5	5481	1110	160599	840.9	4638	205	501313	260.2
WG69	*F. aurea*	11807427	6.8	15.8	91.6	847515	9.0	5455	1030	160524	988.5	4576	173	470163	183.7
Fm	*F. microcarpa*	111388996	33.6	78.1	98.77	245181	57.9	5483	1903	160968	724.7	4638	1925	446816	408.0
WG71	*F. cyathistipula*	5420958	6.7	15.6	94.98	748441	8.1	5457	415	160466	928.3	4637	99	357255 (9)	413.9
WG62	*F. religiosa*	8890025	6.6	15.4	89.15	947792	8.9	5443	520	160657	864.6	4220	99	455109	194.2
WG157	*F. microdictya*	10909891	5.6	13.0	91.96	1006532	9.5	5459	992	160535	751.4	4391	254	433874	341.4
WG16	*F. hainanensis*	12145408	5.3	12.3	94.47	402535	5.2	5464	435	160358	227.3	4633	88	439,685 (2)	148.2
FhF3	*F. hispida*	48726015	14.7	34.2	93.36	1949236	23.4	5479	2271	160341	944.8	4478	528	711536	157.2
WG80	*F. triloba*	5021215	6	14.0	91.94	1403462	10.1	5458	372	160332	986.2	4595	78	472114	266.2
WG116	*F. carica*	11441139	4.6	10.7	95.68	951875	7.4	5458	2171	160461	1053.3	4638	228	345350 (9)	100.8
WG78	*F. gasparriniana*	4305502	5.7	13.3	97.04	1112740	8.0	5476	728	160448	1017.7	4637	92	466433	148.9
WG72	*F. cyrtophylla*	4554936	5.6	13.0	97.25	1208126	8.7	5460	612	160493	951.5	4620	83	489306	218.6
WG86	*F. sagittata*	4432329	6.3	14.7	96.36	1194937	9.5	5454	494	160286	898.2	4632	145	475839	377.8
WG120	*Morus alba*	26287343	8.7	20.2	57.13	283863	20.8	4796	1313	160602	1044.8	2415	571	554366	471.3
WG61	*Antiaris toxicaria*	207892793	62.4	145.1	49.26	640799	58.8	4814	12460	161941	684.2	2524	2760	572717	116.9
**Mean (Ficus)**		17803960	8.37	19.5	93.94	1089318	12.8	5463	983.20	160409	858.0	4548	290	472749	244.2

NB: ***:** Number of contigs is shown in parentheses when mitochondrial genomes could not be completely assembled (circular).

Supplementary Table 3: Summary of genomic-windows datasets used for inference of species trees. Physical distribution of genomic sliding windows in chromosomes of the reference genome is shown in Source data 2.

Window type	No. of windows used	Window length (Kb)	Step length (Kb)	No. of SNPs/window	Notice
1000SNP	351	1003 ± 636 (1000 SNPs)	~1014 ± 636 (1100 SNPs)	1000 ± 0	Fixed number of SNPs
500kb	764	500 ± 0	500 ± 0	5852 ± 3651	Fixed windows size
100kb	3697	100 ± 0	100 ± 0	1220 ± 839	Fixed windows size
50kb	3459	50 ± 0	100 ± 0	658 ± 442	Fixed windows size

NB: Fixed number of SNPs indicates that each window is the genomic fragment including 1000 SNPs. Size of the windows can thus vary, while window size in each of the other three windows types is fixed.

Supplementary Table 4: Information on fossil calibrations for *Ficus* divergence time estimation in MCMCTree

Nodes	Max age (Mya)	Min age (Mya)	Reference
Crown of Moraceae	100	-	Gardner *et al*. 2017[16]
Crown of *Ficus*	90	60	Cruaud *et al*. 2012[12]
Stem of section *Urostigma*	-	43	Cruaud *et al*. 2012[12]
Stem of subgenus *Sycomorus*	-	34	Cruaud *et al*. 2012[12]
Stem of subgenus *Sycidium*	-	26	Cruaud *et al*. 2012[12]
Stem of subgenus *Synoecia*	-	26	Cruaud *et al*. 2012[12]
Stem of section *Americana*	-	22	Cruaud *et al*. 2012[12]

Supplementary Table 5: All potential basal *Ficus* inferred with BUCKy based on the windows1000SNPs dataset. Primary and alternative minor splits indicating all main potential basal clades of *Ficus* phylogeny. The mean and 95% credibility interval of the split in the primary concordance tree are shown. Gynodioecious clade: including all four subgenera dominated by gynodioecious species. Section *Pharmacosycea* obtained the highest CF values (0.406) as a signal clade. However, subgenus *Urostigma* or its sections (highlighted in bold) are also repeatedly inferred as the basal *Ficus* clade, although with CF values.

**Splits Type**	**Splits**	**Sample-wide mean CF (95% credibility)**	**Genome-wide mean CF (95% credibility)**	**Basal clades inferred**
Primary	{1,12,16|2,3,4,5,6,7,8,9,10,11,13,14,15}	0.406 (0.328, 0.459)	0.405 (0.317, 0.481)	Sect. *Pharmacosyea*
Minor	{1,2,3,4,5,6,7,9,10,11,12,14,15|8,13,16}	0.160 (0.128, 0.188)	0.160 (0.113, 0.211)	**Sect. *Americana* + *Conosycea***
Minor	{1,2,3,4,5,6,7,9,10,11,12,14|8,13,15,16}	0.120 (0.094, 0.148)	0.119 (0.079, 0.165)	**Sect. *Americana* + *Conosycea* + *Galoglychia***
Minor	{1,3,4,5,6,7,9,10,11,12,14|2,8,13,15,16}	0.111 (0.080, 0.148)	0.111 (0.068, 0.161)	**Subg. *Urostigma***
Minor	{1,2,3,4,5,6,7,8,9,10,11,12,14,15|13,16}	0.110 (0.080, 0.140)	0.110 (0.068, 0.156)	**Sect. *Conosycea***
Minor	{1,3,4,5,6,7,8,9,10,11,12,13,14,15|2,16}	0.069 (0.040, 0.100)	0.069 (0.032, 0.112)	**Sect. *Urostigma***
Minor	{1,3,4,5,6,7,9,10,11,12,14,15|2,8,13,16}	0.069 (0.043, 0.100)	0.069 (0.035, 0.111)	**Sect. *Urostigma* + *Americana* + *Conosycea***
Minor	{1,2,3,4,5,6,7,9,10,11,12,13,14,15|8,16}	0.060 (0.031, 0.085)	0.060 (0.026, 0.099)	**Sect. *Americana***
Minor	{1,2,3,4,5,6,7,8,9,10,11,12,13,15|14,16}	0.130 (0.097, 0.168)	0.130 (0.084, 0.184)	Sect. *Rhizocladus*
Minor	{1,2,8,12,13,15|3,4,5,6,7,9,10,11,14,16}	0.093 (0.066, 0.125)	0.093 (0.055, 0.140)	Sect. *Oreosycea* + Gynodioecious clade
Minor	{1,2,3,4,5,8,10,11,12,13,14,15|6,7,9,16}	0.071 (0.048, 0.097)	0.071 (0.038, 0.109)	Subg. *Sycomorus*
Minor	{1,2,5,8,12,13,15|3,4,6,7,9,10,11,14,16}	0.062 (0.040, 0.085)	0.062 (0.031, 0.100)	Gynodioecious clade

Minor	{1,2,12|3,4,5,6,7,8,9,10,11,13,14,15,16}	0.060 (0.040, 0.083)	0.060 (0.030, 0.096)	All *Ficus* excluding sect. *Pharmacosycea*

NB: 1: *F. maxima*; 2: *F. religiosa*; 3: *F. gasparriniana*; 4: *F. cyrtophylla*; 5: *F. vasculosa*; 6: *F. hispida*; 7: *F. microdictya*; 8: *F. aurea*; 9: *F. hainanensis*; 10: *F. carica*; 11: *F. triloba*; 12: *F. adhatodifolia*; 13: *F. microcarpa*; 14: *F. sagittata*; 15: *F. cyathistipula*; 16: *Morus alba*;

Supplementary Table 6: Frequency of evolutionary events inferred with cophylogenetic analysis in JANE. The optimal cost setting, frequency (mean ± SD) of each evolutionary event, and cost setting selection results in JANE 4.01 are given. Frequency of both "Duplications" and "Failure to diverge" events are zero for all phylogeny pairs, and thus do not appear in the table. The optimal cost setting for each phylogenies pair was chosen with both the significant **P**-value (two sided **T**-test) and lowest total cost value in both random tip mapping (*test1*) and random parasite tree permutation tests (*test2*). Two nuclear phylogenies of *Ficus* (Astral tree and primary concordance tree (PCT)), chloroplast and mitochondrial phylogenies produced by our analyses, and the two main published phylogenies of pollinator wasps[12,14] (Supplementary Fig. 5[11]) were used. Refer to Supplementary Figs. 7-9 and 11-14 for details of phylogenetic reconciliations. Events whose frequency was significantly greater than zero are presented in **boldface type.** Significant frequency of associate switch and/or associate loss events indicates that the pair of phylogenies in question cannot be explained by the strict-sense codivergence/co-speciation model.

**Host tree (*Ficus*)**	**Parasite tree (*Ficus*/Pollinator)**	**Optimal Cost setting**	**Codivergence/ Cospeciation**	**Associate Switches**	**Losses**	**Observed Cost**	**Expected Cost** ^{test1}	**P**	**Expected Cost** ^{test2}	**P**	**No. of solutions***
Nuclear_ Astral	Chloroplast	C0D1DH1L1F1	**7.16 ± 0.37**	**6.84 ± 0.37**	**1.16 ± 0.37**	8	11.64 ± 1.00	0.02	11.63 ± 0.99	0.02	12018
Nuclear_ Astral	Mitochondrial	C0D1DH1L1F1	**7.16 ± 0.36**	**6.84 ± 0.36**	0.16 ± 0.36	7	11.24 ± 0.84	0	11.24 ± 0.84	0	11013
Nuclear_ PCT	Chloroplast	C0D1DH1L1F1	**9.00 ± 0.00**	**5.00 ± 0.00**	**4.00 ± 0.00**	9	11.34 ± 0.98	0.04	11.35 ± 0.96	0.04	25160
Nuclear_ PCT	Mitochondrial	**C0D1DH2L1F1**	**10.00 ± 0.00**	**4.00 ± 0.00**	**7.00 ± 0.00**	15	21.85 ± 1.79	0	21.85 ± 1.75	0	3160
Chloroplast	Mitochondrial	C0D1DH1L1F1	**8.00 ± 0.00**	**6.00 ± 0.00**	0.00 ± 0.00	6	11.34 ± 0.93	0	11.60 ± 0.85	0	1918
Nuclear_ Astral	Pollinator_Cruaud	C0D1DH1L1F1	**5.57 ± 0.50**	**8.43 ± 0.50**	0.57 ± 0.50	9	11.36 ± 0.89	0.02	11.64 ± 0.82	0.02	7621
Nuclear_ Astral	Pollinator_Machado	C0D1DH1L1F1	**5.00 ± 0.00**	**9.00 ± 0.00**	0.00 ± 0.00	9	11.44 ± 0.85	0	11.50 ± 0.82	0.03	5030
Nuclear_ PCT	Pollinator_Cruaud	C0D1DH1L1F1	**5.21 ± 0.41**	**8.79 ±0.41**	0.21 ±0.41	9	11.39 ± 0.84	0	11.40 ± 0.82	0	10090
Nuclear_ PCT	Pollinator_Machado	C0D1DH1L1F1	**4.00 ± 0.00**	**10.00 ± 0.00**	0.00 ± 0.00	10	11.44 ± 0.95	0	11.46 ± 0.92	0	11969

Chloroplast	Pollinator_Cruaud	C0D1DH1L1F1	**4.35 ± 0.48**	**9.65 ± 0.48**	0.35 ± 0.48	10	11.24 ± 0.71	0.04	11.25 ± 0.70	0.04	10180
Chloroplast	Pollinator_Machado	**C0D1DH2L1F1**	**6.61 ± 0.49**	**7.39 ± 0.49**	**3.22 ± 0.98**	18	21.87 ± 1.78	0	21.65 ± 1.98	0	6306
Mitochondrial	Pollinator_Cruaud	C0D1DH1L1F1	**4.27 ± 0.45**	**9.72 ± 0.45**	0.27 ± 0.45	10	11.48 ± 0.81	0.03	11.40 ± 0.92	0.03	6516
Mitochondrial	Pollinator_Machado	**C0D1DH2L1F1**	**5.78 ± 0.57**	**8.22 ± 0.57**	**2.56 ± 1.14**	19	21.52 ± 1.75	0	21.58 ± 1.89	0	9076
Pollinator_Cruaud	Pollinator_Machado	C0D1DH1L1F1	**8.00 ± 0.00**	**6.00 ± 0.00**	**1.00 ± 0.00**	7	11.33 ± 0.84	0	11.33 ± 0.83	0	19291

NB: "C0D1DH1L1F1" indicates cost setting with zero cost for cospeciation and one for all other events, "C0D1DH2L1F1" indicates cost setting with zero cost for cospeciation, a cost of two for associate switches, and a cost of one for all other events. Results of cophylogenetic analyses between the two wasp phylogenies (bottom row of the table) just show that their phylogenetic topologies differ significantly, and do not indicate any other biological meaning. * indicate number of reconciliation solutions inferred by JANE under the optimal cost setting. Each solution with give an inferred value of each five event (codivergence, duplication, associate switches, associate losses, and fail to divergence). Most or even all solutions may show same pattern. Based this data, the mean and SD of each event under each phylogeny-pairs were calculated. Meanwhile, the reconciliation pattern confirmed with highest number of solutions were shown in supplementary figure 7-15.

Supplementary Table 7. Pollinator host-switch events inferred with JANE based on *Ficus* ASTRAL tree and Cruaud *et al.*'s pollinator phylogeny[12], and related evidence for *Ficus* hybridization based on different methods. There are three columns of direct evidence for hybridization detection, and 13 columns of indirect evidence based on cophylogenetic reconciliation using JANE, when associate switches were inferred to have occurred between related figs groups.

		Direct evidence			Indirect evidence												
**Order**	**Pollinator host-switch events (from - to)**	***D*-statistics**	**BUCKy**	**PhyloNetworks**	**fig_A–CP**	**fig_P–CP**	**fig_A–MT**	**fig_P–MT**	**CP–MT**	**fig_A–wasp_C**	**fig_P–wasp_C**	**fig_A–wasp_M**	**fig_P–wasp_M**	**CP–wasp_C**	**CP–wasp_M**	**MT–wasp_C**	**MT–wasp_M**
**1**	Subg. *Sycomorus* to subg. *Sycidium*	**Yes**	No	No	No	No	**Yes**	No	**Yes**	**Yes**	**Yes**	**Yes**	**Yes**	**Yes**	**Yes**	**Yes**	**Yes**
**2**	Sect. *Eriosycea* to sect. *Pharmacosycea*	**Yes**	No	No	No	No	No	No	No	**Yes**	No	No	No	No	No	**Yes**	**Yes**
**3**	Sect. *Eriosycea* to *F. carica* clade	**Yes**	**Yes**	No	No	No	**Yes**	**Yes**	No	**Yes**	**Yes**	**Yes**	No	No	No	No	No
**4**	GTS clade to ancestor of sect. *Americana* and *Galoglychia*	**Yes**	**Yes**	**Yes**	No	No	No	No	No	**Yes**	No	No	No	No	No	No	No
**5**	Ancestor of sect. *Americana* and *Galoglychia* to sect. *Urostigma*	**Yes**	**Yes**	No	No	No	No	No	No	**Yes**	**Yes**	No	**Yes**	No	**Yes**	**Yes**	**Yes**
**6**	Sect. *Americana* to sect. *Conosycea*	**Yes**	No	No	No	No	No	No	**Yes**	**Yes**	No	No	No	**Yes**	**Yes**	No	No
**7**	Sect. *Ficus* to subg. *Synoecia*	**Yes**	No	**Yes** [a]	No	No	No	No	**Yes**	**Yes**	No	No	No	**Yes**	**Yes**	**Yes**	No
**8**	*F. carica* clade to sect. *Oreosycea*	**Yes**	No	**Yes** [a]	No	**Yes**	No	No	No	**Yes**	**Yes**	**Yes**	**Yes**	**Yes**	**Yes**	**Yes**	**Yes**

NB: Yes or No: indicates whether each hybridization relationship was supported (Yes) or not (No) by the method in question. a: indicates that the hybridization detected involved an ancient clade of one group. fig_A and fig_P: indicate that the Astral tree or the primary concordance tree, respectively, was used to represent *Ficus* nuclear

phylogeny, respectively. CP and MT: indicate *Ficus* phylogenies based on chloroplast and on mitochondrial genomes, respectively. fig_C and fig_M: indicate pollinator phylogenies extracted from Cruaud *et al.*[12] and Machado *et al.*[14], respectively.

Supplementary Table 8. Summary of evidence for hybridization events among main clades of *Ficus*. There are three columns of direct evidence for hybridization detection, and 13 columns of indirect evidence based on cophylogenetic reconciliation using JANE, when associate switches were inferred to have occurred between related figs groups.

	**Direct evidence**			**Indirect evidence**												
**Hybridization events**	**D-statistics**	**BUCKy**	**PhyloNetworks**	**fig_A–CP**	**fig_P–CP**	**fig_A–MT**	**fig_P–MT**	**CP–MT**	**fig_A–wasp_C**	**fig_P–wasp_C**	**fig_A–wasp_M**	**fig_P–wasp_M**	**CP–wasp_C**	**CP–wasp_M**	**MT–wasp_C**	**MT–wasp_M**
Subg. *Urostigma* - Sect. *Oreosycea*	**Yes**	**Yes**	No	No	No	No	**Yes**	No	No	No	No	**Yes**	**Yes**	No	**Yes**	**Yes**
Subg. *Urostigma* - Sect. *Pharmacosycea*	**Yes**	No	No	No	No	**Yes**	No	**Yes**	No	No	No	No	**Yes**	No	No	No
Subg. *Urostigma* - Gynodioecious	**Yes**	**Yes**	**Yes**	**Yes**	**Yes**	**Yes**	**Yes**	**Yes**	**Yes**	**Yes**	**Yes**	**Yes**	**Yes**	No	No	No
Subg. *Urostigma* - CC clade	**Yes**	**Yes** [a]	No	No	No	No	**Yes**	No	**Yes**	**Yes**	**Yes**	No	No	No	No	No
Subg. *Urostigma* - GTS clade	**Yes**	**Yes**	**Yes**	No	No	No	**Yes**	**Yes**	No	No	No	No	**Yes**	No	No	No
Subg. *Urostigma* - Subg. *Sycomorus*	**Yes**	**Yes** [a]	No	**Yes**	**Yes**	**Yes**	**Yes**	**Yes**	No	No	No	No	No	No	No	No
Sect. Oreosycea - Sect. *Pharmacosycea*	**Yes**	No	No	No	No	No	No	No	No	No	No	**Yes**	No	No	No	No
Sect. Oreosycea - Gynodioecious	**Yes**	**Yes**	**Yes**	**Yes**	**Yes**	**Yes**	**Yes**	**Yes**	**Yes**	**Yes**	**Yes**	**Yes**	**Yes**	**Yes**	**Yes**	**Yes**
Sect. Oreosycea - CC clade	**Yes**	No	**Yes** [a]	No	No	No	No	No	**Yes**	**Yes**	**Yes**	**Yes**	**Yes**	**Yes**	**Yes**	**Yes**
Sect. Oreosycea - GTS clade	**Yes**	**Yes**	**Yes** [a]	No	No	No	No	No	No	No	No	**Yes**	No	No	No	No
Sect. Oreosycea - Subg. *Sycomorus*	**Yes**	No	**Yes** [a]	**Yes**	**Yes**	**Yes**	**Yes**	**Yes**	No	No	No	No	No	No	No	No
Sect. *Pharmacosycea* - Gynodioecious	**Yes**	No	No	**Yes**	No	No	No	No	**Yes**	**Yes**	**Yes**	**Yes**	No	No	**Yes**	**Yes**
Sect. *Pharmacosycea* - CC clade	**Yes**	No	No	No	No	No	No	No	No	**Yes**	**Yes**	No	No	No	No	No
Sect. *Pharmacosycea* - GTS clade	**Yes**	No	No	No	No	No	No	No	**Yes**	No	No	**Yes**	No	No	**Yes**	**Yes**
Sect. *Pharmacosycea* - Subg. *Sycomorus*	**Yes**	No	No	No	No	No	No	No	No	No	No	No	No	No	No	No

CC clade - GTS clade	**Yes**	**Yes**	**Yes** [a]	**Yes**	No	**Yes**	**Yes**	**Yes**	**Yes**	**Yes**	**Yes**	**Yes**	**Yes**	No	No	No
CC clade - Subg. *Sycomorus*	**Yes**	**Yes**	**Yes** [a]	No	No	**Yes**	No	**Yes**	**Yes**	**Yes**	**Yes**	**Yes**	**Yes**	**Yes**	**Yes**	**Yes**
GTS clade - Subg. *Sycomorus*	**Yes**	No	**Yes** [a]	**Yes**	**Yes**	**Yes**	No	**Yes**	No	No	**Yes**	**Yes**	No	**Yes**	No	**Yes**
Within Subg. *Urostigma*	**Yes**	**Yes**	No	**Yes**	**Yes**	No	**Yes**	**Yes**	**Yes**	**Yes**	**Yes**	**Yes**	**Yes**	**Yes**	**Yes**	**Yes**
Within Gynodioecious	**Yes**	**Yes**	**Yes**	**Yes**	**Yes**	**Yes**	**Yes**	**Yes**	**Yes**	**Yes**	**Yes**	**Yes**	**Yes**	**Yes**	**Yes**	**Yes**
Within GTS clade	**Yes**	**Yes**	No	No	No	**Yes**	No	**Yes**	**Yes**	No	**Yes**	**Yes**	**Yes**	**Yes**	**Yes**	No
Within Subg. *Sycomorus*	**Yes**	No	**Yes**	No	No	No	No	**Yes**	No	**Yes**	No	**Yes**	**Yes**	**Yes**	**Yes**	**Yes**

NB: Yes or No: indicates whether each hybridization relationship was supported (Yes) or not (No) by the method in question. a: indicates that the hybridization detected involved an ancient clade of one group. fig_A and fig_P: indicate that the Astral tree or the primary concordance tree was used to represent *Ficus* nuclear phylogeny, respectively. CP and MT: indicate *Ficus* phylogenies based on chloroplast and on mitochondrial genomes, respectively. fig_C and fig_M: indicate pollinator phylogenies extracted from Cruaud *et al.*[12] and Machado *et al.*[14], respectively.

Supplementary Table 9: **Genome ploidy estimated with nQuire program[17].** All species are inferred as diploid species. Code as: create -b bam -o prefix -f 0.3 && nQuire lrdmodel bin.

species	free	dip	tri	tet	d_dip	d_tri	d_tet	ploid_level	depth
Antiaris toxicaria	136694	113333	94911	62607	23361	41783	74087	**2**	6.15
F. adhatodifolia	9674364	7813267	7049547	4588406	1861097	2624817	5085958	**2**	14.76
F. aurea	12974090	11132222	8546683	5621556	1841868	4427407	7352534	**2**	25.75
F. carica	12400885	10507946	8481016	5600413	1892938	3919869	6800472	**2**	16.87
F. cyathistipula	5765837	4327204	4412042	2798811	1438633	1353795	2967026	**2**	16.21
F. cyrtophylla	11813156	9741080	8256229	5249115	2072077	3556928	6564042	**2**	20.88
F. esquiroliana	12403296	10992529	7648503	5333636	1410767	4754793	7069660	**2**	25.89
F. gasparriniana	13276642	10957307	9256981	5917221	2319335	4019660	7359421	**2**	20.94
F. hainanensis	2657478	2215515	1861465	1230483	441963	796013	1426994	**2**	11.79
F. hispida	9599077	7471410	6655309	3801578	2127667	2943768	5797499	**2**	43.35
F. maxima	6163743	5791497	3170564	2765490	372246	2993179	3398253	**2**	72.58
F. microcarpa	13020529	10633252	9290632	5884873	2387277	3729897	7135656	**2**	18.42
F. microdjctya	9727627	8102500	6713819	4286094	1625127	3013808	5441533	**2**	23.34
F. religiosa	11705431	9876450	7880790	5075164	1828982	3824642	6630267	**2**	25.22
F. sagittata	12081420	10166284	8096876	5174424	1915136	3984544	6906996	**2**	23.38
F. vasculosa	3996988	3172295	2940002	1867299	824692	1056986	2129689	**2**	18.49
Morus alba	2215757	1705548	1604804	941238	510209	610953	1274519	**2**	20.81

Supplementary Table 10: Model selection results for reconstruction of ancestral distributions with R package "BioGeoBEARS". Best model based on Astral tree and primary concordance tree were indicated with bold word.

Model	LnL	numparams	AIC	AIC_wt
		**Astral tree**		
DEC	-25.74	2	55.47	0.023
DEC+J	-21.8	3	49.6	0.44
DIVALIKE	-24.55	2	53.1	0.076
**DIVALIKE+J**	**-22.17**	**3**	**50.33**	**0.31**
BAYAREALIKE	-29.96	2	63.91	0.0003
BAYAREALIKE+J	-22.85	3	51.7	0.15
		**Primary concordance tree**		
DEC	-27.84	2	59.68	0.002
DEC+J	-21.74	3	49.47	0.261
DIVALIKE	-24.98	2	53.96	0.028
**DIVALIKE+J**	**-20.90**	**3**	**47.79**	**0.605**
BAYAREALIKE	-30.37	2	64.74	0.000
BAYAREALIKE+J	-22.66	3	51.31	0.104

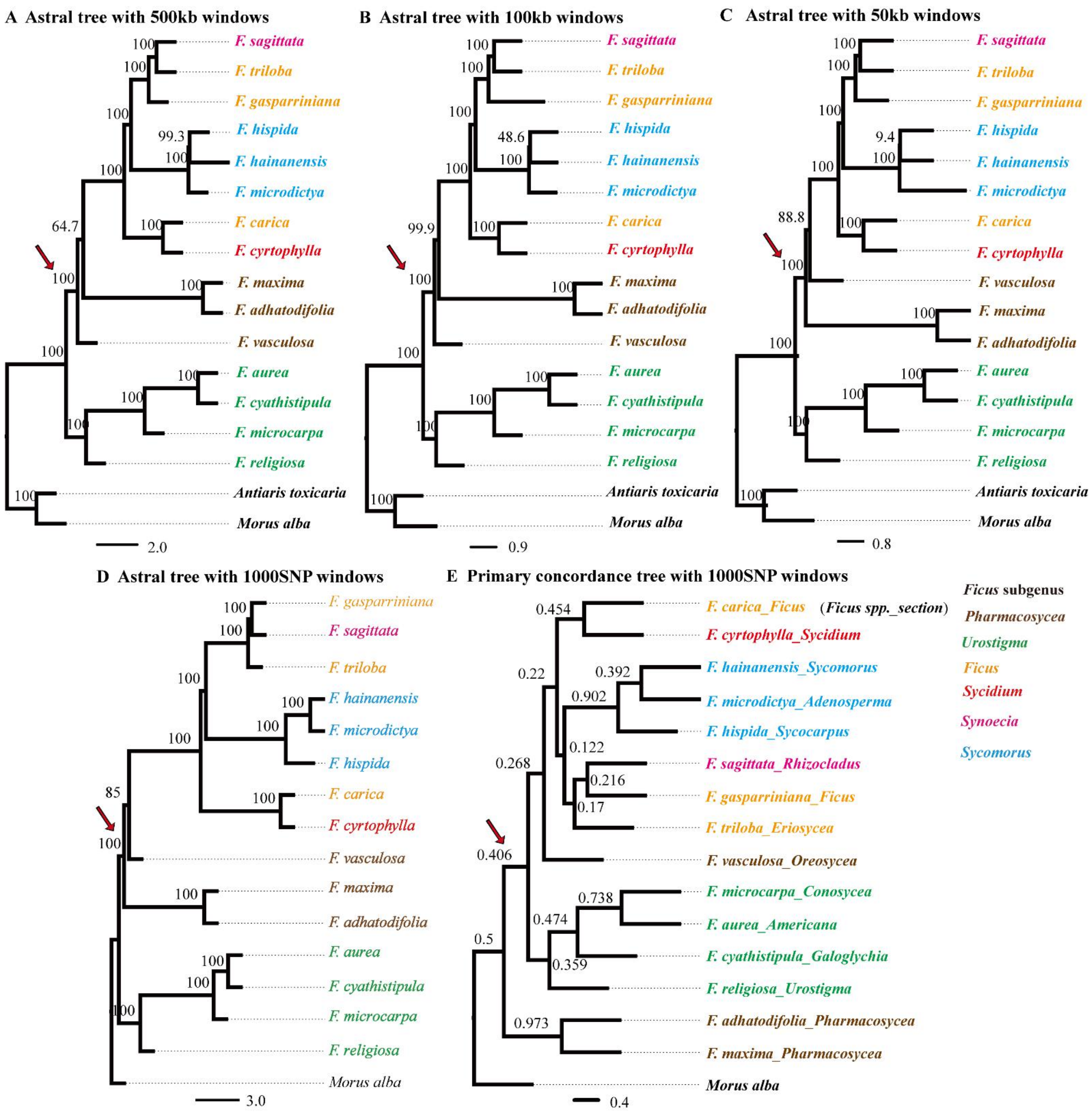

Supplementary Fig. 1: *Ficus* species trees estimated based on nuclear genomic datasets. (A-D) Four Astral species trees with bootstrap values at the nodes based on nuclear genomic-windows datasets with different windows lengths, inferred with ASTRAL II. (E) Primary concordance tree (PCT) with concordance factor values at the nodes based on the Windows1000SNPs dataset, inferred with BUCKy. For information on windows datasets, refer to Supplementary Table 3. *Ficus* species and section names are given in the *Ficus* phylogeny. *Ficus* species belonging to the same subgenus are marked with the same color. Nodes related to the potential basal clades of *Ficus* are marked with a red arrow in each tree. Support values are substantially higher in the four Astral trees, which support subgenus *Urostigma* (green) as the basal clade of *Ficus*, while values of concordance factor are lower in the PCT, which supports section *Pharmacosycea* (brown) as the basal clade of *Ficus*. Other potential basal clades are shown in Supplementary Table 4. For details of results, refer to Source Data Appendices 1 and 2.

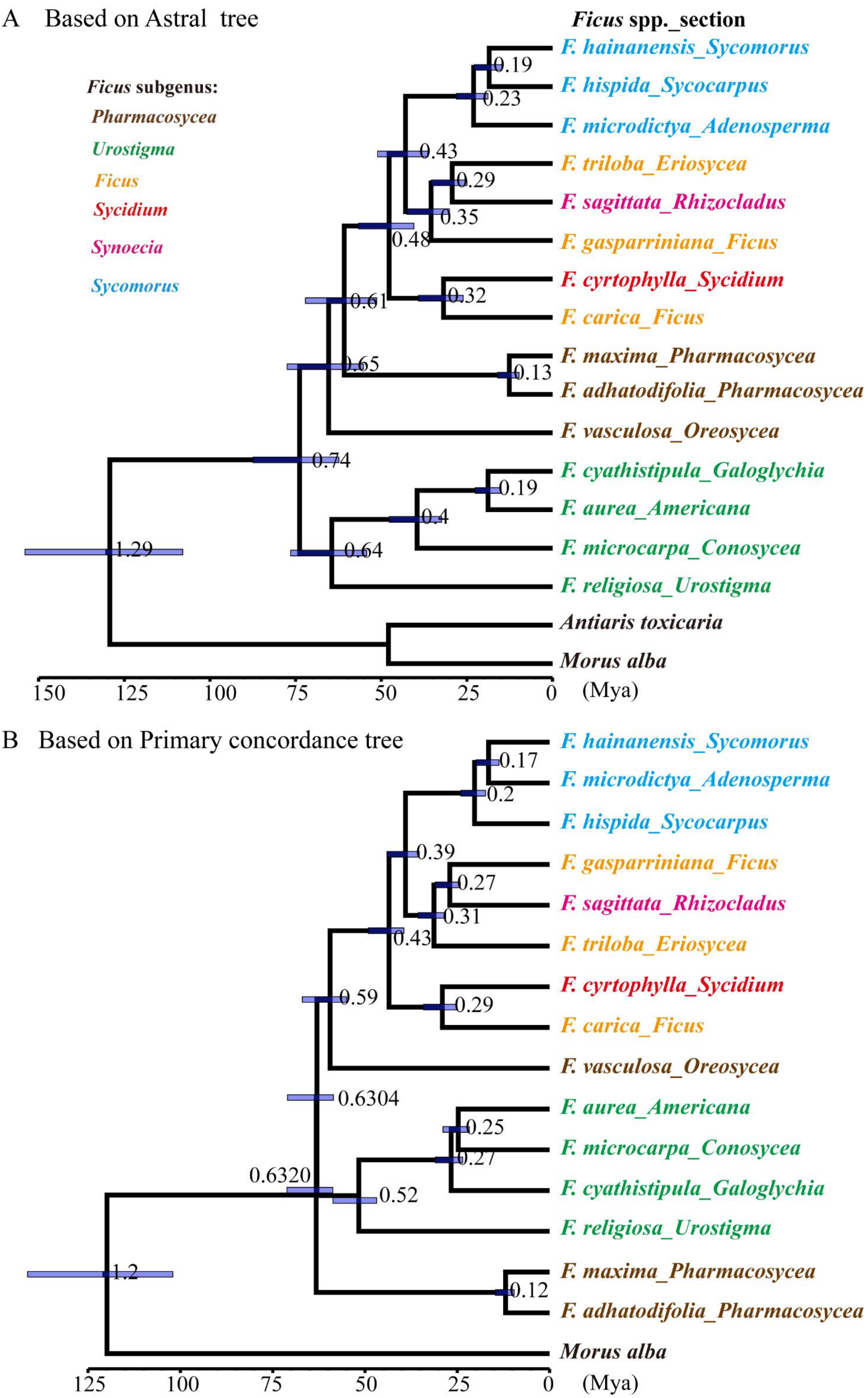

A Based on Astral tree
Ficus spp._section
Ficus subgenus:
Pharmacosycea
Urostigma
Ficus
Sycidium
Synoecia
Sycomorus
F. hainanensis_Sycomorus
F. hispida_Sycocarpus
F. microdictya_Adenosperma
F. triloba_Eriosycea
F. sagittata_Rhizocladus
F. gasparriniana_Ficus
F. cyrtophylla_Sycidium
F. carica_Ficus
F. maxima_Pharmacosycea
F. adhatodifolia_Pharmacosycea
F. vasculosa_Oreosycea
F. cyathistipula_Galoglychia
F. aurea_Americana
F. microcarpa_Conosycea
F. religiosa_Urostigma
Antiaris toxicaria
Morus alba
0.19
0.23
0.43
0.29
0.35
0.48
0.32
0.61
0.65
0.13
0.74
0.19
0.4
0.64
1.29
150 125 100 75 50 25 0 (Mya)
B Based on Primary concordance tree
F. hainanensis_Sycomorus
F. microdictya_Adenosperma
F. hispida_Sycocarpus
F. gasparriniana_Ficus
F. sagittata_Rhizocladus
F. triloba_Eriosycea
F. cyrtophylla_Sycidium
F. carica_Ficus
F. vasculosa_Oreosycea
F. aurea_Americana
F. microcarpa_Conosycea
F. cyathistipula_Galoglychia
F. religiosa_Urostigma
F. maxima_Pharmacosycea
F. adhatodifolia_Pharmacosycea
Morus alba
0.17
0.2
0.39
0.27
0.31
0.43
0.29
0.59
0.6304
0.25
0.27
0.6320
0.52
1.2
0.12
125 100 75 50 25 0 (Mya)

Supplementary Fig. 2: Divergence times in *Ficus* inferred with MCMCTree using Astral tree (A) and primary concordance tree (B) based on nuclear genomic datasets. Values and bars at the nodes indicate the mean and 95% credibility interval of node age. *Ficus* species and section names are given in the *Ficus* phylogeny. *Ficus* species belonging to the same subgenus are marked with the same color. Refer to Appendix 3 for detail results.

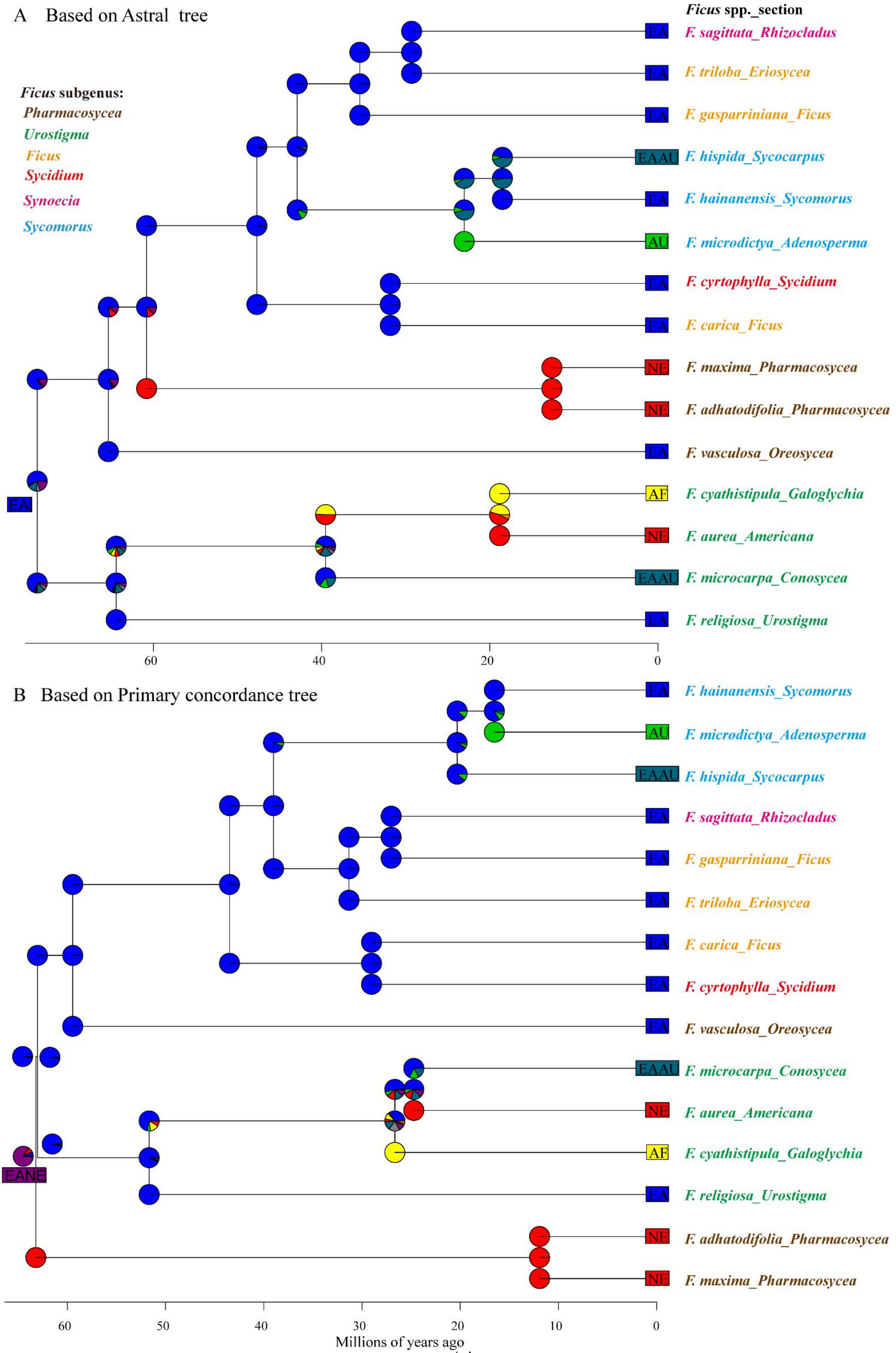

A Based on Astral tree
Ficus spp._section
Ficus subgenus:
Pharmacosycea
Urostigma
Ficus
Sycidium
Synoecia
Sycomorus
EA
F. sagittata_Rhizocladus
F. triloba_Eriosycea
F. gasparriniana_Ficus
EAAU
F. hispida_Sycocarpus
F. hainanensis_Sycomorus
AU
F. microdictya_Adenosperma
F. cyrtophylla_Sycidium
F. carica_Ficus
NE
F. maxima_Pharmacosycea
F. adhatodifolia_Pharmacosycea
F. vasculosa_Oreosycea
AF
F. cyathistipula_Galoglychia
F. aurea_Americana
F. microcarpa_Conosycea
F. religiosa_Urostigma
60
40
20
0
B Based on Primary concordance tree
EANE
60
50
40
30
20
10
0
Millions of years ago

Supplementary Fig. 3: Estimation of ancestral distribution of *Ficus* following Astral tree (A) and primary concordance tree (B) under best models with BioGeoBEARS R package. Pie charts show the relative probabilities of possible geographic distributions, with the most probable ancestral distribution labeled beside. Current species distribution (tip symbols) and he relative probabilities of possible geographic ranges (pie charts) of all nodes are indicated. Ranges that are combinations of these four distributions are shown by colors formed by mixing of the component colors. *Ficus* species and section names are given in the *Ficus* phylogeny. *Ficus* species belonging to the same subgenus are marked with the same color. Species distributions and taxonomic classification follow Cruaud et al. (2012). A) based on Astral tree, *Ficus* is inferred originated in Eurasia in ~74 Mya, and all main clades coexisted for more than 10 million years in Eurasia, before dispersal to other areas. The long coexistence history provided opportunities for ancient hybridization between different clades. B) based on primary concordance tree, *Ficus* is inferred originated in either Eurasia or Neotropics (combing of Eurasia and Neotropics) in ~63.2 Mya, followed with a very quick divergence between Neotropical section Pharmacosycea and rest figs originated in Eurasia. However, the later pattern does not fit the paleogeographic pattern around that time, when two pale-continents (Eurasia and Neotropics) were separately (http://scotese.com/K/t.htm). For details of the results, refer to methods and Appendix 4 for details.

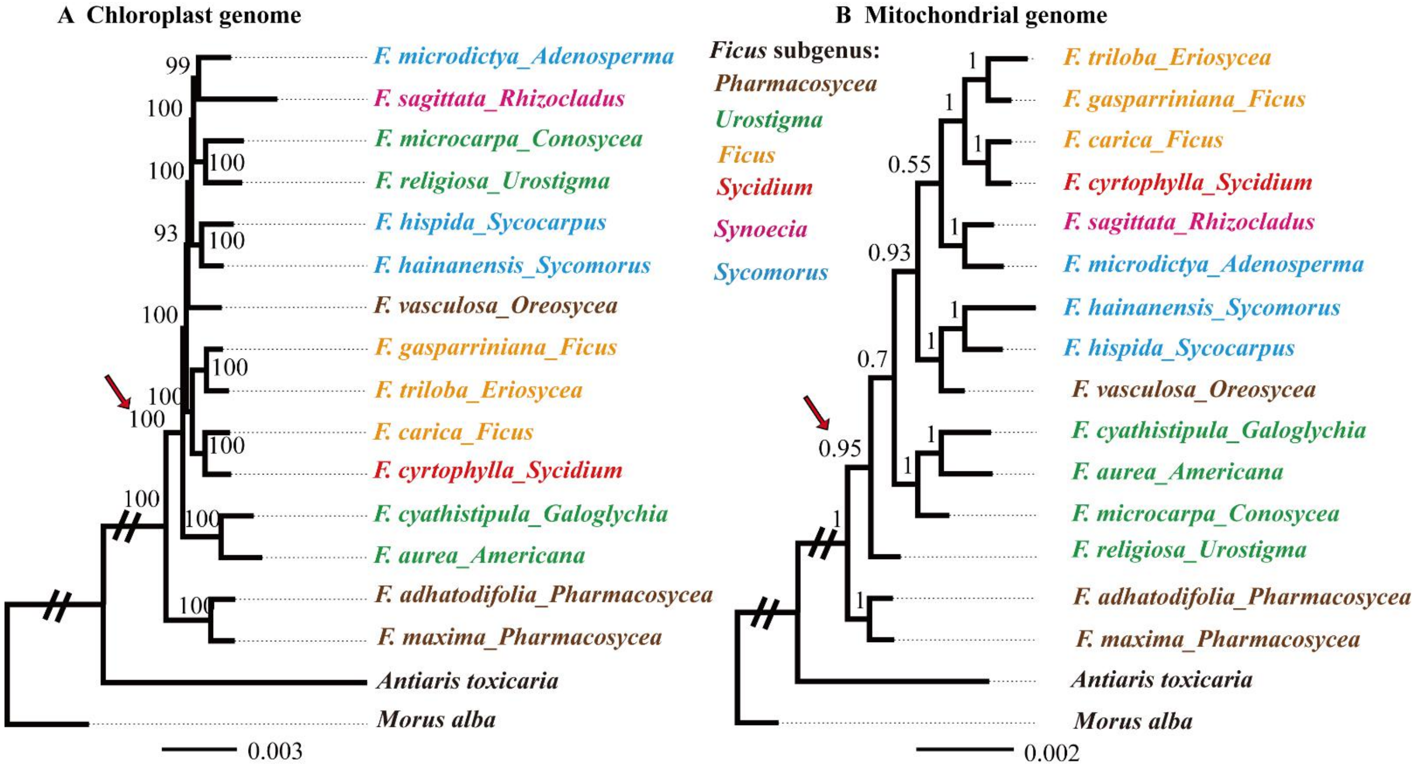

Supplementary Fig. 4: ML trees based on *Ficus* chloroplast and mitochondrial genomes. Phylogenies were inferred based on the concatenated whole chloroplast or mitochondrial genomic sequence using RAxML (A) and FastTree (B), respectively. Node values indicate the bootstrap values (A) or local support values (B). *Ficus* species and section names are given in the *Ficus* phylogeny. *Ficus* species belonging to the same subgenus are marked with the same color. Both organelle phylogenies support section *Pharmacosycea* as being sister to all other figs. This position is the same as that in the PCT tree based on nuclear genomes, but different from that in the Astral trees. For details of the results, refer to Appendix 5.

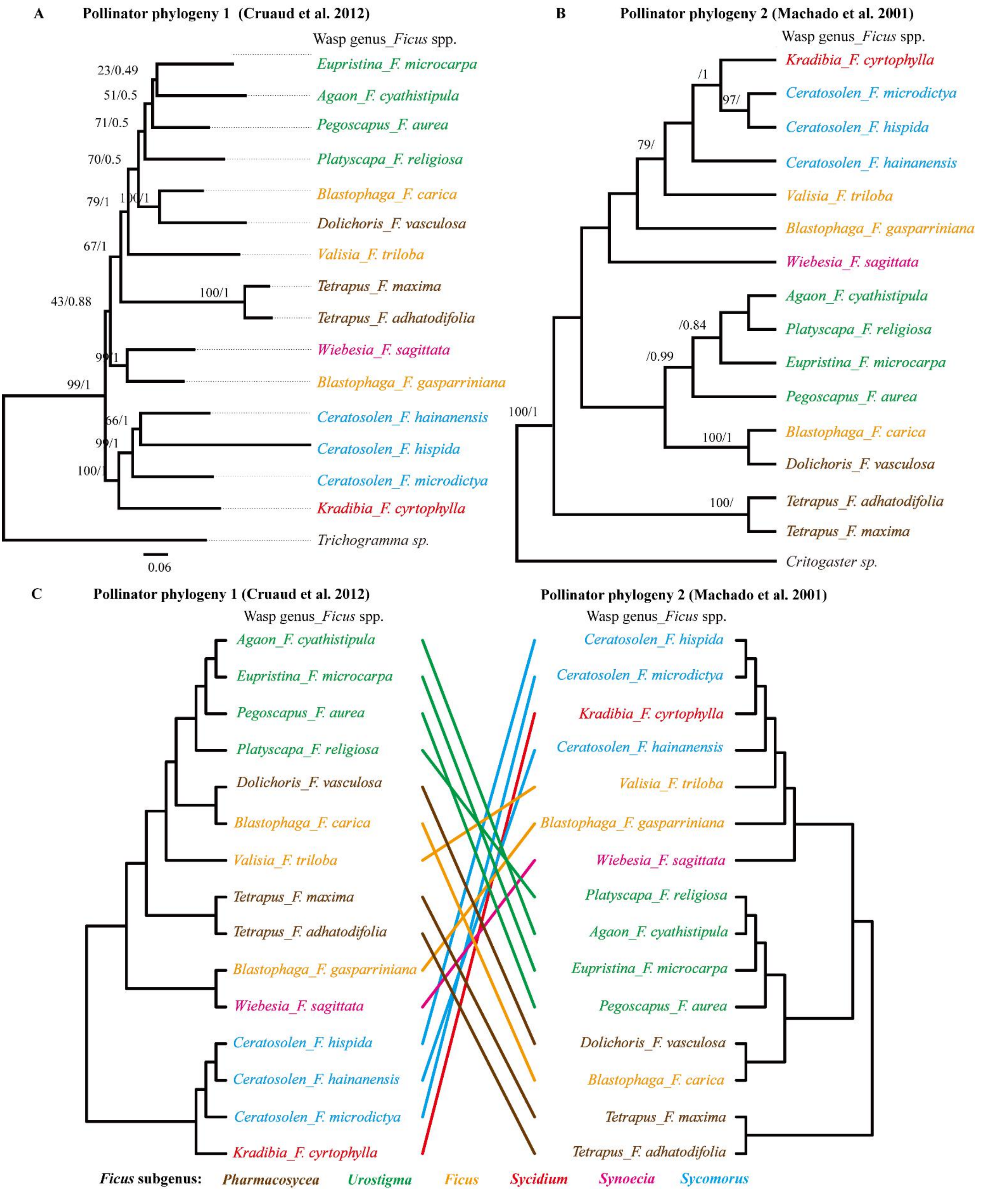

Supplementary Fig. 5: Phylogenies of pollinating fig wasps associated with the studied fig species used for co-phylogenetic analysis in JANE. A) Phylogeny 1 was extracted from the largest published wasp phylogeny, based on six genes sequenced in 200 wasp species[12]. B) Phylogeny 2 represents an alternative view of fig wasp evolution based on the mitochondrial COI gene[14], and is supported by several other studies[16,17]. Names of wasp genera and associated host figs are shown in the tip labels. Bootstrap percentages/posterior probabilities are shown above nodes when available. C) Face-to-face comparison of the two wasp phylogenies, showing the extensive differences in the relationships they infer. Host *Ficus* species belonging to the same subgenus are marked with the same color. See Supplementary Table 6 (last line) for details on the quantitative estimation of differences.

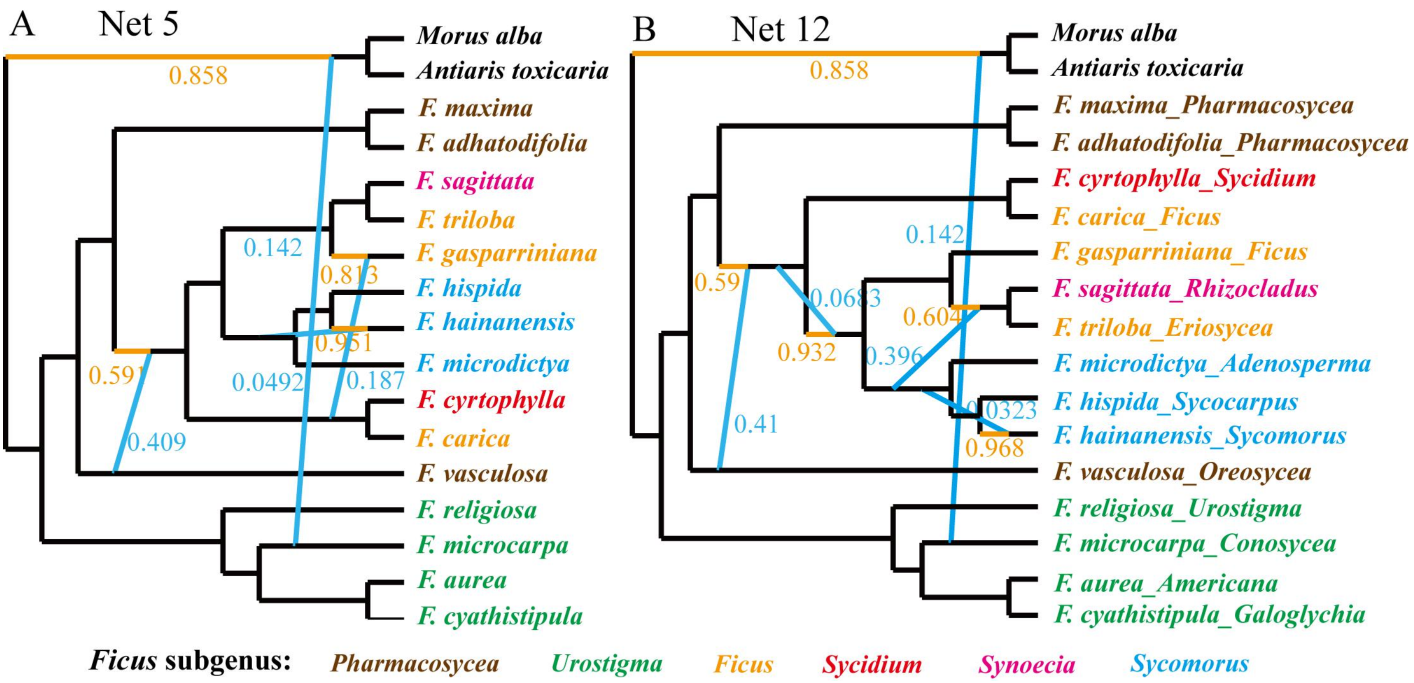

Supplementary Fig. 6. *Ficus* phylogenetic networks inferred by PhyloNetworks based on 500kb windows datasets. Phylogenetic networks under different maximum hybrid node numbers (hmax) are shown, representing hybridization relationships under hmax set to 5 and 12. Colored lines represent the inferred hybridization events. The colored numbers on these lines indicate the genomic ratio from the two parental clades (blue for the minor clade, orange for the major clade), with the branch representing the major clade also being marked with orange. Five events involving deeper nodes within *Ficus* were detected based on a dataset with shorter and size-fixed window, the 500-kb windows (500 kb per window). *Ficus* species and section names are given in the *Ficus* phylogeny. *Ficus* species belonging to the same subgenus are marked with the same color. Notably, one hybridization event is inferred to have occurred between *F. microcarpa* and the non-*Ficus* outgroups. This suggests that hybridization involved potential extinct taxa near the base of *Ficus* phylogeny, or that descendants of the parental taxa are missing from the sample.

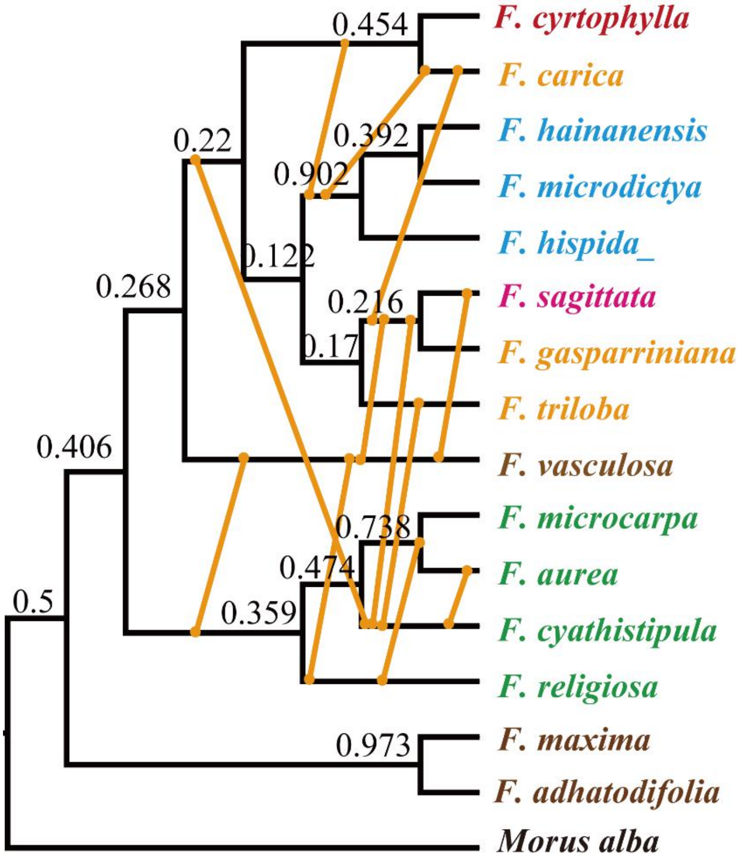

Supplementary Fig. 7. Hybridization events within *Ficus* inferred with BUCKy. The primary concordance tree for *Ficus* (with concordance factors indicated at the nodes), with branches indicated by horizontal lines and hybridization events indicated with vertical lines, as inferred in BUCKy based on the 1000SNPs windows dataset. For all pairs of species or clades with a dot where a vertical line crosses the (horizontal) branch indicating the species or clade, hybridization was detected involving taxa connected by the same vertical line. *Ficus* classification and current distribution at the section level are also shown. *Ficus* species belonging to the same subgenus are marked with the same color. Refer to Appendix 2 for details.

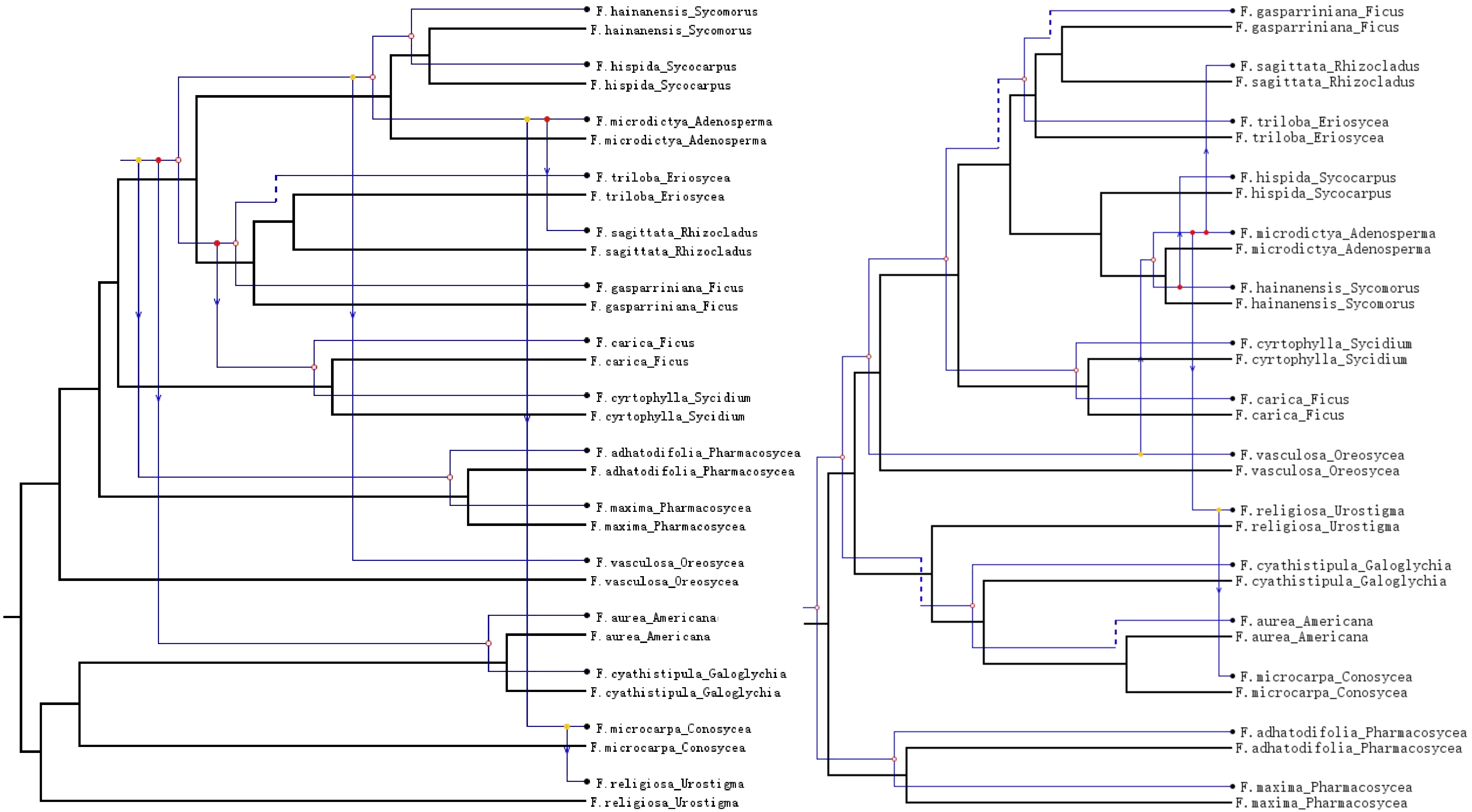

Supplementary Fig. 8: One of the equally most parsimonious reconciliations between *Ficus* nuclear (Left: ASTRAL tree; Right: Primary concordance tree) and chloroplast phylogenies inferred with JANE. The black tree represents the nuclear genomic phylogeny and the blue tree the chloroplast genome phylogeny. Hollow red circles on nodes represent codivergence events; filled colored dots and arrows on the blue lines represent events of associate host switch followed duplication, while filled orange dots indicate the existence of another potential switch location with equal cost, and a filled red dot means that all other potential switch locations have higher cost. For the optimal cost settings used, refer to Supplementary Table 6.

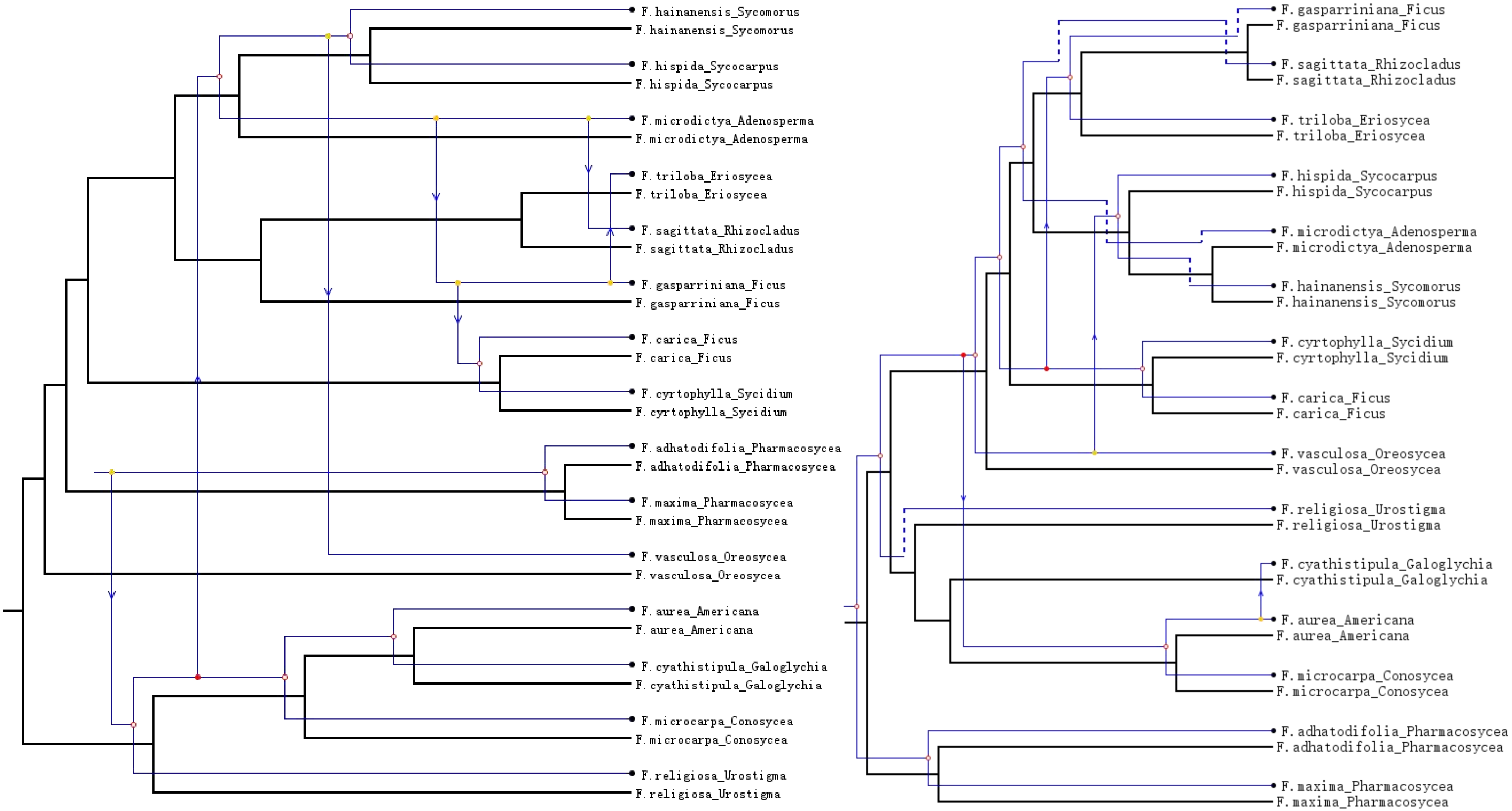

Supplementary Fig. 9: One of the equally most parsimonious reconciliations between *Ficus* nuclear (Left: ASTRAL tree; Right: Primary concordance tree) and mitochondrial phylogenies inferred with JANE. The black tree represents the nuclear genomic phylogeny ("host") and the blue tree the mitochondrial genome phylogeny. Hollow red circles on nodes represent codivergence events; filled colored dots and arrows on the blue lines represent events of associate host switch following duplication, while filled orange dots indicate the existence of another potential switch location with equal cost, and a filled red dot means that all other potential switch locations have higher cost. For the optimal cost settings used, refer to Supplementary Table 6.

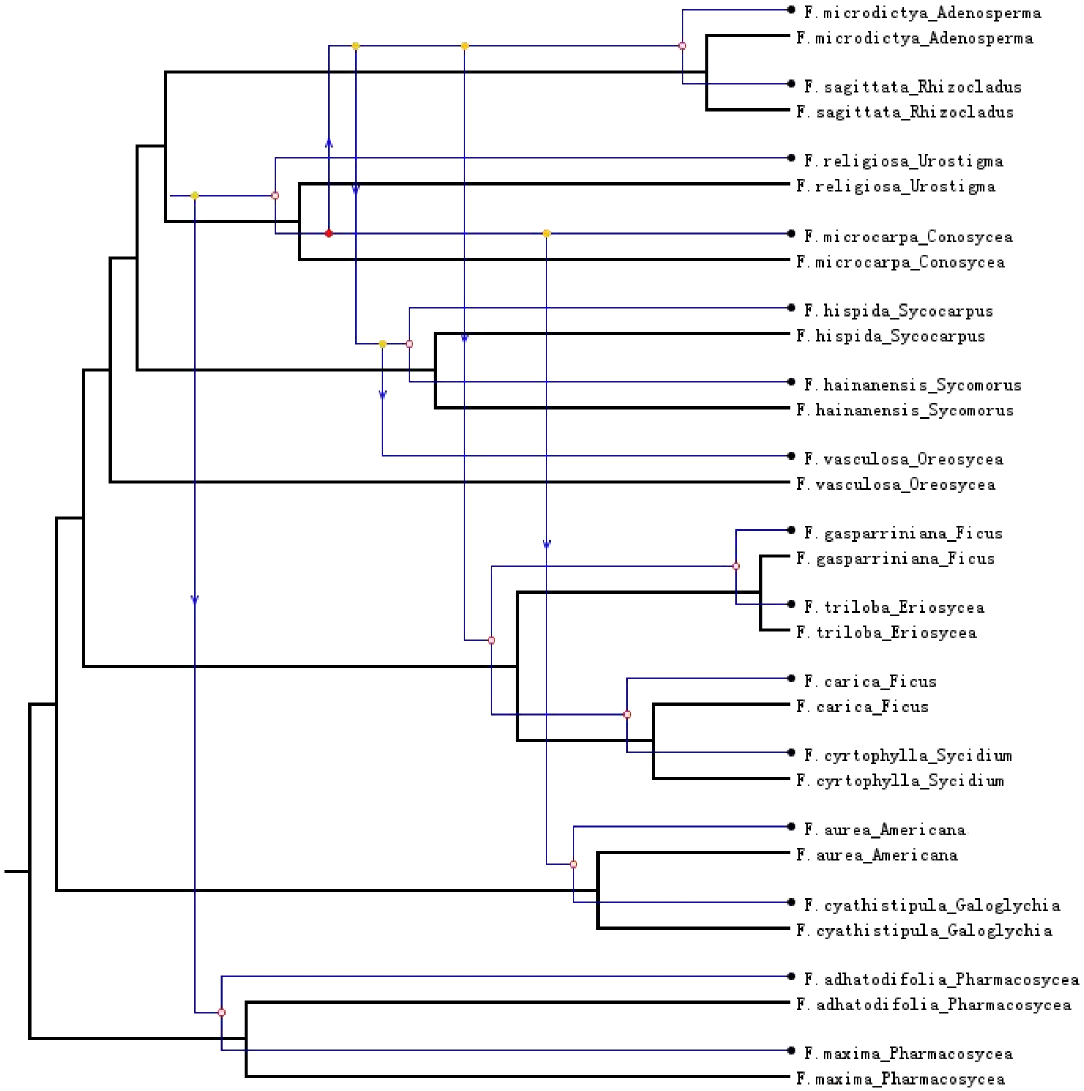

Supplementary Fig. 10: One of the equally most parsimonious reconciliations between *Ficus* chloroplast and mitochondrial phylogenies inferred with JANE. The black tree represents the chloroplast genomic phylogeny and the blue tree the mitochondrial genome phylogeny. Hollow red circles on nodes represent codivergence events; filled color dots and arrows on the blue lines represent events of associate host switch following duplication, while filled orange dots indicate the existence of another potential switch location with equal cost, and a filled red dot means that all other potential switch locations have higher cost. For the optimal cost settings used, refer to Supplementary Table 6.

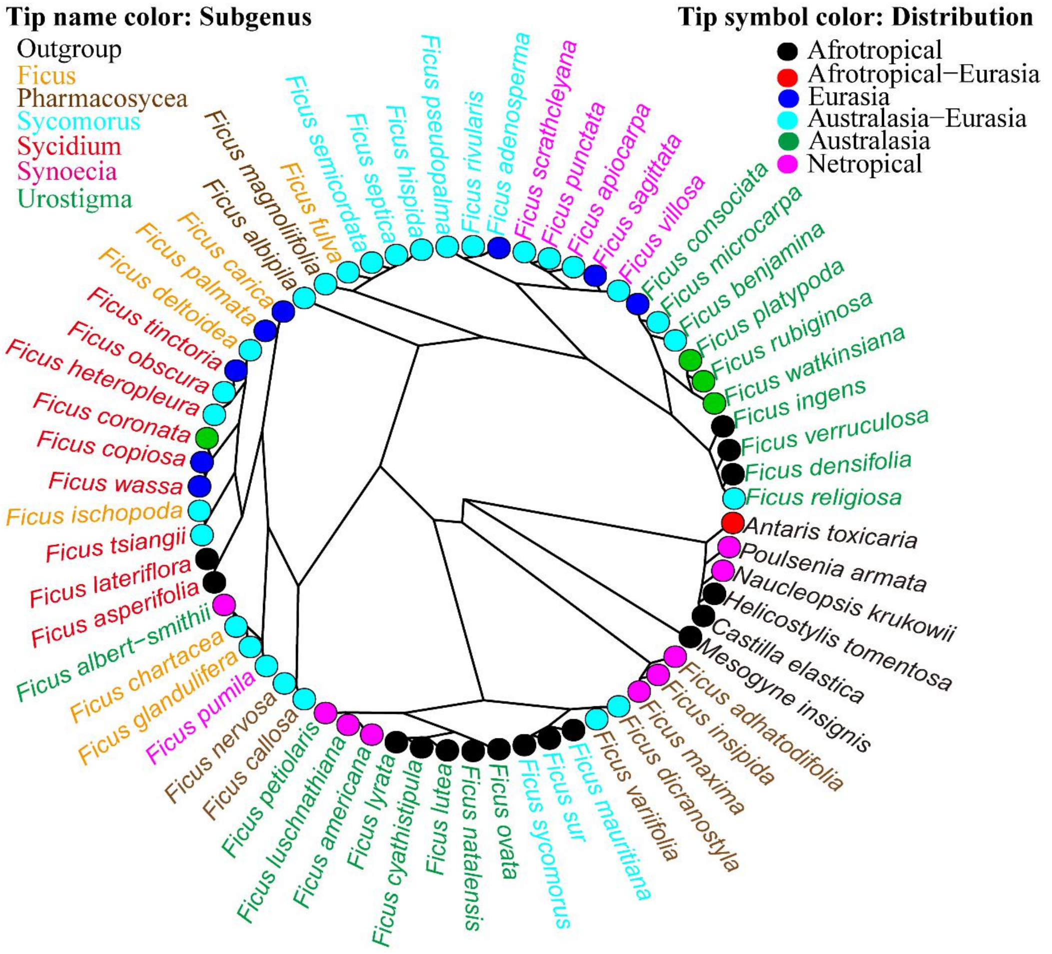

Supplementary Fig 11: Geographic clustering pattern of *Ficus* chloroplast genomes on the 59-species chloroplast phylogeny. The published 59-species chloroplast phylogeny[13] supported none of the six *Ficus* subgenera as a monophyletic clade, but revealed a pattern of geographic clustering, in which sympatric fig species from different subgenera were frequently found in the same clade. For example, specific Afrotropical (black symbol) figs of subgenera *Sycomorus* (light-blue names) and *Urostigma* (green names) clustered in the same clade, but the two subgenera are shown to be distantly related clades, and each monophyletic, in nuclear phylogenies (Fig. 1). Furthermore, gynodioecious figs from different subgenera co-distributed from Eurasia to Australasia were also repeatedly found to cluster in multiple chloroplast clades. Subgeneric classification is shown by tip name colors and geographic distribution is shown by tip symbol color.

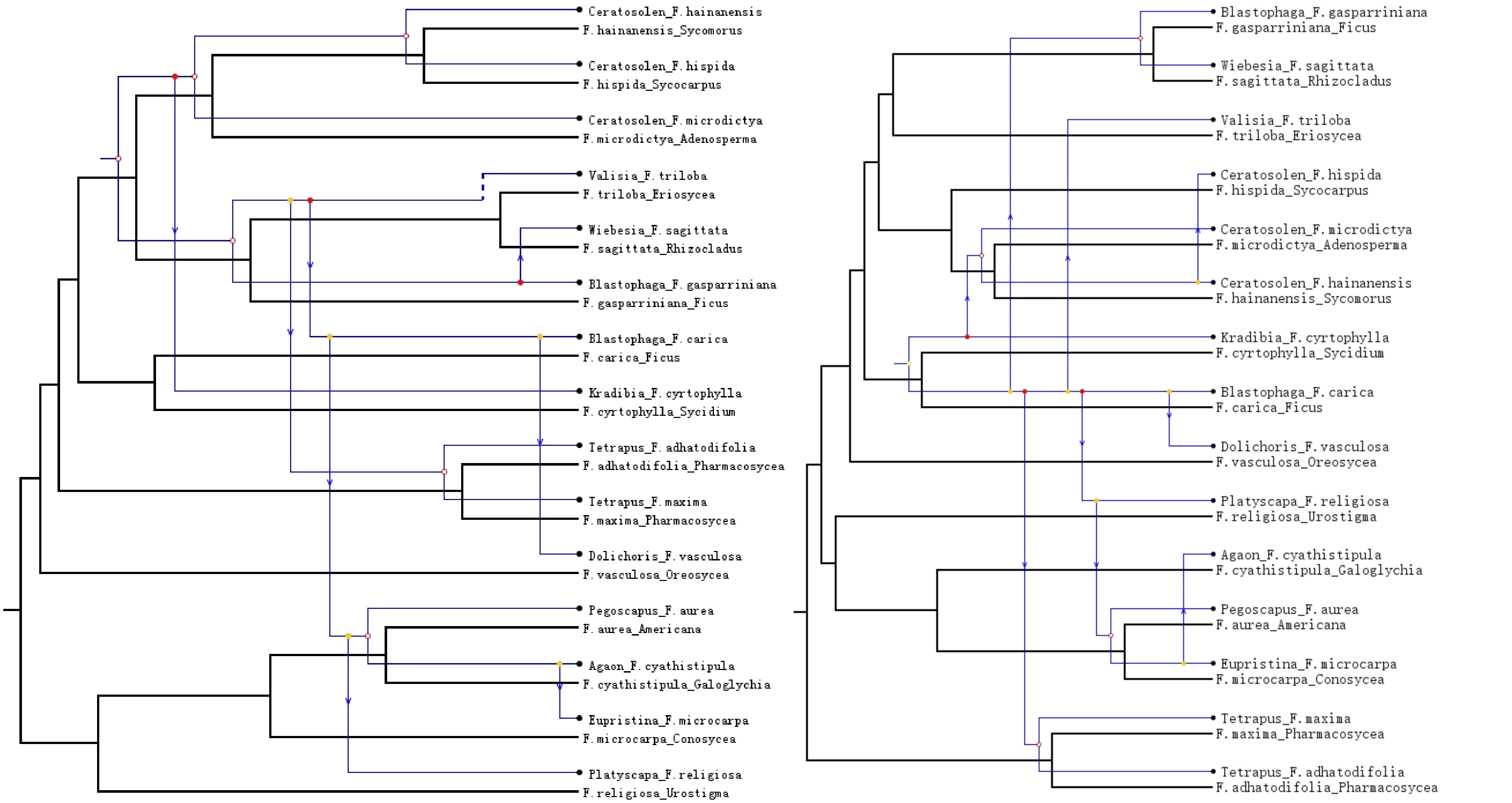

Supplementary Fig. 12: One of the equally most parsimonious reconciliations between *Ficus* nuclear phylogenies (Left: ASTRAL tree; Right: Primary concordance tree) and the pollinator phylogeny of Cruaud et al. (2012) inferred with JANE. The black tree represents the nuclear genomic phylogeny and the blue tree the pollinator phylogeny. Hollow red circles on nodes represent codivergence events; filled colored dots and arrows on the blue lines represent events of associate host switch following duplication, while filled orange dots indicate the existence of another potential switch location with equal cost, and a filled red dot means that all other potential switch locations have higher cost. For the optimal cost settings used, refer to Supplementary Table 6.

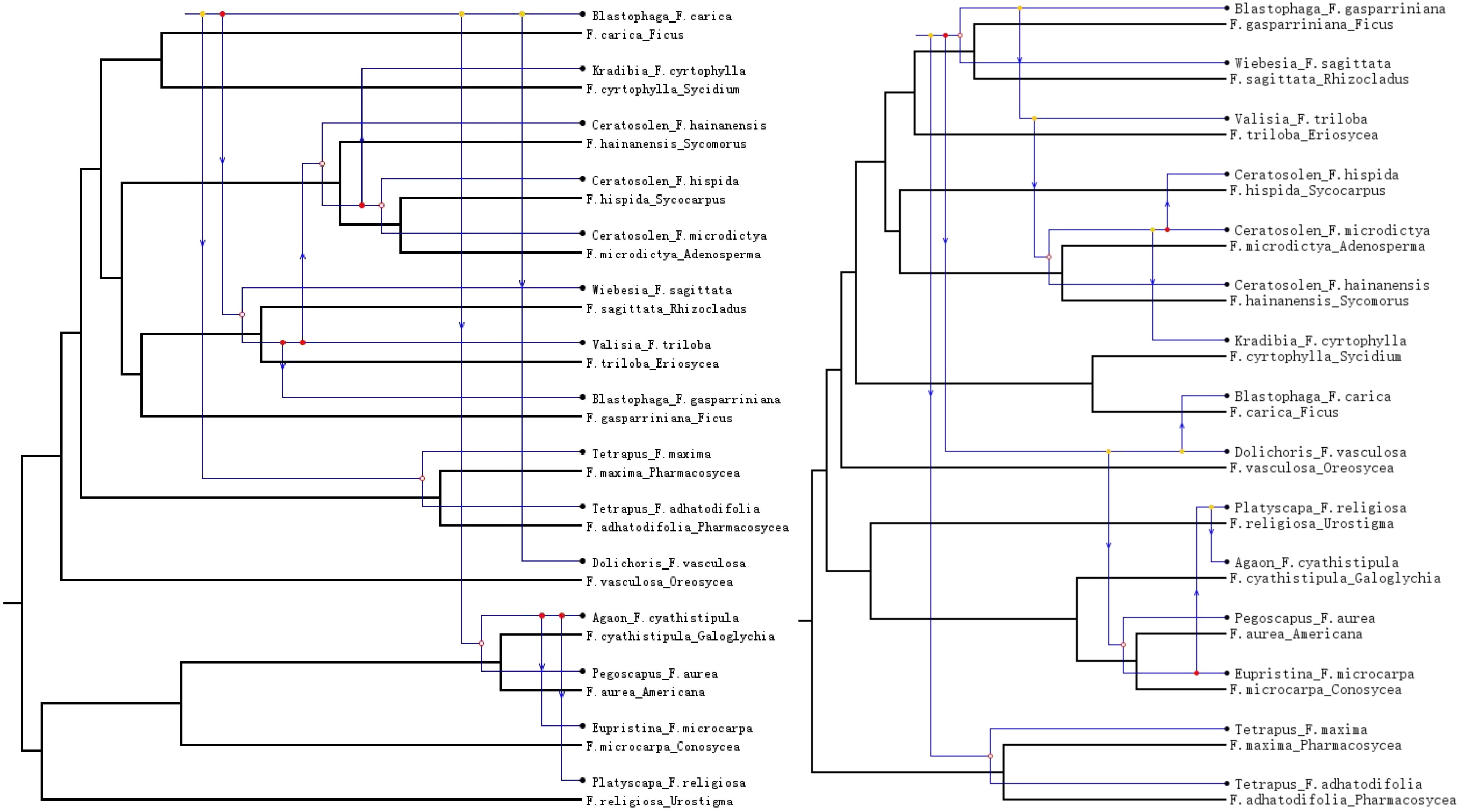

Supplementary Fig. 13: One of the equally most parsimonious reconciliations between *Ficus* nuclear phylogenies (Left: ASTRAL tree; Right: Primary concordance tree) and the pollinator phylogeny of Machado et al. (2001) inferred with JANE. The black tree represents the nuclear genomic phylogeny and the blue tree the pollinator phylogeny. Hollow red circles on nodes represent codivergence events; filled colored dots and arrows on the blue lines represent events of associate host switch following duplication, while filled orange dots indicate the existence of another potential switch location with equal cost, and a filled red dot means that all other potential switch locations have higher cost. For the optimal cost settings used, refer to Supplementary Table 6.

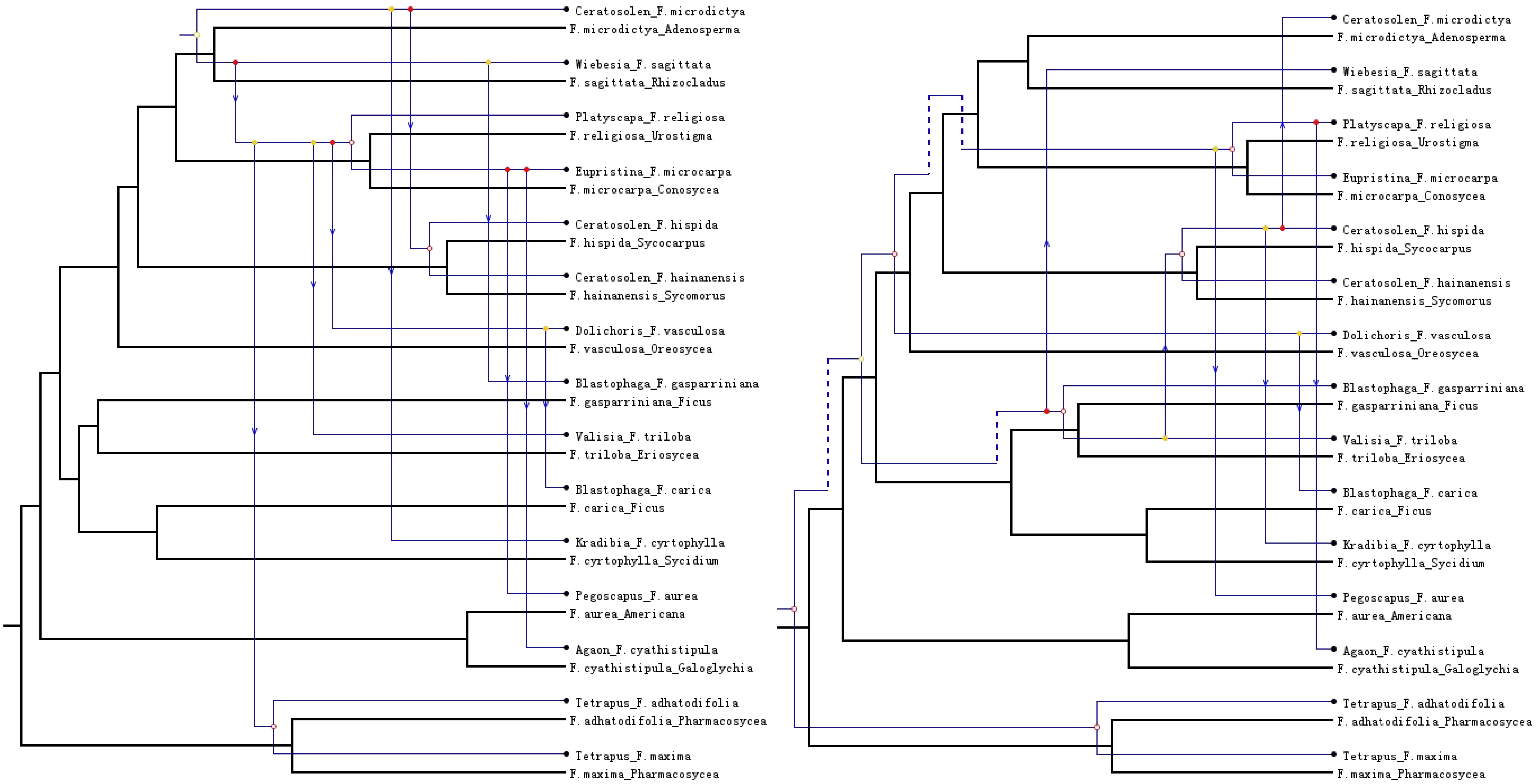

Supplementary Fig. 14: One of the equally most parsimonious reconciliations between *Ficus* chloroplast phylogeny and pollinator phylogenies (Left: Cruaud et al. phylogeny (2012); Right: Machado et al. phylogeny (2001)) inferred with JANE. The black tree represents the chloroplast genomic phylogeny and the blue tree the pollinator phylogeny. Hollow red circles on nodes represent codivergence events; filled colored dots and arrows on the blue lines represent events of associate host switch following duplication, while filled orange dots indicate the existence of another potential switch location with equal cost, and a filled red dot means that all other potential switch locations have higher cost. For the optimal cost settings used, refer to Supplementary Table 6.

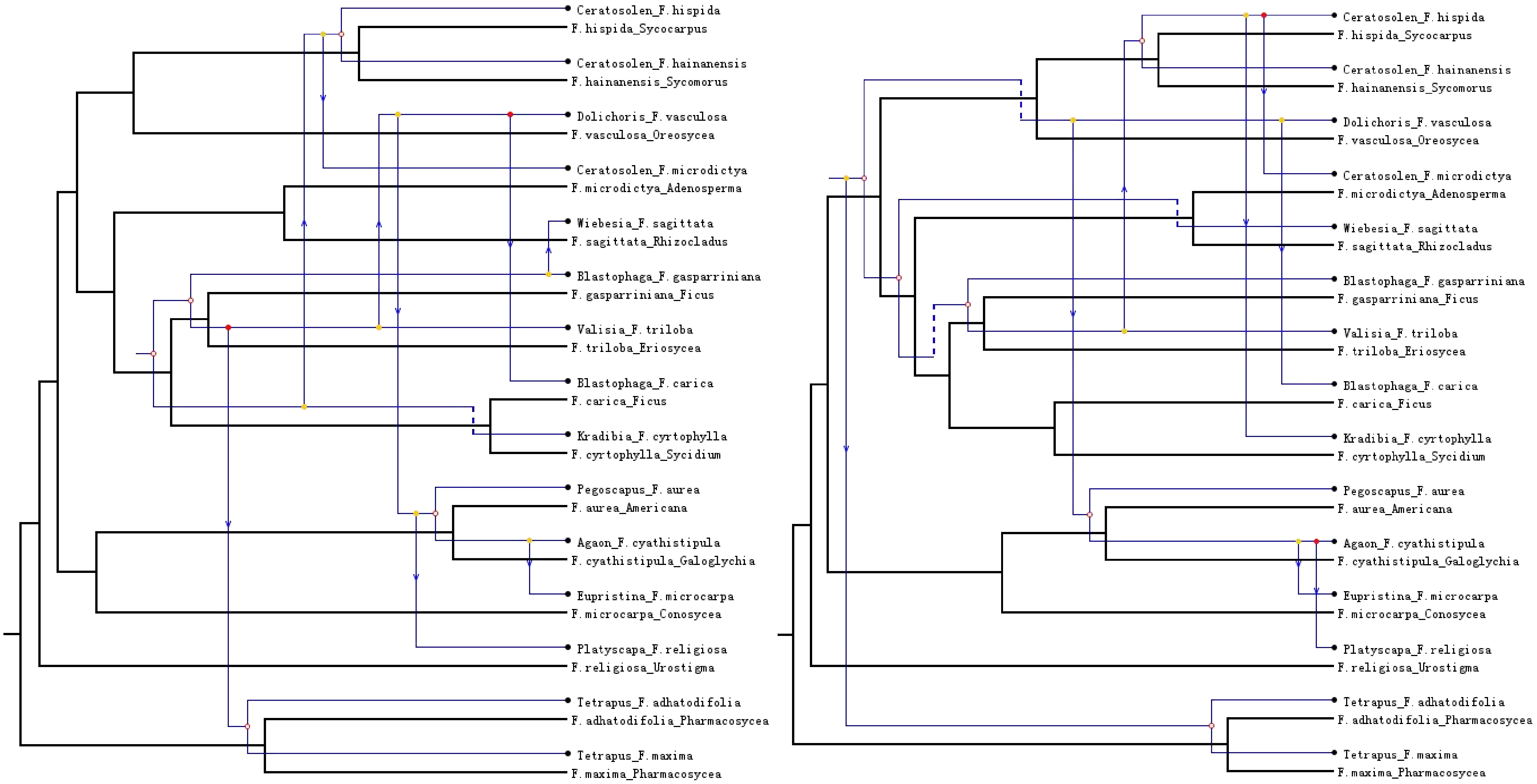

Supplementary Fig. 15: One of the equally most parsimonious reconciliations between *Ficus* mitochondrial phylogeny and pollinator phylogenies (Left: Cruaud et al. phylogeny (2012); Right: Machado et al. phylogeny (2001)) inferred with JANE. The black tree represents the mitochondrial genomic phylogeny and the blue tree the pollinator phylogeny. Hollow red circles on nodes represent codivergence events; filled colored dots and arrows on the blue lines represent events of associate host switch following duplication, while filled orange dots indicate the existence of another potential switch location with equal cost, and a filled red dot means that all other potential switch locations have higher cost. For the optimal cost settings used, refer to Supplementary Table 6.

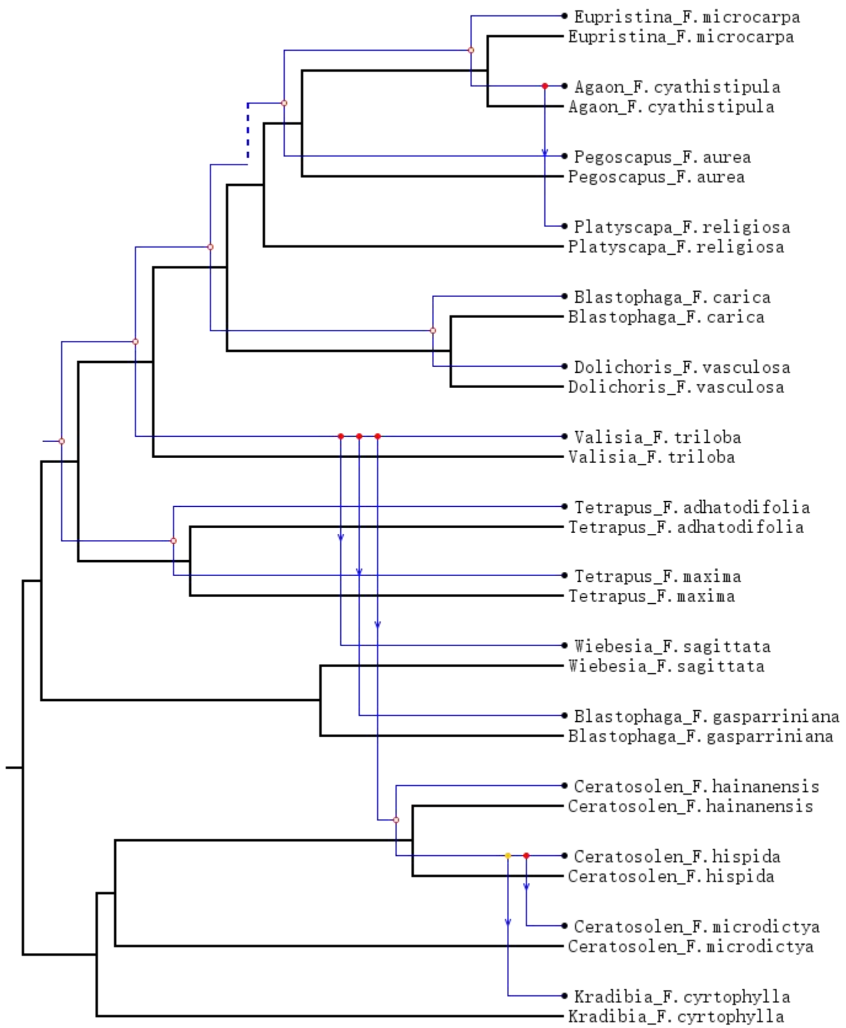

Supplementary Fig. 16: One of the equally most parsimonious reconciliations between two pollinator phylogenies inferred with JANE. The black tree represents pollinator phylogeny extracted from **Cruaud *et al*. phylogeny (2012),** and the blue tree that extracted from **Machado *et al*. phylogeny (2001)**. Hollow red circles on nodes represent codivergence events; filled colored dots and arrows on the blue lines represent events of associate host switch following duplication, while filled orange dots indicate the existence of another potential switch location with equal cost, and a filled red dot means that all other potential switch locations have higher cost. For the optimal cost settings used, refer to Supplementary Table 6. This result between the two wasp phylogenies just show that their phylogenetic topologies differ significantly, and do not indicate any other biological meaning.

Supplementary References

1 Zhang, X. et al. Genomes of the Banyan Tree and Pollinator Wasp Provide Insights into Fig-Wasp Coevolution. *Cell* **183**, 875–889, doi:10.1016/j.cell.2020.09.043 (2020).

2 Kerdelhue, C. & Rasplus, J. Y. The evolution of dioecy among *Ficus* (Moraceae): An alternative hypothesis involving non-pollinating fig wasp pressure on the fig-pollinator mutualism. *Oikos* **77**, 163-166 (1996).

3 Jousselin, E., Hossaert-McKey, M., Herre, E. A. & Kjellberg, F. Why do fig wasps actively pollinate monoecious figs? *Oecologia* **134**, 381-387 (2003).

4 Kjellberg, F. *et al.* Pollination mode in fig wasps: the predictive power of correlated traits. *Proc. R. Soc. B-Biol. Sci.* **268**, 1113-1121, doi:10.1098/rspb.2001.1633 (2001).

5 Harrison, R. D. & Yamamura, N. A few more hypotheses for the evolution of dioecy in figs (*Ficus*, Moraceae). *Oikos* **100**, 628-635 (2003).

6 Ramirez, W. Fig wasps - mechanism of pollen transfer. *Science* **163**, 580-& (1969).

7 Ramirez, W. Coevolution of Ficus and Agaonidae. *Ann Mo Bot Gard* **61**, 770-780 (1974).

8 Pellmyr, O. *et al.* Active pollination drives selection for reduced pollen-ovule ratios. *Am. J. Bot.* **107**, 164-170, doi:10.1002/ajb2.1412 (2020).

9 Jander, K. & Herre, E. Host sanctions and pollinator cheating in the fig tree-fig wasp mutualism. *Proc. R. Soc. B-Biol. Sci.* **277**, 1481-1488, doi:10.1098/rspb.2009.2157 (2010).

10 Jousselin, E., Rasplus, J. Y. & Kjellberg, F. Convergence and coevolution in a mutualism: Evidence from a molecular phylogeny of Ficus. *Evolution* **57**, 1255-1269 (2003).

11 Cook, J. M., Bean, D., Power, S. A. & Dixon, D. J. Evolution of a complex coevolved trait: active pollination in a genus of fig wasps. *J. Evol. Biol.* **17**, 238-246, doi:10.1111/j.1420-9101.2003.00683.x|ISSN 1010-061X (2004).

12 Cruaud, A. *et al.* An extreme case of plant-insect co-diversification: figs and fig-pollinating wasps. *Syst. Biol.* **61**, 1029-1047, doi:10.1093/sysbio/sys068 (2012).

13 Bruun-Lund, S., Clement, W. L., Kjellberg, F. & Rønsted, N. First plastid phylogenomic study reveals potential cyto-nuclear discordance in the evolutionary history of *Ficus* L. (Moraceae). *Mol. Phylogenet. Evol.* **109**, 93-104, doi:10.1016/j.ympev.2016.12.031 (2017).

14 Machado, C. A., Jousselin, E., Kjellberg, F., Compton, S. G. & Herre, E. A. Phylogenetic relationships, historical biogeography and character evolution of fig-pollinating wasps. *Proc. R. Soc. B-Biol. Sci.* **268**, 685-694 (2001).

15 Herre, E. A. *et al.* Molecular phylogenies of figs and their pollinator wasps. *J. Biogeogr.* **23**, 521-530, doi:DOI 10.1111/j.1365-2699.1996.tb00014.x (1996).

16 Gardner, E. M., Sarraf, P., Williams, E. W. & Zerega, N. J. C. Phylogeny and biogeography of Maclura (Moraceae) and the origin of an anachronistic fruit. *Mol. Phylogenet. Evol.* **117**, 49-59, doi:_10.1016/j.ympev.2017.06.021 (2017).

17 Weiß, C. L., Pais, M., Cano, L. M., Kamoun, S. & Burbano, H. A. nQuire: a statistical framework for ploidy estimation using next generation sequencing. BMC Bioinformatics 19, 122, doi:10.1186/s12859-018-2128-z (2018).
