# Supplementary material for: Genomic evidence of prevalent hybridization throughout the evolutionary history of the fig-wasp pollination mutualism
Source: Nat Commun. 2021 Feb 2;12:718. doi: 10.1038/s41467-021-20957-3 (PMC7854680; doi:10.1038/s41467-021-20957-3)
Supplement: Supplementary file 4 — Description of Additional Supplementary Files [file 41467_2021_20957_MOESM4_ESM.pdf]

Description of Additional Supplementary Files

Supplementary Data 1: Detailed results of four-taxon ABBA-BABA D-statistics tests for hybridization detected at different phylogenetic levels in *Ficus*

Supplementary Data 2: Major discordance in splits groups and inference of hybridization with BUCKy

Supplementary Data 3: Geographic distribution of 59 figs studied by Bruun-Lund et al. 2017
