# Supplementary material for: Genomic evidence of prevalent hybridization throughout the evolutionary history of the fig-wasp pollination mutualism
Source: Nat Commun. 2021 Feb 2;12:718. doi: 10.1038/s41467-021-20957-3 (PMC7854680; doi:10.1038/s41467-021-20957-3)
Supplement: Supplementary file 2 — Peer Review File [file 41467_2021_20957_MOESM2_ESM.pdf]

Reviewers' comments:

Reviewer #1 (Remarks to the Author):

In their manuscript submitted for consideration in Nature Communications, Wang et al use extensive genome-scale data to reconstruct phylogenetic relationships among figs (plants in the genus Ficus, the family Moraceae), and to evaluate evidence for past hybridization and host switching by fig wasp pollinators. Using summary statistics (Patterson's D), methods for parameter estimation that evaluate phylogenetic conflicts between genes and between genomic compartments (nuclear, plastid, mitochondrial), the authors find support for recurrent hybridization in the evolutionary history of the figs. In addition, the authors used event-based analyses to evaluate evidence for both hybridization and host switching by pollinators, and find support for both.

The genus Ficus is a diverse, globally distributed group, and in many ecosystems figs serve as a keystone species that is involved in ecological interactions with many other organisms. In addition, the fig / fig wasp system is perhaps the best-known example of an obligate pollination mutualism (aka a nursery pollination system) in which pollinators lay their eggs in the flowers of their host and engage in active, 'deliberate' pollination of their host. These nursery pollination systems are considered important models for studies of coevolution and mutualistic interactions generally. Understanding the ecology and evolution of the genus is thus of importance both from the perspective of pure scientific curiosity, and for applied conservation biology.

For many years now there has been considerable debate within the coevolution community about the extent to which figs, and nursery pollination systems generally, exhibit strict 'one-to-one' host specificity, and whether they have evolved through 'cospeciation'. That is, it has been unclear to what extent each species of fig is served by a single pollinator, and whether each species of pollinator associates with a single species of fig. Likewise, an area of significant interest has been whether the two groups speciate in parallel, with each speciation event in the pollinators prompting speciation in the host (or vice versa).

The authors bring genetic data that are unprecedented in scale to this question, and find compelling evidence of recurrent hybridization, suggesting both a lack of complete host specificity and deviations from strict cospeciation. The work should therefore be of interest to a wide range of ecologists, evolutionary biologists, and those interested in interactions more generally. Furthermore, the paper potentially offers the definitive conclusion about the evolutionary history of figs and their pollinators, and provides a model for how similar studies might be conducted in other nursery pollination systems.

The methods used to approach these problems are the accepted tools within the field, and are applied appropriately. However, I see two areas in which additional evidence may strengthen the paper, both of which involve comparisons among the various results. These are as follows:

First, I think that more could be done with the information about the geographic ranges of the figs. The authors identify an intriguing pattern in which hybridization appears to have occurred among lineages that are sympatric (that is, they occupy the same habitats, presenting the opportunity for hybridization). This is also supported by a pattern of 'phylogenetic clustering' of fig species from the same regions in an evolutionary analysis of chloroplast genome sequence data. This observation makes biological sense, as the chloroplast is typically passed maternally (in seeds) and thus should show greater geographic structure than nuclear genes, which may be dispersed over long distances by pollinators. HOWEVER, if I understand correctly, the inference that hybridization happens among sympatric species is based entirely on CURRENT geographic distributions. A further analysis that would substantially strengthen the paper would be to reconstruct the ancestral distributions of the plants, and evaluate whether different lineages were sympatric at the time that hybridization events appear

to have occurred (Note, because both the analyses of hybridization events and reconstructions of geographic distribution refer to the same phylogeny, a molecular clock analysis would not be necessary).

Second, I believe that there are opportunities for comparing the hybridization events and host switches by pollinators. An appealing aspect of this study is that it evaluates patterns of co-evolutionary history across different scales – genes within genomes, parasites (pollinators) within hosts, species within geographic regions. The parallels between horizontal gene flow, host switching, and dispersal, have fascinated evolutionary biologists for decades. In this manuscript, the authors identify – in a single study – evidence for both hybridization (horizontal gene flow) and host switching by pollinators (a potential mechanisms for hybridization and horizontal gene flow). However, as far as I can tell, there is no direct comparison of those results. That is, it would be intriguing to know if the recovered hybridization events correspond to specific host switching events by the pollinators. Can we say that a particular hybridization event was contemporaneous with a pollinator host switch among the species that hybridized? I may be missing something, but I see no reason that such an analysis is not possible.

Reviewer #2 (Remarks to the Author):

This manuscript presents an extensive tri-genome dataset for a modest sample of Ficus species, with the goal of evaluating the role of hybridization in this history of this economically and evolutionarily important lineage. While there is much to be impressed about with this paper, I am not convinced that it achieves its goals. I have two broad concerns, in particular. First, the narrative relies on their being an extensive history of hybridization in the group, and I'm not sure about the evidence for that. For example, the incongruence between the species tree ("tetrad tree") and the primary concordance tree suggests to me that this is a tricky dataset, potentially with a lot of noise, and that the incongruence should not be attributed quickly to hybridization. The ABBA-BABA tests are potentially the best examination of hybridization, and I would like them to be much more thoroughly described (both their implementation and their results), prior to being very comfortable with the conclusions drawn here. I could be misunderstanding some of the functionality of BUCKy (and welcome being corrected by the authors), but my understanding is that it doesn't model hybridization (or ILS) specifically—concordance, or lack thereof, could be due to a host of factors. For example, I would want to see more attention paid to investigating the quality of the sequence data – could be there be a background rate of index-swapping in the illumina runs that are giving the impression of hybridization? Phrased very generally, I think this paper suffers from too much data, in the sense that it is then difficult to clearly discern what is going on, to distinguish stochastic from systematic error, etc.

My second broad concern is about the macroevolutionary narrative—there is no evidence presented to link the inferences in this paper to diversification dynamics, and no evidence presented for why the diversification rates of Ficus are in any way unusual. Any connection between the inferred patterns of hybridization and diversification dynamics and adaptive processes seem highly conjectural. Basically, if the results of this study are correct, figs hybridize a lot —there's no evidence that this hybridization has an impact on diversification, let alone an adaptive function in response to environmental change.

More specific comments:
-I'm a little concerned about using cultivated plants for a study like this—could there be artificial hybrids involved? It's easy for such a thing to happen (and for provenance and identification information to be swapped) in a botanical garden
-In general, the description of the methods needs elaboration
-The outgroup is potentially inadequate, especially given that the main thrust of the paper depends on getting the ingroup relationships (and thus the root position) correct. I'd suggest using multiple accessions for the outgroup, including lineages more closely related to Ficus.
-I don't think the JANE analyses are appropriate for looking at incongruence among the genomic

compartments.
-Fig1c – why are coalescent units shown rather than a metric of support, as in the other trees in this figure?
-Fig. 2 – the red and blue lines were “generated” by BUCKy and the ABBA-BABA test, respectively? How was that done?
-Table 1 – following my general comments on the cospeciation model approach, I would suggest that the strictly genomic comparisons (nuclear-mitochondrial, etc) be removed from this table. The cospeciation model only applies to co-evolving species—in this case, the figs and the wasps

-Full voucher information (including the herbarium where the specimen is deposited) need to be provided for each plant sequenced. In addition, the sequence data need to be deposited in a publically accessible archived, and ideally the phylogenies too (TREEbase or OpenTree, etc).

Additional comments are included as tracked changes in the attached version of the manuscript.

Reviewer #3 (Remarks to the Author):

Overview:
The authors use complete genome sequences of representatives of major Ficus lineages to demonstrate that introgression and potentially hybrid speciation has occurred frequently within the group. This complex history of introgression is likely associated with pollinator host switching and ultimately diversification of the genus. Patterns of introgression were identified with deviations from equal minor quartet frequencies among gene trees and genome-wide ABBA-BABA tests. Strong cyto-nuclear discordance was also observed between chloroplast/mitochondrial and nuclear genomes. Cophylogeny analyses suggests that co-speciation alone is not sufficient, and that some degree of host-switching is necessary, which is coincident with the fig and fig-wasp cophylogeny. A speciation mechanism for Ficus is proposed with applications to other plant-pollinator associations too.

Major Comments:
Normally I do not belabor phylogenetic methods, but because the cophylogeny analyses are highly dependent on the chloroplast and mitochondrial trees, I would like to see that the results are robust to phylogenetic estimation method. Specifically, I find the use of invariants (i.e. tetrad) for the chloroplast and mitochondrial trees to be an odd choice- organellar genomes are single loci and I would thus argue treating them as unlinked SNP data for an MSC method inappropriate. Additionally, although invariant methods are consistent, they can require large numbers of SNPS to perform well for some phylogenetic problems such as large population sizes (e.g. Washer and Kubatko 2019). The organellar genomes would be best treated with concatenated and partitioned ML. If there is some reference and annotated chloroplast and mitochondrial genomes, I would think extracting potential partitions (i.e. coding vs noncoding) from the gff would be straightforward.

I have concerns about how loci are delimited for the BUCKy analyses. The loci are windows of the nuclear genome with 1,100 SNPS each – these must span large physical distances, right? I might be mistaken, but there is no information on the reference genome and how these sliding windows are oriented physically to judge otherwise. I would expect recombination has occurred with some frequency within these large genomic regions and treating them as independent loci for BUCKy and the introgression tests strikes me as though it will introduce some systematic biases. Why not use MDL (Ané 2011), or sliding windows of a fixed length (e.g. Figueiró et al. 2017)? Either way, I would like some justification on why treating large concatenated genomic regions as independent loci here would not be misleading – that a relatively small proportion of sites in each window is dominating the posterior distribution of topologies (e.g. Shen et al. 2017).

The divergence time estimation as described on lines 490-494 is strange. The population tree from

BUCKy estimates branch lengths in coalescent units (Number of Generations/2Ne). r8s estimates absolute substitution rates and divergence times from a tree that has branch lengths in expected substitutions per site. I do not see how using coalescent units for estimating divergence times is a reasonable approximation. Why not just re-estimate branch lengths with ML and then use r8s (The penalized likelihood function used in R here)? I can appreciate that the divergence time estimation is not a central component of the results here, but normally you would want to determine a good rate smoothing parameter heuristically with cross-validation too.

It is difficult to determine where the BUCKy tests for over-representation of a minor quartet and D-stats agree based on Figure 2. Also, but from what I can tell, the F. vasculosa ¬– F. sagittata introgression event from BUCKy in Fig. 2a seems absent from the D-stats in Fig. 2b. Although, the D-stat result is quite strong based on Supplementary Table S6. Similarly, I do not see introgression between the common ancestor of subgenus Sycomorus and F. carica in Fig. 2a represented by the D-stats in Fig. 2b. In short, I think Fig. 2a and Fig 2b can be reconciled into a single figure with links that show events detected by both BUCKy and D-stats and those detected with D-stats alone.

The Jane analyses are critical to the argument that co-phylogeny alone is not a good model for fig and fig-wasp coevolution and connecting host switching to introgression among fig species. But, I am also not convinced that some of the inferred costs for co-phylogeny between nuclear and organellar genomes is not due to poor phylogenetic estimation. Jane should be able to do two permutation tests to check that the cost scores and host switching events would not be expected by chance alone – There is a random tip-mapping procedure as well as a test for when the parasite (i.e. cp, mt, or pollinator) tree is random. I would like to see that the inferred costs and host switches would not be expected by chance alone.

Minor Comments:

The comparison of the larger 5-gene nuclear phylogeny and 59 cp genome phylogeny on Fig. 5 and lines 198-209 is difficult to interpret. I really cannot follow any claim of cp-nuclear discordance given Figure 5. Why not parse out only the taxa represented in the cp tree from the nuclear dataset? That would make the comparison of the two trees easier for the reader. In general, I do not think this section adds much to the results because it does not do draw new insights from the data compared to (Cruaud et al. 2012) unless the results can be better integrated with the 15 taxon analyses.

It is not clear why the 5-gene nuclear phylogeny is "improved" if the methods and data were the same. The re-analysis brings Urostigma together as a monophyletic group with 100% bootstrap support, but this would be due to the constraint imposed by the 15-taxon backbone tree (F. religiosa, F. aurea, and F. cyathistipula). Thus, the tree is practically forced to have 100% bootstrap support for Urostigma. I am guessing than an ML tree was re-estimated using GTR-CAT with RAxML and the 15 taxon species tree was used as a constraint. Please elaborate and clarify in the methods. Cruaud et al. (2012) used several approaches for phylogenetic estimation and citing "same procedures" on line 579 is ambiguous.

As a complementary analysis to the quartet-based tests with BUCKy, consider passing the quartet concordance factors to PhyloNetworks (Solis-Lemus et al. 2017). Estimating an explicit network might help resolve some of the ambiguity in where introgression occurred on the phylogeny. I think this would require minimal effort given the quartet concordance factors are already estimated. The authors allude to this on lines 374-382 of the discussion anyway.

I do not see the fig reference genome cited anywhere. Is it Mori et al. (2017).

Are all of the Ficus species sequenced here diploids? Are any of the species potentially allopolyploids?

References:

Ané C. 2011. Detecting phylogentic breakpoints and discordance from genome-wide alignments for species tree reconstruction. Genome Biol Evol. 3:246-258.

Cruaud A, Rønsted N, Chantarasuwan B, Chou LS, Clement WL, Couloux A, Cousins B, Genson G, Harrison RD, Hanson PE, et al. 2012. An extreme case of plant-insect codiversification: figs and fig-pollinating wasps. Syst Biol. 61:1029-1047.

Figueiró HV, Li G, Trindade FJ, Assis J, Pais F, Fernandes G, Santos SHD, Huhes GM, Komissarov A, Antunes A, et al. 2017. Genome-wide signatures of complex introgression and adaptive evolution in the big cats. Sci Adv. 3:e1700299. doi: https://doi.org/10.1126/sciadv.1700299.

Mori K, Shirasawa K, Nogata H, Hirata C, Tashiro K, Habu T, Kim S, Himeno S, Kuhara S, Ikegami H. 2017. Identification of RAN1 orthologue associated with sex determination through whole genome sequencing analysis in fig (Ficus carica L.). Sci Rep. 7:41124. Doi: https://doi.org/10.1038/srep41124.

Solis-Lemus C, Bastide P, Ané C. 2017. PhyloNetowks: A package for phylogenetic networks. Mol Biol Evol. 34:3292-3298.

Shen X-X, Hittinger CT, Rokas A. 2017. Contentious relationships in phylogenomic studies can be driven by a handful of genes. Nat Ecol Evol. 1:0126. doi: https://doi.org/10.1038/s41559-017-0126.

Washer M, Kubatko L. 2019. Consistency of SVDQuartets and maximum likelihood for coalescent based species tree estimation. bioRxiv. doi: https://doi.org/10.1101/523050.

Point-by-point replies (text in blue) under each comment (text in black)

##

Reviewers' comments:

Reviewer #1 (Remarks to the Author):

In their manuscript submitted for consideration in Nature Communications, Wang et al use extensive genome-scale data to reconstruct phylogenetic relationships among figs (plants in the genus Ficus, the family Moraceae), and to evaluate evidence for past hybridization and host switching by fig wasp pollinators. Using summary statistics (Patterson's D), methods for parameter estimation that evaluate phylogenetic conflicts between genes and between genomic compartments (nuclear, plastid, mitochondrial), the authors find support for recurrent hybridization in the evolutionary history of the figs. In addition, the authors used event-based analyses to evaluate evidence for both hybridization and host switching by pollinators, and find support for both.

The genus Ficus is a diverse, globally distributed group, and in many ecosystems figs serve as a keystone species that is involved in ecological interactions with many other organisms. In addition, the fig / fig wasp system is perhaps the best-known example of an obligate pollination mutualism (aka a nursery pollination system) in which pollinators lay their eggs in the flowers of their host and engage in active, 'deliberate' pollination of their host. These nursery pollination systems are considered important models for studies of coevolution and mutualistic interactions generally. Understanding the ecology and evolution of the genus is thus of importance both from the perspective of pure scientific curiosity, and for applied conservation biology.

For many years now there has been considerable debate within the coevolution community about the extent to which figs, and nursery pollination systems generally, exhibit strict 'one-to-one' host specificity, and whether they have evolved through 'cospeciation'. That is, it has been unclear to what extent each species of fig is served by a single pollinator, and whether each species of pollinator associates with a single species of fig. Likewise, an area of significant interest has been whether the two groups speciate in parallel, with each speciation event in the pollinators prompting speciation in the host (or vice versa).

The authors bring genetic data that are unprecedented in scale to this question, and find compelling evidence of recurrent hybridization, suggesting both a lack of complete host specificity and deviations from strict cospeciation. The work should therefore be of interest to a wide range of ecologists, evolutionary biologists, and those interested in interactions more generally. Furthermore, the paper potentially offers the definitive conclusion about the evolutionary history of figs and their pollinators, and provides a model for how similar studies might be conducted in other nursery pollination systems.

Reply: Thank you for your positive evaluation concerning the importance and value of our study.

The methods used to approach these problems are the accepted tools within the field, and are applied appropriately. However, I see two areas in which additional evidence may strengthen the paper, both of which involve comparisons among the various results. These are as follows:

First, I think that more could be done with the information about the geographic ranges of the figs. The authors identify an intriguing pattern in which hybridization appears to have occurred among lineages that are sympatric (that is, they occupy the same habitats, presenting the opportunity for hybridization). This is also supported by a pattern of 'phylogenetic clustering' of fig species from the same regions in an evolutionary analysis of chloroplast genome sequence data. This observation makes biological sense, as the chloroplast is typically passed maternally (in seeds) and thus should show greater geographic structure than nuclear genes, which may be dispersed over long distances by pollinators. HOWEVER, if I understand correctly, the inference that hybridization happens among sympatric species is based entirely on CURRENT geographic distributions. A further analysis that would substantially strengthen the paper would be to reconstruct the ancestral distributions of the plants, and evaluate whether different lineages were sympatric at the time that hybridization events appear to have occurred (Note, because both the analyses of hybridization events and reconstructions of geographic distribution refer to the same phylogeny, a molecular clock analysis would not be necessary).

Reply: Thanks very much for this constructive suggestion. In the revised version, we estimated the divergence times of main clades within *Ficus* using the MCMCTREE program in PAML 4.9a (Yang

2007) and reconstructed the ancestral geographic distributions of the lineages to which the surveyed fig species belong, using the R package “BioGeoBEARS (Matzke 2014). We consider the implications of both Eurasian (more consistent with the Astral analyses) and Gondwanan origins of *Ficus* (more consistent with the BUCKy analyses), both of which suggest ancestral distributions of most fig clades that are compatible with their coexistence for periods sufficient for sympatric hybridization events to occur. This long period of ancient sympatry makes it easier to understand the inferred hybridization events between lineages that are currently allopatric. Further, we emphasize what data are needed to distinguish between what we consider the currently unresolved question of site and time of origin of *Ficus*.

Text related to these new analyses was added in the Methods, Results and Discussion sections of the revised manuscript.

Yang, Z. H. PAML 4: Phylogenetic analysis by maximum likelihood. *Mol. Biol. Evol.* **24**, 1586-1591, doi:10.1093/molbev/msm088 (2007).

Matzke, N. J. Model selection in historical biogeography reveals that founder-event speciation is a crucial process in island clades. *Syst. Biol.* **63**, 951-970, doi:10.1093/sysbio/syu056 (2014).

Second, I believe that there are opportunities for comparing the hybridization events and host switches by pollinators. An appealing aspect of this study is that it evaluates patterns of co-evolutionary history across different scales – genes within genomes, parasites (pollinators) within hosts, species within geographic regions. The parallels between horizontal gene flow, host switching, and dispersal, have fascinated evolutionary biologists for decades. In this manuscript, the authors identify – in a single study – evidence for both hybridization (horizontal gene flow) and host switching by pollinators (a potential mechanisms for hybridization and horizontal gene flow). However, as far as I can tell, there is no direct comparison of those results. That is, it would be intriguing to know if the recovered hybridization events correspond to specific host switching events by the pollinators. Can we say that a particular hybridization event was contemporaneous with a pollinator host switch among the species that hybridized? I may be missing something, but I see no reason that such an analysis is not possible.

Reply: Thanks very much for this important and constructive suggestion, which we have followed up with additional analyses.

In this revised version, we not only confirmed that both hybridization and pollinator host switches have been frequent, but also that they largely correspond to each other. Comparing the patterns of results for hybridization and for pollinator host switches, we found that both types of events (hybridization: Fig. 2; pollinator host switches: Fig. 5B, supplementary Figs. 11-14) involved all main clades of *Ficus*. Using the host-switch pattern based on the highly supported Astral tree for *Ficus* and the most recently published and widely accepted phylogeny for fig wasps (Cruaud et al. 2012) as examples, all eight pollinator host-switch events detected are matched by hybridization events detected between the corresponding host fig clades (Supplementary Table 9). Furthermore, we have also supplied a summary table (Supplementary Table 10) showing the main hybridization events inferred among main *Ficus* clades, and evidence for the corresponding inferred pollinator host-switch events. Most hybridizations (18 of 22) listed inferred between main *Ficus* clades are accompanied by corresponding inferred pollinator host switches. Finally, we added a new Fig. 5 showing detailed results of cophylogenetic reconciliations of both *Ficus* nuclear-chloroplast and *Ficus* nuclear-pollinator phylogeny pairs. These results also highlight the similarity between the general pattern of hybridization and that of pollinator host switches between *Ficus* main clades.

Text related to these new analyses was added in the Methods, Results and Discussion sections of the revised manuscript.

Cruaud, A. *et al.* An extreme case of plant-insect co-diversification: figs and fig-pollinating wasps. *Syst. Biol.* **61**, 1029-1047, doi:10.1093/sysbio/sys068 (2012).

Reviewer #2 (Remarks to the Author):

This manuscript presents an extensive tri-genome dataset for a modest sample of Ficus species, with the goal of evaluating the role of hybridization in this history of this economically and evolutionarily important lineage. While there is much to be impressed about with this paper, I am not convinced that it achieves its goals. I have two broad concerns, in particular. First, the narrative relies on their being an extensive history of hybridization in the group, and I’m not sure about the evidence for that. For example, the incongruence between the species tree (“tetrad tree”) and the primary concordance tree

suggests to me that this is a tricky dataset, potentially with a lot of noise, and that the incongruence should not be attributed quickly to hybridization.

I could be misunderstanding some of the functionality of BUCKy (and welcome being corrected by the authors), but my understanding is that it doesn't model hybridization (or ILS) specifically—concordance, or lack thereof, could be due to a host of factors. For example, I would want to see more attention paid to investigating the quality of the sequence data – could be there be a background rate of index-swapping in the illumina runs that are giving the impression of hybridization? Phrased very generally, I think this paper suffers from too much data, in the sense that it is then difficult to clearly discern what is going on, to distinguish stochastic from systematic error, etc.

Reply: Thanks very much for your criticism. Based on your comments, we have attempted to clarify the relevant parts of the paper.

In response to your query about the quality of sequence data, we make it clear that we used strict criteria to filter the re-sequencing reads generated in the Illumina X10 platform. To avoid potential DNA contamination via sequencing, such as index-swapping, we built dual-indexed libraries with unique indexes for each sample. The dual-indexes contain a total of 16 bases that were inserted into the flanking regions of the target DNA fragments. This ensured our ability to unambiguously separate DNA sequences pooled from different libraries and thereby avoid potential index hopping. In addition, raw reads that had any mismatch with index sequences were clustered as undetermined sequences and removed from our analysis.

For further information on the methods we used, we refer the reviewer to detailed information supplied by Illumina: "Effects of Index Misassignment on Multiplexing and Downstream Analysis" (https://www.illumina.com/content/dam/illumina-marketing/documents/products/whitepapers/index-hopping-white-paper-770-2017-004.pdf?linkId=36607862). We also attached this Illumina file within the Source Data of this manuscript (https://datadryad.org/stash/share/AyiHbELHaKUt9cfN1c1xk-kHk_ErG2U_iYavoiU-MLo).

Furthermore, our results revealed multiple ancient hybridization events involving species sequenced at different times and by different sequencing facilities. The two species *F. microcarpa* and

F. hispida were sequenced independently from all other species for de novo genome assemblies, by different sequencing providers (BioMarker Company located in Beijing city, and Annoroad Company located in Yiwu city, respectively). There is thus no possibility of data pollution between these two species and between either of them and the other fig species, simply because they were sequenced independently (in space and time). The multiple hybridization events detected between *F. microcarpa* and *F. hispida*, and between each of them and other studied fig species, allow us to reject the hypothesis of sequencing pollution. More information on the data used to assemble the genomes of *F. microcarpa* and *F. hispida* will appear in a companion paper presently under review (Zhang *et al.*, “Genomes of banyan trees provide insights into fig-wasp co-evolution”).

Importantly, the *F. microcarpa* reference genome is of high quality. BUSCO analysis showed 93.5% genome completeness. PASA-assembled transcripts based on RNA-seq reads were aligned to the reference genome and the result showed that more than 99.9% of transcripts could be aligned to the assembly with 99.88% single base accuracy for our assembly. In addition, 97.98% of Illumina reads can be mapped to the reference genome, covering 99.23% of assembled sequences, indicating a high-quality reference assembly. The *Ficus microcarpa* reference genome has been deposited in the Genome Sequence Archive BIG Data Center (http://bigd.big.ac.cn/gsa) under the accession number of GWHABKV00000000. We added the above information in Supplementary Note 1.

For the two other cases you mentioned (incongruence between the species tree and the primary concordance tree, and the ability of BUCKy to detect hybridization), there appears to be some misunderstanding.

First, we did not mention any incongruence between the “tetrad tree” and the “primary concordance tree”; the two trees are in fact similar in topology with little incongruence. We mentioned this in the results section of the initial submission as follows (lines 119-121): “the main phylogenetic topologies of the species trees based on nuclear genomic data were similar as determined using three different reconstruction methods, namely, tetrad tree, primary concordance tree, and population tree (Fig.1, Fig.S1A-C)”.

Second, regarding the capacity of BUCKy to detect hybridization, we agree that the primary concordance tree cannot be used directly for this purpose. The lower concordance factor value only indicates the serious gene-tree discordance, giving no information about the possible cause. We

mentioned this in the original submission, e.g., lines 148-150: “The primary concordance tree (Fig. 1B, Fig. 2) inferred with BUCKy[44], possesses relatively low concordance factor (CF) values for most nodes, indicating extensive gene-tree discordance.” We use the results of BUCKy to infer hybridization by further testing whether the distribution of gene-tree frequencies is compatible with that expected under a neutral coalescent process (incomplete lineage sorting, ILS), or whether other biological process must also be invoked (e.g., hybridization, horizontal gene transfer) (Baum 2007, Degnan and Rosenberg 2009). We infer that hybridization must be invoked. We apologize for having failed to explain this more clearly. In the revised version, we have improved our description of methods in Lines 593-609.

Baum, D. A. Concordance trees, concordance factors, and the exploration of reticulate genealogy. *Taxon* **56**, 417-426, doi : 10.1002/tax.562013 (2007).

Degnan, J. H. & Rosenberg, N. A. Gene tree discordance, phylogenetic inference and the multispecies coalescent. *Trends Ecol. Evol.* **24**, 332-340, doi:10.1016/j.tree.2009.01.009 (2009).

My second broad concern is about the macroevolutionary narrative—there is no evidence presented to link the inferences in this paper to diversification dynamics, and no evidence presented for why the diversification rates of Ficus are in any way unusual. Any connection between the inferred patterns of hybridization and diversification dynamics and adaptive processes seem highly conjectural. Basically, if the results of this study are correct, figs hybridize a lot —there’s no evidence that this hybridization has an impact on diversification, let alone an adaptive function in response to environmental change.

Reply: Thanks for this insightful comment. Although we demonstrate that there has been pervasive hybridization throughout *Ficus* evolution, we agree that there is still a gap between this demonstration and the mechanisms of diversification in *Ficus*. *Ficus* is among the most diverse woody plant genera (~800 described species) and is often the most diverse genus in tropical forests (Harrison 2005, Berg and Corner 2005). Uncovering the full diversification mechanism of *Ficus* will be a great contribution but also a big challenge.

We considered trying to develop a hypothesis relating the pattern of frequent hybridization and pollinator host switches throughout *Ficus* evolution that we detected in this study to mechanisms of

diversification. However, as you mention here and elsewhere in your review, there are still many gaps, and we agree that the patterns we uncovered here do not show how hybridization and host switching could increase speciation rate. Net diversification rate is also influenced by extinction rate, about which our data are also silent.

In the revised version, we deleted almost all discussion of potential mechanisms of diversification, focusing simply on explaining how hybridization and pollinator host switches can occur in the supposedly obligate and specific interactions between *Ficus* and their pollinating wasps. Together with what is known about the chemical ecology of pollinator attraction to *Ficus*, published reports documenting numerous exceptions to host specificity, and the occurrence of hybrid *Ficus* seedlings and even adult plants, suggest a scenario in which periods of reproductive isolation mediated by pollinator specificity alternating with hybridization events mediated by pollinator host-switching can lead to novel fig/wasp associations. This insight could be an important first step toward understanding diversification mechanisms, not only in fig-fig wasp mutualisms, but also in other pollination mutualisms, as we discuss in the closing paragraph. Currently, we agree that this is a story to be developed later. See the Discussion section for details of our treatment of this aspect.

Harrison, R. D. Figs and the diversity of tropical rainforests. *Bioscience* **55**, 1053-1064, doi : 10.1641/0006-3568(2005)055[1053:FATDOT]2.0.CO;2 (2005).

Berg, C. C. & Corner, E. J. H. in *Flora Malesiana Series I -Seed Plants* Vol. 17 (ed H.P. Nooteboom) 1-702 (Nationaal Herbarium Nederland, 2005).

More specific comments:

-I’m a little concerned about using cultivated plants for a study like this—could there be artificial hybrids involved? It’s easy for such a thing to happen (and for provenance and identification information to be swapped) in a botanical garden

Reply: Thanks for this comment, which provides us with an opportunity to further clarify our sampling strategy and how we avoided this potential problem. First, samples for many species were collected not from the botanical garden itself, but in a large area (315 ha) of mature rain forest

adjacent to the garden. Second, individuals of those species that were sampled in the garden originated from seeds or seedlings collected from these nearby natural habitats or introduced from abroad (e.g., *F. aurea* from the Neotropics and *F. cyathistipula* from South Africa), as documented by the garden's records. Third, other species were directly sampled from natural habitats in distant countries (e.g., *F. adhatodifolia* and *F. maxima* from Brazil, and *F. microdictya* from Papua New Guinea). Finally, all sampled trees or specimens were carefully identified following morphological criteria by our taxonomists at XTBG, and the biology of most species has been intensively studied for many years by *Ficus* research groups in XTBG, in collaboration with several international groups from France, the USA, the UK, and Brazil, and elsewhere. For all these reasons, we are highly confident that we have avoided sampling artificial hybrids. We have made the above points clear in the Methods section of the revised manuscript.

-In general, the description of the methods needs elaboration

Reply: We improved and expanded the presentation of the Methods section.

-The outgroup is potentially inadequate, especially given that the main thrust of the paper depends on getting the ingroup relationships (and thus the root position) correct. I'd suggest using multiple accessions for the outgroup, including lineages more closely related to Ficus.

Reply: Thank you for this suggestion, which we accepted. In the revised version, **we re-ran almost all analyses with two outgroup species**. The newly added species (*Antiaris toxicarica*) is much more closely related to *Ficus* than is *Morus*, the sole outgroup used in the initial submission, based on recently published work on Moraceae phylogeny (see Fig. 2 in Gardner et al. 2017). Based on the new dataset with two outgroups, the Astral species trees inferred with four types of nuclear windows datasets yielded a more robust *Ficus* phylogeny, with high support values at most nodes.

Gardner, E. M., Sarraf, P., Williams, E. W. & Zerega, N. J. C. Phylogeny and biogeography of *Maclura* (Moraceae) and the origin of an anachronistic fruit. *Mol. Phylogenet. Evol.*, doi:10.1016/j.ympev.2017.06.021 (2017).

-I don't think the JANE analyses are appropriate for looking at incongruence among the genomic compartments.

Reply: JANE analysis has been widely used to study phylogenetic incongruence among associated lineages, especially parasite and host lineages. However, its utility has not been limited to this. According to the original publication and the official website of JANE software (see below), JANE was designed for several kinds of historical association problems, including reconciliation of species trees (nuclear) and gene trees (e.g., chloroplast tree and mitochondrial tree), as was conducted in this study.

However, the point you make is helpful in our case, because the terms we used in describing JANE results in the initial submission ("cospeciation", "host switches") are not strictly suitable when applied to different genomic compartments of a single species. We have changed these terms to "codivergence" and "associate switches", respectively.

The following passage from the abstract of the paper that first introduced JANE software (Conow et al. 2010) shows that our application of JANE to compare nuclear and organelle trees is appropriate: "This paper describes the theory and implementation of a new software tool, called Jane, for the study of historical associations. This problem arises in parasitology (associations of hosts and parasites), molecular systematics (associations of orderings and genes), and biogeography (associations of regions and orderings)."(Conow, C., Fielder, D., Ovadia, Y. & Libeskind-Hadas, R. Jane: a new tool for the cophylogeny reconstruction problem. *Algorithms Mol. Biol.* 5, doi:10.1186/1748-7188-5-16 (2010).)

The first paragraph in the Jane website (https://www.cs.hmc.edu/~hadas/jane/) is also instructive: "Jane is a software tool for the cophylogeny reconstruction problem. …… Note that while this documentation refers to the trees as "host" and "parasite," Jane is also used to reconcile other pairs of trees such as species trees and gene trees."

-Fig1c – why are coalescent units shown rather than a metric of support, as in the other trees in this figure?

Reply: In the revised version, **we have deleted those results**. In the initial submission, Figure 1c (population tree inferred with BUCKy) only gave coalescent units as node labels, with no support values being supplied. Nonetheless, this population tree shows the same topology as the primary concordance tree (with support values, also inferred with BUCKy, Figure 1b of the initial submission). We thus presented the coalescent units in Figure 1c just to give more information about divergence times of lineages within *Ficus*. In the revised version, we have replaced these illustrations and associated text with a more formal estimation of divergence times using the MCMCTREE program of PAML 4.9a (Yang 2007).

Yang, Z. H. PAML 4: Phylogenetic analysis by maximum likelihood. Mol. Biol. Evol. 24, 1586-1591, doi:10.1093/molbev/msm088 (2007).

-Fig. 2 – the red and blue lines were “generated” by BUCKy and the ABBA-BABA test, respectively? How was that done?

Reply: In the first version, these lines were artificially plotted onto the *Ficus* species tree based on the results of BUCKy analysis and ABBA-BABA tests, for a better visualization of results for detection of hybridization based on the two methods, while the detailed information was shown in Supplementary Tables. In the revised version, we followed the same strategy to show all the hybridization events detected by three methods (ABBA-BABA *D*-statistics, Bayesian Concordance, and PhyloNetworks) in Fig. 2, and put the detailed results in supplementary files (Supplementary Tables 6, 7 and Supplementary Fig. 6).

-Table 1 – following my general comments on the cospeciation model approach, I would suggest that the strictly genomic comparisons (nuclear-mitochondrial, etc) be removed from this table. The cospeciation model only applies to co-evolving species—in this case, the figs and the wasps

Reply: As in our reply to a related question above, the suitability of using JANE in the way we have done, for comparisons among different genomes of the same species, is confirmed both by the original paper introducing the software and the official website of JANE. Meanwhile, as explained above, in

response to your comments we did modify the terms used to describe concepts in these particular comparisons ("codivergence" and "associate switch", rather than "cospeciation" and "host switch").

-Full voucher information (including the herbarium where the specimen is deposited) need to be provided for each plant sequenced. In addition, the sequence data need to be deposited in a publically accessible archived, and ideally the phylogenies too (TREEbase or OpenTree, etc).

Reply: We added voucher information in Supplementary Table S1. The raw resequencing data and *Ficus microcarpa* reference genome produced for this project have been deposited in the Genome Sequence Archive BIG Data Center (http://bigd.big.ac.cn/gsa) under the accession numbers CRA001150 and GWHABKV00000000, respectively. Other results and related data used to generate the results of this study are attached as Supplementary Tables or Supplementary Source Data attached to the manuscript and Dryad (https://datadryad.org/stash/share/AyiHbELHaKUt9cfN1c1xk-kHk_ErG2U_iYavoiU-MLo). See the description of supplementary files for details.

Additional comments are included as tracked changes in the attached version of the manuscript.

Reply: Thanks very much for your careful checking and very helpful comments. Your main comments in the track-change version, and our replies, are shown in the following pages. We have already replied above to some questions in your track-change threads, and will not repeat our replies to these questions here.

##

Result part:

"Tetrad tree", This term isn't familiar to me – the authors are using it to say a tree inferred from a quartet-based algorithm, like ASTRAL or SVDquartets?

Reply: Yes, tetrad is a species tree inference tool within the ipyrad program (Eaton 2014) based on the SVDQuartets algorithm of Chifman and Kubatko (2014). It uses the theory of phylogenetic invariants to resolve quartet trees from SNPs for all sets of quartets in a larger tree, and then joins the quartets

together into a supertree using QMC. Refer to the ipyrad website for details (https://ipyrad.readthedocs.io/en/latest/API-analysis/cookbook-tetrad.html). However, in the revised version, **we have replaced "tetrad tree" with "Astral tree", a term that is more commonly used**. As we mention, the Astral topologies of *Ficus* phylogenetic reconstructions are strongly supported.

Chifman, J. & Kubatko, L. Quartet inference from SNP data under the coalescent model. *Bioinformatics* **30**, 3317-3324, doi:10.1093/bioinformatics/btu530 (2014).
Eaton, D. A. R. PyRAD: assembly of de novo RADseq loci for phylogenetic analyses. *Bioinformatics* **30**, 1844-1849, doi:10.1093/bioinformatics/btu121 (2014).

"**Complex genomic switch relationships among fig sections**"
I think this section should be removed, but if it retained, note that any conclusions derived from it are inferences, not observations (so events are inferred rather than detected or established)

Reply: Thanks for your suggestion. **We have removed this part**. Although this part supplied a nice visualization of the extent of breakdown of nuclear-organelle genome association, and thus supplies indirect evidence for hybridization among *Ficus* main lineages, a similar pattern could be inferred from the detailed results of phylogenetic reconciliation shown in the supplementary files (Supplementary Figs. 7-9).

In the revised version, we removed this part of the Results section. We added a new figure showing results of cophylogenetic reconciliation based on the *Ficus* Astral tree and chloroplast tree (Fig. 5A), which shows inferred genomic associate switches and associate losses among the three genomic compartments of *Ficus* plants (Fig. 4A). Meanwhile, full results of cophylogenetic reconciliation using all pairs of phylogenies based on nuclear and organelle genomic trees are supplied in Supplementary Figs. 7-9.The main conclusion (that hybridization has been frequent) was also further strengthened with direct evidence from ABBA-BABA *D*-statistics, BUCKy and PhyloNetworks (newly added in this version). All analyses of all data types (nuclear, mitochondrial, chloroplast) indicate frequent hybridization events during the evolutionary history of *Ficus*.

"Extensive cyto-nuclear phylogenetic discordance was consistently observed on a larger set of species,

including a published 200-species nuclear phylogeny based on five genes (5.5 Kb)[21] and the 59-species chloroplast genomic phylogeny[38] (Fig. 5, Supplementary Fig. 5). Similar to the pattern detected with the dataset including 15 species, the 59-species chloroplast genomic phylogeny also did not support the monophyly of any currently recognized subgenus or even of most sections, in contrast to the nuclear phylogeny (Fig. 1, Fig. 5)"

This section, comparing the present findings with those of earlier papers, belongs in the Discussion, rather than in the Results

Reply: **We have removed this part from the Results section**, and placed it in the Discussion, as requested.

Discussion part:

For original text: "The results provide a novel mechanism for explaining the high overall diversification of fig plants throughout much of their evolutionary history. The two co-evolutionary mechanisms (strict-sense and diffuse coevolution models) act synergistically, through bouts of reproductive isolation, where strict pollinator specificity leads to purification and specialization of genetic traits and ecological adaptation, punctuated by episodes of pollinator-switching-mediated hybridization, which can create novel genetic recombinants in figs thereby facilitating adaptation to environmental changes."

I appreciate the angle here, but I don't think a mechanism for diversification has been established. High hybridization rates, and/or high rates of pollinator switching, do not imply high rates of diversification to me. It's also unclear with considering figs to have a high diversification rate is itself justified—for a clade with a crown age of 75 million years to have an extant diversity of ~800 species seems unremarkable to me?

This sounds highly speculative to me. I don't see anything in the results of this paper that demonstrate these effects

I don't understand "co-evolutionary mechanisms (strict-sense and diffuse coevolution models)".

Reply: As we noted in our reply to your related general comments above, **we have deleted this part, removing virtually all discussion of potential codiversification mechanisms in fig-fig wasp**

mutualisms. In the revised version, we focus simply on explaining how hybridization and pollinator host-switching can occur so frequently in these supposedly specific obligate systems.

“Our results indicate that hybridization has played a substantial role in fig diversification throughout the evolutionary history of the genus.”

I don’t see the link with diversification. This would require specific analyses of diversification rates, and correlation of those rates with the factor of interest (in this case hybridization events).

Reply: Once again, **we have deleted this part.**

“This observed pattern is reasonable as sympatric taxa have a higher probability of hybridization via pollinator sharing or host-switches.”

But the inferred hybridization events are deep in the phylogeny, among ancestral taxa that likely had very different ranges? (The taxon sampling is sparse in this analysis, so those ancestral lineages are probably ancestral to broad suites of extant taxa?)

Reply: Thanks for this question. **We have removed this part.** In the revised version, we consider the implications of both Eurasian and Gondwanan origins of *Ficus*, both of which suggest ancestral distributions of most fig clades that are compatible with their coexistence for periods sufficient for sympatric hybridization events to occur. This long period of ancient sympatry makes it easier to understand the inferred hybridization events between lineages that are currently allopatric. Further, we emphasize what data are needed to distinguish between alternative answers to what we consider the currently unresolved question of site and time of origin of *Ficus*.

“Fourth, the 15 species in this study are, largely, quite distantly related; they represent 14 large species-rich clades (sections) within *Ficus*, among which the apparent divergence times range from 70 to 20 million years ago[21]. Thus, the likelihood of two species possessing the same set of alleles purely through ILS should be very low.”

There hasn’t been any description of the actual sequences yet—evidence of deep hybridization (or ILS), as the authors state, is different from extant species sharing the same sequence. Was that observed

(identical sequences shared among species)? If so, I agree that effectively rules out ILS (and deep hybridization), and makes me worried about contamination or sequencing errors.

Reply: **We have removed this part, as we do not reject the existence of ILS during evolution.** The three methods we used, ABBA-BABA D-statistics, BUCKy and PhyloNetworks, simply enable us to determine whether ILS alone can account for discordances within the nuclear genome, or whether some other process must also be invoked. What the three methods demonstrate is thus the necessity of invoking hybridizations; no method rejects the existence of ILS. Meanwhile, as we explained in response to your general comments above, given our procedures for data quality control during the sampling, sequencing and data clearing process, the probability that contamination or sequencing errors has affected our inference of hybridization is very low.

"Extensive hybridization and introgression are also supported indirectly by the difficult taxonomic delineation and phylogenetic inference in Ficus,"

I disagree. Or, at least, the evidence is so indirect as to not be worth mentioning (there are many ways to have challenging phylogenetic or taxonomic problems without there being any hybridization)

Reply: **We deleted this paragraph.**

"Thus, pollinator switches may be the primary mechanism underlying ancient hybridizations and introgressions."

To me "pollinator switch" implies an obligate pollinator that switches from one fig to another, whereas the cited evidence here seems to be of the pollinators not showing particularly strong host fidelity in the first place – the system seems to be "leaky".

Reply: **Yes, we agree with your point that the system seems to be "leaky", and we highlight this throughout the revised version. Also, the sentence in question was deleted in the revised version, as part of many changes in the Discussion section.** One of our main results is demonstrating that specificity in the so-called "specific obligate" pollination systems is not complete. It is thus important that BOTH reproductive isolation mediated by pollinator specificity AND hybridization mediated by

pollinator host-switches be considered in studying codiversification in such systems. We cite studies offering examples of both pollinator switches and pollinator sharing. Pollinator sharing may be the first step toward a definitive pollinator switch. An increasing number of studies have reported pollinator switches or pollinator sharing. Thus, although the fig-fig wasp system may be “the most extreme obligate pollination system known” (Cruaud et al. 2012), it also appears to be quite leaky on both ecological and evolutionary timescales, as previous studies, and now this one, respectively demonstrate. The quotation marks we put around “obligate” in describing this system are meant to highlight this leakiness,

Cruaud, A. *et al.* An extreme case of plant-insect co-diversification: figs and fig-pollinating wasps. *Syst. Biol.* **61**, 1029-1047, doi:10.1093/sysbio/sys068 (2012).

Explanations for apparently allopatric hybridization events

“Firstly, there may be evidence of the ghost of hybridization past[1,4], involving either extinct taxa, or extant taxa that were not sampled.”

But the hybridization events inferred are all old ones? Among the ancestors of extant lineages, not among extant species themselves? To rephrase – is it appropriate to refer to this inferred hybridization as “allopatric” in the first place when it is unclear where the hybridizing species (which existed a long time ago) occurred?

Reply: Thanks for these questions. **We have deleted the whole subsection titled** “**Explanations for apparently allopatric hybridization events**”. In this revision, we consider the implications of both Eurasian and Gondwanan origins of *Ficus*, both of which suggest ancestral distributions of most fig clades that are compatible with their coexistence for periods sufficient for sympatric hybridization events to occur. This long period of ancient sympatry makes it easier to understand the inferred hybridization events between lineages that are currently allopatric. Further, we emphasize what data are needed to distinguish between what we consider the currently unresolved question of site and time of origin of *Ficus*.

“Secondly, intercontinental hybridization may have occurred via the long-distance dispersal (LDD) of figs and/or fig wasps.”

This feels speculative, and unnecessary – as I mentioned above, I’m not sure why such explanations would be required given the uncertainty in the geographic range of the individuals that were the ones that actually hybridized

Reply: Thanks for this suggestion. **We agree and have deleted this part.**

Explanations for discordance between chloroplast and mitochondrial genomes

“Firstly, repeated bidirectional hybridization and introgression may lead to the reassortment of chloroplast and mitochondrial genomes in the cytoplasm via random genomic capture of one or both genomes[50]”

How would this work? The coalescent histories of the organelles should be identical – I don’t how the plastid and mitochondrion could fix on different copies (or even how different copies of the plastid vs the mitochondrion could be present in an individual organism – each individual inherits the maternal copy of both, together).

Reply: Thanks for these questions. **These sentences have disappeared, as part of the substantial changes in the Discussion section. However, the revised version does include responses to your questions.** We point out that biparental inheritance and heteroplasmy (i.e., multiple different copies of organellar genomes within the same cell or individual) of both chloroplast and mitochondrial genomes are more common in angiosperms than previously thought (Ramsey and Mandel 2019, Hu et al. 2008, Zhang et al. 2003). The pertinent part of the revised version was added in lines 274-286.

Ramsey, A. J. & Mandel, J. R. When one genome is not enough: organellar heteroplasmy in plants. *Annual Plant Reviews online* **2**, 619-658 (2019), doi: 10.1002/9781119312994.apr0616

Hu, Y. C., Zhang, Q., Rao, G. Y. & Sodmergen. Occurrence of plastids in the sperm cells of Caprifoliaceae: Biparental plastid inheritance in angiosperms is unilaterally derived from maternal inheritance. *Plant Cell Physiol.* **49**, 958-968, doi:10.1093/pcp/pcn069 (2008).

Zhang, Q., Liu, Y. & Sodmergen. Examination of the cytoplasmic DNA in male reproductive cells to

determine the potential for cytoplasmic inheritance in 295 angiosperm species. *Plant Cell Physiol.* **44**, 941-951, doi:DOI 10.1093/pcp/pcg121 (2003).

Potential mechanism of co-diversification in the fig-wasp mutualism

“Based on our results, we suggest that both the relatively stable reproductive isolation driven by the fidelity of pollinating fig wasps to their host figs, and adaptive introgression of genetic elements through interspecific hybridization following pollinator switches, have acted synergistically to generate the stunning diversity found in both partners of this coevolutionary relationship[16].”

Both parts of this statement seem conjectural to me—I don’t see how either “stable reproductive isolation” or “adaptive introgression” are demonstrated by the results of this study

I don’t think this has been established, either—what is stunning about the diversity of figs or fig wasps? (stunning diversit)

Reply: Once again, **we have removed this whole part**.

“These dynamics are most clearly demonstrated by the concept of the syngameon[54], in which a suite of closely related plant species maintain partial inter-fertility and exchange genetic material during episodic changes in environment and ecology.”

This is a very interesting idea! But I don’t think this mechanism is demonstrated by the current study

Reply: **We have removed this whole part.**

“our findings demonstrate that the general idea of pollinator-mediated hybridization in this system must be more seriously considered.”

It sounds like hybridizastion (which is pollinator-mediated by definition, since this is an animal-pollinated plant) is already well-documented and seriously considered in Ficus (multiple studies in the works cited report it)

Reply: Thanks for this comment. **We have removed this sentence, but the idea is retained elsewhere in the revised version.** It is true that multiple studies have demonstrated hybridization in

fig-fig wasp systems. However, most of these concern cases of current hybridization, under artificial conditions or in certain particular ecological situations. Most importantly, most of these studies are based on inferences from only a relatively few genes. Our study is to our knowledge the first that has applied a comprehensive sampling design combining deep sampling of both nuclear and cytoplasmic genomes across distantly related *Ficus* taxa. Finally, previous studies have lacked rigorous tests to distinguish true genetic introgression from the alternative process of incomplete lineage sorting. To avoid potential misunderstanding, we added a few sentences in the Introduction to highlight this distinctive feature of our study (lines 90-96).

###

Reviewer #3 (Remarks to the Author):

Overview:

The authors use complete genome sequences of representatives of major Ficus lineages to demonstrate that introgression and potentially hybrid speciation has occurred frequently within the group. This complex history of introgression is likely associated with pollinator host switching and ultimately diversification of the genus. Patterns of introgression were identified with deviations from equal minor quartet frequencies among gene trees and genome-wide ABBA-BABA tests. Strong cyto-nuclear discordance was also observed between chloroplast/mitochondrial and nuclear genomes. Cophylogeny analyses suggests that co-speciation alone is not sufficient, and that some degree of host-switching is necessary, which is coincident with the fig and fig-wasp cophylogeny. A speciation mechanism for Ficus is proposed with applications to other plant-pollinator associations too.

Major Comments:

Normally I do not belabor phylogenetic methods, but because the cophylogeny analyses are highly dependent on the chloroplast and mitochondrial trees, I would like to see that the results are robust to phylogenetic estimation method. Specifically, I find the use of invariants (i.e. tetrad) for the chloroplast and mitochondrial trees to be an odd choice- organellar genomes are single loci and I would thus argue treating them as unlinked SNP data for an MSC method inappropriate. Additionally, although invariant methods are consistent, they can require large numbers of SNPS to perform well for some phylogenetic problems such as large population sizes (e.g. Washer and Kubatko 2019). The

organellar genomes would be best treated with concatenated and partitioned ML. If there is some reference and annotated chloroplast and mitochondrial genomes, I would think extracting potential partitions (i.e. coding vs noncoding) from the gff would be straightforward.

Reply: Thanks very much for these insightful and helpful comments. **We have accepted your suggestions and produced ML trees of the two organellar genomes based on the concatenated full assembled sequences**. Chloroplast genomes of all 17 species (15 *Ficus* spp. and the two outgroup species), and mitochondrial genomes of 12 *Ficus* and 2 outgroup species were assembled into circular sequences. The three remaining fig species were assembled at the contigs level with GetOrganelle toolkit (Jin et al. 2018, refer to Supplementary Table 2 for details). We further inferred the ML trees of chloroplast and mitochondrial genomes based on the concatenated full genomic sequences with RAxML and FastTree (approximately-maximum-likelihood method, with local support values at nodes) (Price et al. 2010), respectively. Both trees had high support values at most nodes (Supplementary Fig. 4).

The concatenated sequence-based ML trees showed very similar topology to that of the invariants-based tetrad trees that were presented in the original submission, while the support values were greatly improved (see the tetrad trees and ML trees in the figure presented below). Responding to your remark about the weakness of methods of phylogenetic inference based on invariants, we deleted the results for all tetrad trees of these genomes, and only kept the ML trees based on concatenated sequences (Supplementary Fig. 5; Source Data Appendix 3).

To confirm the results of cophylogenetic analyses among nuclear, chloroplast and mitochondrial phylogenies, we repeated the comparison of the two main species trees of *Ficus* (Astral tree and primary concordance tree) based on nuclear data with organelle genome phylogenies, using this time the new ML trees of *Ficus* organellar genomes. Once again, frequent events of associate switch or associate loss among the three genomic compartments were repeatedly inferred in all analyses (Supplementary Table 8).

Jin, J.-J. *et al.* GetOrganelle: a simple and fast pipeline for de novo assembly of a complete circular chloroplast genome using genome skimming data. doi:10.1101/256479 %J bioRxiv (2018).

Price, M. N., Dehal, P. S. & Arkin, A. P. FastTree 2-approximately maximum-likelihood trees for large

alignments. *PLoS One* **5**, doi:10.1371/journal.pone.0009490 (2010).

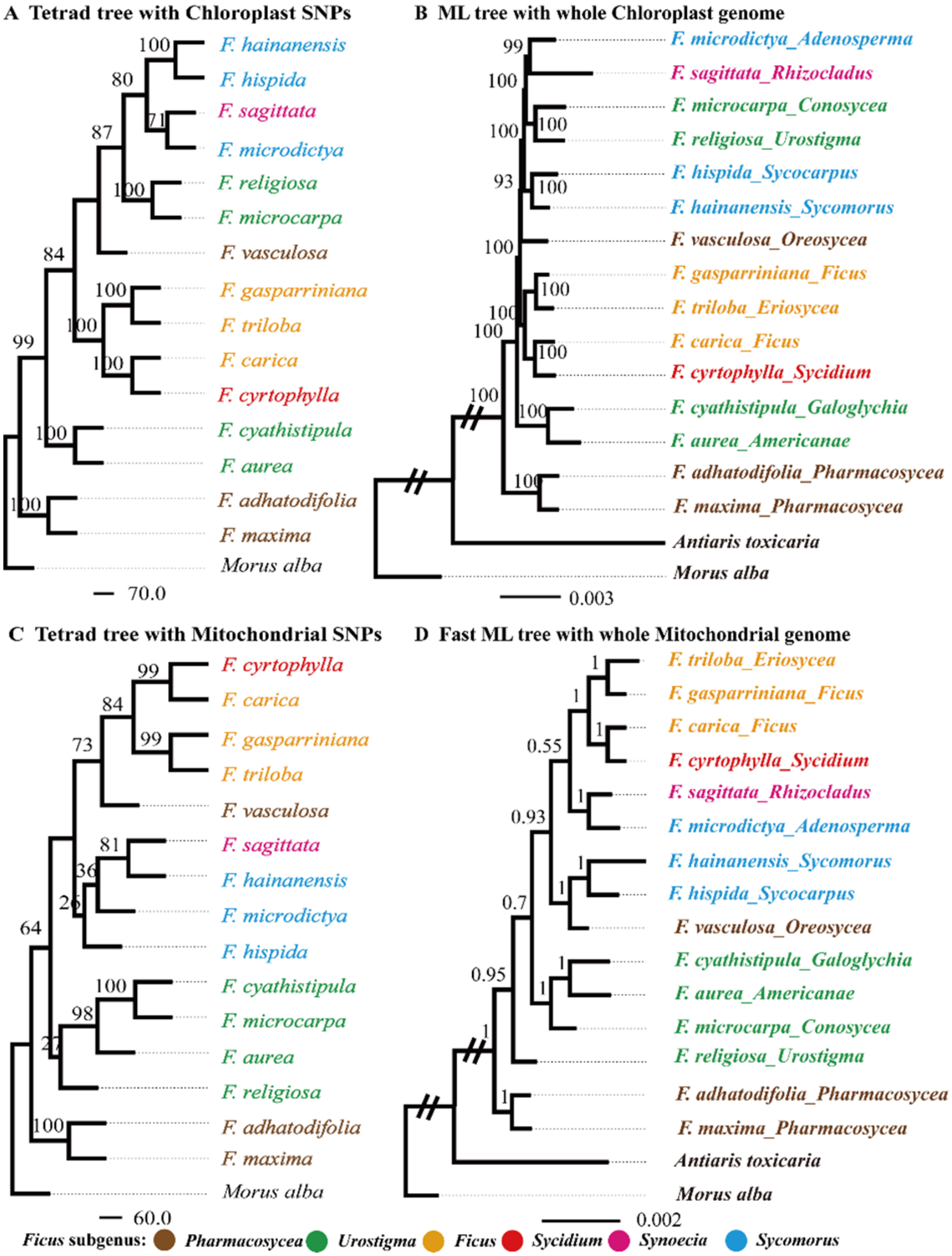

(The concatenated sequence-based ML trees of two organellar genomes show very similar topology

with that of the invariants-based tetrad tree in first submitted version, while the support values were greatly improved. Only ML trees were kept in the revised version.)

I have concerns about how loci are delimited for the BUCKy analyses. The loci are windows of the nuclear genome with 1,100 SNPS each – these must span large physical distances, right? I might be mistaken, but there is no information on the reference genome and how these sliding windows are oriented physically to judge otherwise. I would expect recombination has occurred with some frequency within these large genomic regions and treating them as independent loci for BUCKy and the introgression tests strikes me as though it will introduce some systematic biases. Why not use MDL (Ané 2011), or sliding windows of a fixed length (e.g. Figueiró et al. 2017)? Either way, I would like some justification on why treating large concatenated genomic regions as independent loci here would not be misleading – that a relatively small proportion of sites in each window is dominating the posterior distribution of topologies (e.g. Shen et al. 2017).

Reply: Thanks very much for these very helpful comments and suggestions. In the revised version, **following your suggestion, most analyses related to this aspect were reconducted based on three types of new added size-fixed windows datasets, and we also identified the physical location of each window in chromosomes of the reference genome (Supplementary Source Data Table S1) and supplied detailed information on the reference genome in Supplementary Note 1**. Analyses based on the SNPs-fixed windows were also maintained.

To recheck the species tree inferred with BUCKy, in the revised version we added new results of Astral species trees inferred based on the three types of size-fixed sliding windows (windows of size 500kb, 100kb and 50kb). Results of BUCKy analysis based on the SNPs-fixed large windows (Windows1000SNPs) were kept. We agree that the genome windows including 1,100 SNPs that we used in the original submission do span large physical distances in the genome (mean window size is ~1 Mb, with a range of 438 Kb-4.16 Mb). The 1000SNP windows-based Astral tree is similar to each of the three Astral trees based on the size-fixed windows datasets (Supplementary Fig.1). We agree that the risk of a disproportionate influence of a small proportion of sites in each window on phylogeny inference (e.g. Shen et al. 2017) may be higher with longer windows, but the new added

results with the shorter-window datasets support the same basic conclusions and suggest that this risk is not serious in our case.

Let us explain why we chose this kind of windows strategy. One reason we chose to fix the SNPs number of each window, instead of fixing the real window size, was to balance the number of SNPs in each window to ensure that each genomic window contains enough phylogenetic information to allow inference of the gene trees. Second, we chose a long window size to reduce the number of windows used for downstream analysis. More windows require more computational resources. We also conducted PhyloNetworks analyses, following the BUCKy analysis, before the initial submission, but we were unable to finish these analyses with the limited computational resources available at that time. We agree with your comment that longer windows will potentially suffer a greater influence of genomic recombination on inference of phylogeny (e.g. Shen et al. 2017). Furthermore, longer windows may also prevent detection of some very ancient hybridization events, as the introgressive genomic blocks in ancient hybridizations are expected to continually shorten over time due to genomic recombination (Ungerer et al. 1998; Buerkle and Rieseberg 2008). Some hybridization signals could thus be covered by derived signals if long genomic windows are used. Based on your comments and on the above considerations, we persevered and found more computational resources, enabling us to re-run the analysis based on four types of windows (three size-fixed and one SNPs-fixed windows). Again, these analyses using shorter windows support the same basic conclusions.

In detail, in the revised version, following the data type used in the paper you suggested (Figueiro et al. 2017), we constructed three non-overlapping sliding windows with fixed length (500kb, 100kb and 50 kb, respectively). Meanwhile, we also kept the long SNPs-fixed windows (1000SNPs windows). The physical distribution of each window of the four types in chromosomes of the reference genome is shown in Source Data Table S1. We inferred the Astral species trees based on each of the four types of windows. All showed similar topologies with highly supported values (Supplementary Fig. 1). PhyloNetworks analysis based on the 1000SNP windows inferred two hybridization events, and PhyloNetworks based on the 500kb windows inferred five additional hybridization events with greater involvement of deep nodes (Fig.2, Supplementary Fig. 6). All six hybridization events within *Ficus* clades were corroborated with ABBA-BABA *D*-statistics.

Figueiro, H. V. *et al.* Genome-wide signatures of complex introgression and adaptive evolution in the big cats. *Sci. Adv.* **3**, e1700299, doi:10.1126/sciadv.1700299 (2017).

Ungerer, M. C., Baird, S. J. E., Pan, J. & Rieseberg, L. H. Rapid hybrid speciation in wild sunflowers. *Proceedings of the National Academy of Sciences* **95**, 11757-11762, doi:10.1073/pnas.95.20.11757 (1998).

Buerkle, C. A. & Rieseberg, L. H. The rate of genome stabilization in homoploid hybrid species. *Evolution* **62**, 266-275, doi:10.1111/j.1558-5646.2007.00267.x (2008).

Shen, X. X., Hittinger, C. T. & Rokas, A. Contentious relationships in phylogenomic studies can be driven by a handful of genes. *Nat. Ecol. Evol.* **1**, doi: 10.1038/s41559-017-0126 (2017).

The divergence time estimation as described on lines 490-494 is strange. The population tree from BUCKy estimates branch lengths in coalescent units (Number of Generations/2Ne). r8s estimates absolute substitution rates and divergence times from a tree that has branch lengths in expected substitutions per site. I do not see how using coalescent units for estimating divergence times is a reasonable approximation. Why not just re-estimate branch lengths with ML and then use r8s (The penalized likelihood function used in R here)? I can appreciate that the divergence time estimation is not a central component of the results here, but normally you would want to determine a good rate smoothing parameter heuristically with cross-validation too.

Reply: Thanks very much for this helpful suggestion. **We have deleted the result in question, and re-estimated divergence times with a more formal analysis using the MCMCTREE program in PAML program (Yang 2007)**. The results of the new analysis are shown in Supplementary Fig. 2, and details of the method are presented in lines 514-531.

Yang, Z. H. PAML 4: Phylogenetic analysis by maximum likelihood. *Mol. Biol. Evol.* **24**, 1586-1591, doi:10.1093/molbev/msm088 (2007).

It is difficult to determine where the BUCKy tests for over-representation of a minor quartet and D-stats agree based on Figure 2. Also, but from what I can tell, the F. vasculosa ¬– F. sagittata introgression event from BUCKy in Fig. 2a seems absent from the D-stats in Fig. 2b. Although, the

D-stat result is quite strong based on Supplementary Table S6. Similarly, I do not see introgression between the common ancestor of subgenus Sycomorus and F. carica in Fig. 2a represented by the D-stats in Fig. 2b. In short, I think Fig. 2a and Fig 2b can be reconciled into a single figure with links that show events detected by both BUCKy and D-stats and those detected with D-stats alone.

Reply: Thanks very much for your comments and helpful suggestions. **In the revised version, we first rechecked all hybridization events and replotted the figures to allow easier comparison between hybridization results based on BUCKy and those based on the other two methods (ABBA-BABA *D*-statistics and PhyloNetworks). The two cases you mentioned (introgression between *F. vasculosa* and *F. sagittata*, and that between the common ancestor of subgenus *Sycomorus* and *F. carica*) were added to Fig. 2 of the revised version.**

There are 12 hybridization events detected with BUCKy following the topology of the primary concordance tree (new Fig. 2b, Supplementary Table 6); all those events are shown in the new Fig. 2b (on the primary concordance tree), and all those events are also corroborated by results of the ABBA-BABA *D*-statistics (Supplementary Table 6). In the new Fig. 2a, we show the main hybridization events detected with the ABBA-BABA *D*-statistics and PhyloNetworks. All six hybridization events between *Ficus* clades detected with PhyloNetworks are corroborated by the results of the ABBA-BABA *D*-statistics, and are plotted in Fig. 2a (on the Astral tree). However, there are still many hybridization events detected with the ABBA-BABA *D*-statistics that are not plotted in Fig. 2a (Fig. 1a in the previous version). We indicate these results with a dashed line crossing all the tips of the Astral tree in Fig. 2a, to balance information and ease of visualization. We show only the main hybridization events involving deeper nodes and those events within subgenera. The full results of hybridization events based on the ABBA-BABA *D*-statistics are supplied in Supplementary Table S6 and Source Data Table S2.

Meanwhile, following your suggestion, we combined hybridizations inferred with both PhyloNetworks and *D*-statistics into a single Astral tree (Fig. 2a). However, we kept hybridization events based on results of BUCKy analysis in the primary concordance tree separately (Fig. 2b). We did not combine all hybridization events detected with the three methods for display on the same phylogeny due to the phylogenetic conflict between the primary concordance tree and the Astral trees.

The Jane analyses are critical to the argument that co-phylogeny alone is not a good model for fig and fig-wasp coevolution and connecting host switching to introgression among fig species. But, I am also not convinced that some of the inferred costs for co-phylogeny between nuclear and organellar genomes is not due to poor phylogenetic estimation. Jane should be able to do two permutation tests to check that the cost scores and host switching events would not be expected by chance alone – There is a random tip-mapping procedure as well as a test for when the parasite (i.e. cp, mt, or pollinator) tree is random. I would like to see that the inferred costs and host switches would not be expected by chance alone.

Reply: Thanks for your helpful suggestion. In the revised version, **we re-ran Jane analyses with new inferred highly-supported phylogenies, under the optimal cost setting as you suggested.** We first selected the optimal cost setting by running both random tip mapping and random parasite tree permutation tests, with the cost range of each evolutionary event varying from 0 to 1 (excepting that of associate switch events, which varied from 0 to 2). The genetic algorithm parameters were set following a previous study (Cruaud et al. 2012), with generation number = 40, population size = 1,000, and sample size = 100. The optimal cost settings, with significant P-value from random setting and lowest observed cost value, were chosen for formal cophylogenetic analysis. Results of permutation tests pertaining to these analyses are shown in Supplementary Table 8.

Regarding the potential influence of poor phylogenies, first, as we already mentioned in a reply to a previous comment, two highly supported ML trees based on full genome sequences of both chloroplast and mitochondrial genomes were reconstructed and used for analysis. Furthermore, two alternative nuclear trees (Astral species trees, which were highly supported and consistently inferred based on four types of genomic windows, and the primary concordance tree [PCT tree], which showed a different topology at the basal node, with relatively weak support) were tested. Notably, whether the Astral tree or the PCT tree was used, the hypothesis of strict codivergence was clearly rejected, and consistently high frequencies of associate switch and loss events were inferred (Fig. 4a, Supplementary Table 8).

Meanwhile, we also strengthened our results on inference of pollinator host switches. We tested two alternative published pollinator phylogenies (Supplementary Fig. 5) (Cruaud et al. 2012, Machado et al. 2001), both considered plausible, against each of the different *Ficus* phylogenies based

on nuclear, chloroplast and mitochondrial genomes. Regardless of which of these two wasp phylogenies was tested against the various nuclear and organelle phylogenies, pollinator host-switch events always were inferred at higher frequency than were co-speciation events (Fig. 4, Supplementary Table 8). Although currently available pollinator phylogenies are not as robust as the fig phylogenies, no currently supported wasp phylogeny corresponds to any fig phylogeny based on nuclear, chloroplast, or mitochondrial data. This observation strongly suggests that host switching on the part of the associated pollinator wasp species, even between distantly related host taxa, has occurred on a regular basis (see Supplementary Notes 3-4 for details).

Machado, C. A., Jousselin, E., Kjellberg, F., Compton, S. G. & Herre, E. A. Phylogenetic relationships, historical biogeography and character evolution of fig-pollinating wasps. *Proc. R. Soc. B-Biol. Sci.* **268**, 685-694, doi : 10.1098/rspb.2000.1418 (2001).

Cruaud, A. *et al.* An extreme case of plant-insect co-diversification: figs and fig-pollinating wasps. *Syst. Biol.* **61**, 1029-1047, doi:10.1093/sysbio/sys068 (2012).

Minor Comments:

The comparison of the larger 5-gene nuclear phylogeny and 59 cp genome phylogeny on Fig. 5 and lines 198-209 is difficult to interpret. I really cannot follow any claim of cp-nuclear discordance given Figure 5. Why not parse out only the taxa represented in the cp tree from the nuclear dataset? That would make the comparison of the two trees easier for the reader. In general, I do not think this section adds much to the results because it does not do draw new insights from the data compared to (Cruaud et al. 2012) unless the results can be better integrated with the 15 taxon analyses.

Reply: Thanks for your suggestion. In response to your suggestion and a similar one of reviewer 2, **we deleted the comparison of this 5-gene nuclear phylogeny with the cp genome.** In the revised version, we kept only the published 59-species chloroplast genomic phylogeny along with the geographic distribution of each species, to show a general geographic pattern of chloroplast phylogeny (Supplementary Fig. 10). This figure now illustrates well our argument for chloroplast genome exchange via hybridization among sympatric species.

It is not clear why the 5-gene nuclear phylogeny is “improved” if the methods and data were the same. The re-analysis brings Urostigma together as a monophyletic group with 100% bootstrap support, but this would be due to the constraint imposed by the 15-taxon backbone tree (F. religiosa, F. aurea, and F. cyathistipula). Thus, the tree is practically forced to have 100% bootstrap support for Urostigma. I am guessing than an ML tree was re-estimated using GTR-CAT with RAxML and the 15 taxon species tree was used as a constraint. Please elaborate and clarify in the methods. Cruaud et al. (2012) used several approaches for phylogenetic estimation and citing “same procedures” on line 579 is ambiguous.

Reply: Thanks for your comments. In the revised version, **we have removed the 5-gene nuclear phylogeny and the related cyto-nuclear comparison using this 5-gene nuclear phylogeny.** Yes, for the “improved” 5-gene nuclear phylogeny, the methods and data are exactly those presented in the original publication (Cruaud et la. 2012), the only difference being that we added a backbone tree of 15 fig species based on whole nuclear genomes inferred in this study.

As a complementary analysis to the quartet-based tests with BUCKy, consider passing the quartet concordance factors to PhyloNetworks (Solis-Lemus et al. 2017). Estimating an explicit network might help resolve some of the ambiguity in where introgression occurred on the phylogeny. I think this would require minimal effort given the quartet concordance factors are already estimated. The authors allude to this on lines 374-382 of the discussion anyway.

Reply：Thanks very much for your suggestion. **As we reply above, we did the PhyloNetworks analysis based on results of both BUCKy analysis and Astral trees** (Fig. 2a, Supplementary Fig. 6). All six hybridization events among *Ficus* clades inferred by PhyloNetworks were corroborated by results of ABBA-BABA *D*-statistics.

I do not see the fig reference genome cited anywhere. Is it Mori et al. (2017).

Reply：The fig reference genome used here is not the published *Ficus carica* genome (Mori et al. 2017). **We de novo sequenced *Ficus microcarpa* as reference genome with higher quality.**

Detailed information is added in Supplementary Note 1.

Are all of the Ficus species sequenced here diploids? Are any of the species potentially allopolyploids?

Reply：**We assessed the ploidy for each *Ficus* genome using the program nQuire** (Weiß et al. 2018), which is a statistical framework for ploidy. **This result show that all *Ficus* sampled in this study are diploids and none is allopolyploid** (Source Data Table S4).

Weiß, C. L., Pais, M., Cano, L. M., Kamoun, S. & Burbano, H. A. nQuire: a statistical framework for ploidy estimation using next generation sequencing. *BMC Bioinformatics* **19**, 122, doi:10.1186/s12859-018-2128-z (2018).

References:

Ané C. 2011. Detecting phylogentic breakpoints and discordance from genome-wide alignments for species tree reconstruction. Genome Biol Evol. 3:246-258.

Cruaud A, Rønsted N, Chantarasuwan B, Chou LS, Clement WL, Couloux A, Cousins B, Genson G, Harrison RD, Hanson PE, et al. 2012. An extreme case of plant-insect codiversification: figs and fig-pollinating wasps. Syst Biol. 61:1029-1047.

Figueiró HV, Li G, Trindade FJ, Assis J, Pais F, Fernandes G, Santos SHD, Huhes GM, Komissarov A, Antunes A, et al. 2017. Genome-wide signatures of complex introgression and adaptive evolution in the big cats. Sci Adv. 3:e1700299. doi: https://doi.org/10.1126/sciadv.1700299.

Mori K, Shirasawa K, Nogata H, Hirata C, Tashiro K, Habu T, Kim S, Himeno S, Kuhara S, Ikegami H. 2017. Identification of RAN1 orthologue associated with sex determination through whole genome sequencing analysis in fig (Ficus carica L.). Sci Rep. 7:41124. Doi: https://doi.org/10.1038/srep41124.

Solis-Lemus C, Bastide P, Ané C. 2017. PhyloNetowks: A package for phylogenetic networks. Mol Biol Evol. 34:3292-3298.

Shen X-X, Hittinger CT, Rokas A. 2017. Contentious relationships in phylogenomic studies can be driven by a handful of genes. Nat Ecol Evol. 1:0126. doi: https://doi.org/10.1038/s41559-017-0126.

Washer M, Kubatko L. 2019. Consistency of SVDQuartets and maximum likelihood for coalescent based species tree estimation. bioRxiv. doi: https://doi.org/10.1101/523050.

Reply：Thanks very much for the very helpful additional references you supplied. This study has benefited from consultation of these studies.

Reviewer #1 (Remarks to the Author):

I was pleased to see that the authors have added new analyses to address the questions of whether hybridization events in Ficus correspond to periods of past sympatry and to host switching events in the pollinators.

I am slightly disappointed that these analyses are not more prominently highlighted, mostly being buried in supplemental methods and results, though I supposed these were indeed 'supplemental' in that I suggested them after the fact. I understand why the authors chose not to make much of the biogeographic analysis; the sampling of taxa is too think and the geographic distribution of the major clades to wide to draw terribly meaningful conclusions. I was surprised, however, that the authors did make more of the fact that inferred hybridization events in figs correspond to host switching events by the pollinators. To me this is a quite interesting result, but I suppose that is a matter for the authors to decide.

As a purely organizational issue, I will note that I had to struggle quite a bit to see where the authors had made changes to the manuscript in order to address specific reviewer points. In their response to reviews the authors reference portions of the manuscript that have been revised, for example they say 'Text related to these new analyses was added in the Methods, Results and Discussion sections of the revised manuscript. '

However, the text in the corresponding sections often references specific issues brought up in review only very obliquely, and because the authors did not indicate the corresponding page or line numbers it was not always clear what text had been added.

As a frequent reviewer and journal editor, I would suggest more clearly indicating exactly where changes have been made or text added. Without that the reviewer has to search for changes, leading to crankiness and delayed reviews.

Reviewer #2 (Remarks to the Author):

I thank the authors for their thoughtful consideration of my comments (and those of the other reviewers)! Unfortunately, I still have some significant reservations. I'll outline those somewhat broadly, and then list some more specific comments/suggestions by line number (I apologize for some repetition in my comments).

First, I am being harder on this paper than I might otherwise be, because of the visibility of this journal. In addition, the authors include many analyses (some of which they might not necessarily be that familiar with), so there are many areas where mistakes can be made. This is a drawback of an ambitious project like this one. Reviewer 1 says that this paper applies the accepted tools of the field, and does so appropriately. As I am not familiar with the norms of the cospeciation field, I will defer to their assessment, however, as the authors themselves say, "results of cophylogenetic analyses are highly dependent on the phylogenetic topology" and I am concerned that the phylogenetic components of this paper are not robust. Specifically, the narrative of this paper depends upon incongruence among phylogenetic inferences indicating hybridization, and I don't think that conclusion was adequately supported. The authors do a good job of demonstrating that ILS is not a sufficient explanation for the incongruence, but they don't rigorously investigate the slew of potential non-biological causes (most notably, a host of possible estimate errors, contamination, alignment inference errors, hidden paralogy, systematic error due to model mis-specification, etc). For one specific example, ASTRAL attributes all incongruence to ILS (as the authors point out), so if hybridization/introgression are common, as this manuscript reports, than ASTRAL will be unreliable (and so will any method that assume a bifurcating tree).

I appreciate the clarification of the steps taken to ensure that there weren't indexing errors, etc., in the sequencing pipeline (including the example of the samples sequenced at different facilities). What were the steps taken to ensure that there was no cross-contamination during the extraction through to library prep steps? I note that the authors did not directly respond to my question of whether there are any sequences that are shared among accessions (if there are shared sequences that suggests contamination or very recent gene flow).

I am glad that the quartet-based analyses were removed for the organellar datasets (that was a good point of reviewer3), but am disconcerted by the differences between those earlier (quartet-based) and current phylogenies. In the mito data, for example, F.cyathistipula went from 100% support as sister to F.microcarpa to 100% support as sister to F. aurea? I disagree that these qualify as "very similar topologies." If whole-genome data are available, for a tree of this size, I would expect the topologies to be identical. To me, this suggests that there should be more attention paid to possible difficulties in the phylogenetic inference.

In comparing the rates of co-speciation with the rates of pollinator-switching, keep in mind that this is a very sparsely sampled fig phylogeny, so on an given branch there are presumably many (unconsidered) speciation events. It only takes one of those events to be a broad pollinator-switch for that signal to be detectable, whereas the co-speciation events are undetectable.

45 I still feel that this statement is excessively conjectural (at least for the abstract). I don't think that this paper demonstrates that Ficus has an unusually high diversity to begin with (for a clade that's 75 million years old to have 800 species does not seem exceptional), or demonstrates that diversification dynamics have been influenced by hybridization or host-switching. I think the idea is interesting, just conjectural.

53 Maybe include some supporting citations from the polyploidy literature here

78 Minor quibble, but I wouldn't use the phrase "morphologically defined clades." Clades are monophyletic groups, so they are defined by their shared evolutionary history. One might infer that a group is a clade based on its morphology, but morphology does not define a clade.

83 Also extremely minor quibble, but I'm not sure that our genetic information has gotten more sophisticated (or what that would mean, really). It's just that we have more of it.

88 This sentence seems to say that there is evidence of discordance that is consistent with discordance? (Am I misreading this?).

92 Basing an inference of this sort on relatively few genes is not necessarily a weakness. Are there more specific limitations of these studies? (E.g., were they not able to get strong support for backbone nodes, etc?)

106 "The inference of frequent hybridization, among even distantly related lineages of figs, is further supported by the significant lack of concordance of the topologies of the chloroplast and mitochondrial phylogenies of the 15 Ficus species with any supported nuclear phylogenies, or, indeed, with each other" – what is the other evidence, which this evidence "further" supports?

129 As was raised by an earlier review, the application of ASTRAL to these sorts of arbitrary chunks is problematic (there's no reason to think that each chunk represents a single non-recombining region, and thus no reason to think that the gene tree from that chunk will be correct). Since ASTRAL is very sensitive to genetree accuracy, this is potentially a significant problem. Some other method that does not require gene trees, like SVDquartets, would be more appropriate.

135 An extant group cannot be basal to another extant group (the correct term for this relationship in

this case is "sister")

147 "Monophyletic clade" is redundant (if a group is not monophyletic, it is not a clade)

151 As I expand on a little in comments about fig. 1 (below), the geographic reconstructions make me nervous given the sparseness of the taxon sampling. I don't know enough about fig phylogeny to assess this concern, but for these inferences to be reliable, the taxon sample has to accurately represent the current range of the species of the genus (if major transitions are missing, that's a problem). Since most of the included samples are Eurasian, I'm not surprised that much of the ancestry of the tree is reconstructed as Eurasian, but that doesn't really tell us much (in my opinion). Also, existence in "eurasia" does not mean that the species were sympatric. How many of the currently "Eurasian" species are truly sympatric?

161 Misused "basal"

167 I don't think this result—highly supported trees—is at all notable when whole-genome data are used (rather, I would expect such support).

169 This result (the incongruence between the plastid and mito genome inferences) still seems like a warning sign to me. While I conceded that the authors' explanation (in the response document), invoking biparental inheritance and heteroplasmy, is possible, it feels like a significant leap to attribute these results to such processes. I would want, at a minimum, some evidence that these processes occur at appreciable rates, and preferably some evidence that they do so in Ficus. Otherwise, there are a whole host of other explanations for this incongruency, including lots of opportunities for inference errors. A search of the literature for intra-linkage incongruence will reveal other cases.

175 I would argue that the contrasting results from the two analysis approaches indicates a need for further analytical attention, rather than for taking one result over the other.

177 Applying the same method four times and getting high support each time is not a strong indication of the reliability of the conclusion (the fact that the data were treated differently each time helps, but those differences are relatively small).

186 "four-taxon"

191 How is was this conclusion ("at least one hybridization event") drawn? ABBA-BABA tests provide a measure of the statistical support for introgression, rather than a count of events, right?

201 I am uncomfortable with the results from each of these data-clustering approaches (window size) being treated as independent tests. These are not independent—if a result is found using one window size, and a different result is found using another window size, that means that neither result is reliable, rather than that both are reliable.

207 But it is not just biological processes that need to be considered here. Stochastic variation in the evolutionary process, model mis-specifications, labwork errors, etc., can all lead to discordance.

209 BUCKy doesn't find hybridization events—need some other wording here to describe the conclusions drawn from the BUCKy inferences

211 Basal misused

215 A more compelling treatment here, in my opinion, is to only report and discuss the inferences that are supported by ALL the analytical approaches applied. That would also make the results/discussion easier to follow.

221 What does failure to diverge mean in this context?

222 The JANE analyses attribute all incongruence to either “associate switches” or “associate losses”? What do these mean (especially “associate losses”) in the context of genomic compartments within organisms? And there are many reasons for inter-compartmental incongruence that don’t fall into either of those two categories.

241 Why are the two wasp phylogenies substantially different? As with the differences observed in this study among different analyseis approaches and different datatype, this incongruence makes me nervous. If you just did a codiversification analysis of one wasp phylogeny on the other, would you find strong evidence of “host-switching” within the wasps?

268 This line is critical—if the methods are not identifying the same events, than I don’t think their conclusions are reliable.

270 Demonstrating the ILS is not a sufficient explanation for the discordance does not mean that there has to be hybridization—this second part does not follow from the first (sorry to keep harping on this, but it keeps coming up).

426 As I said this in my earlier review, all sampled individuals should be represented by a herbarium voucher (and that voucher information provided in this manuscript). I consider this to be essential.

518 I don’t follow how the MCMCtree analyses were done, but I haven’t used this program myself. The final MCMCtree analyses used fixed substitution rates derived from the earlier PAML runs?

544 Some model-selection and partitioning would be appropriate for the ML analyses of the concatenated organellor genomes. FastTree seems like a poor choice (from an accuracy perspective) unless slower methods were not able to run on this dataset.

562 A quibble, but I would say that a significant ABBA BABA result indicates that ILS is not sufficient to explain the result, but does not necessarily indicate gene flow

637 Basal misused

Fig1 – The response to reviews states that Antiaris is more closely related to Figus than Morus is, yet, here Antiaris and Morus are sister species—this conflict needs to be resolved. The tip states are presumably that of the tip species (so F.carica, for example, is Eurasian). But given the sparseness of the taxon sample, are there a lot of excluded range-change events? E.g., are all members of subgenus Sycidium Eurasian? If range-changes are missing, than the rates of range change will be underestimated in the model, and the reconstructions will be unreliable. This problem would be even worse if the included ranges are biased (e.g., if a disproportionately high number of the included species are Eurasian). The scale on this figure is a little awkward – why not make the units “millions of years” and have the first labeled interval be “25”, instead of making the units “100 million years” and having the first label be 0.25? The nodes on this tree that were calibrated in the divergence-time estimation should be indicated.

Fig2 – The subfigure labeling (A and B) should be switched. Some of the inferred events here are impossible, because they occur between an ancestor and its descendent. For example, the green line that connects the ancestral lineage of Sycomorus and the GTS clade with the F.gasparriniana branch. This further highlights to me that these incongruence analyses are picking up things other than signals of hybridization. B: again, BUCKy can’t “detect” hybridization events—you can infer hybridization events from BUCKy output, but that’s a somewhat different matter. As with figure A, to be biologically possible, both participants in a hybridization have to be contemporaneous, and I’m not sure that is

true of all the inferred events on this tree (I don't see any ancestor-descendent pairs, but it’s still not clear that the branches can be rescaled to allow all those events to have contemporaneous participants. If it is possible, it would require pretty dramatic resizing of branches, such that the divergence time estimates would have to have been wildly off).

Fig3 In cases where sister species could be rotated to avoid crossed lines, that would probably be more appropriate here (otherwise the crossed lines give the impression of incongruence where none exists). So in fig D the two figs with Tetrapus pollinators could be rotated to show that that’s a congruent relationship, and same for the bottom branch in fig C.

Fig4 I’m not sure what these different events would refer to, especially in the comparisons of the different genomic compartments, but regardless, it looks like no Duplication or Fail Div. events occur. If these events are not realistic for this system, they should be removed from the model to avoid artifacts.

Reviewer #3 (Remarks to the Author):

Overview:
I reviewed a previous version of this manuscript and commend the authors for the addition of many new analyses and changes to the manuscript text that have simplified the overall message. I thank the authors for the additional supplementary notes and appendices that have addressed previous questions as well. My comments are thus limited to these additional or edited sections of text.

Major Comments:
I find discussion section “Mechanisms of hybridization: signaling and pollinator recognition of hosts” a bit disconnected from the rest of the paper. Although the speciation mechanisms outlined here seem reasonable, none of the results in the manuscript are referenced here. Are the citations regarding different profiles of volatile compounds among Ficus species and their associated pollinator species relevant to the lineages sampled here? In short, is there a way to connect any of this past research, especially those regarding when wasps can oviposit in distantly related species, to any of the introgression events detected here. If not, lines 345-353 in the second paragraph seem repetitive and vague and can probably be cut down. Some specific examples could also help make this section less vague to those who are not experts in the fig-fig wasp system.

Minor Comments:

Lines 88-90: “evidence suggesting potential discordance” … “that is consistent with potential discordance”. This sounds very weak when it looks like the evidence is quite good.

Lines 222-225: The authors state there are three events when in Table S8 there are 5 events used for computing the cost scores. This is clear in the methods on lines 616-617, but not clear here. The distinction between cospeciation/codivergence events and other event types can probably be more clearly explained here, and how those non-cospeciation events could be relevant to introgression within host phylogeny.

Lines 255-258: I do not think this is a failure of the data or should be perceived as a negative message at all. Introgression between the early diverging lineages (e.g. Urostigma or Pharmacosycea) and the more recently diverged ones was clearly demonstrated. We should expect these types of introgression events to mislead the species tree estimation (see Jiao et al. 2020), especially given the short internal branch lengths of the Astral trees in Supplementary Figure S1 relative to the Ficus MRCA height and the inferred gamma parameter is around 0.2 between F. cyanthistipula and F. triloba from PhyloNetworks in Supplementary Figure S6. The conflict in species tree relationships across methods

will probably persist even with additional taxon sampling because of the difficulty of estimating a single binary species tree with both short internode distances between speciation events (high ILS) and the high levels of introgression between non-sister taxa.

Line 517: Is there a citation for this recommendation?

Line 521: Cite dos Reis et al. (2011) for the approximate likelihood method.

Line 539: The use of "Supplementary Table" in both the Supplementary Information and Appendices is a bit confusing. I was looking for the BioGeoBears model comparison table in the SI until I realized the meaning was that it was in the excel file in the appendix. Maybe some different naming for tables in the appendices is needed. Also, both the BioGeoBears and nQuire tables are rather simple and could be included in the SI pdf rather than in the excel spreadsheet in the Appendix.

Line 575: Delete this sentence rather than say there are results but not show them.

Line 595 and throughout: There are several abbreviations that are defined multiple times or in some cases spelled out. These are BCA, CF, PCT. Please check these are defined at first use and be consistent with the abbreviations throughout.

Figure 5B: Do the pollinator switch events coincide or make sense in the context of inferred introgression events? This does not need to be addressed in the figure legend, but the pollinator switching events beg some discussion in the section "Cophylogenetic analyses and detection of associate switches using cytoplasmic genomes and pollinator phylogenies".

It is probably not appropriate to include Illumina's proprietary documents regarding index switching in the Appendices. The text in the manuscript is sufficient.

References:

Jiao X, Flouri T, Rannala B, Yang Z. 2020. The impact of cross-species gene flow on species tree estimation. Syst Biol. doi: https://doi.org/10.1093/sysbio/syaa001.

dos Reis M, Yang Z. 2011. Approximate likelihood calculation on a phylogeny for Bayesian estimation of divergence times. Mol Biol Evol. 28:2161-2172.

##

Point-by-point reply to reviewers' comments

##

Reviewer #1 (Remarks to the Author):

I was pleased to see that the authors have added new analyses to address the questions of whether hybridization events in Ficus correspond to periods of past sympatry and to host switching events in the pollinators.

I am slightly disappointed that these analyses are not more prominently highlighted, mostly being buried in supplemental methods and results, though I supposed these were indeed 'supplemental' in that I suggested them after the fact. I understand why the authors chose not to make much of the biogeographic analysis; the sampling of taxa is too thin and the geographic distribution of the major clades to wide to draw terribly meaningful conclusions. I was surprised, however, that the authors did make more of the fact that inferred hybridization events in figs correspond to host switching events by the pollinators. To me this is a quite interesting result, but I suppose that is a matter for the authors to decide.

Reply: Thanks very much for your appreciation of our manuscript and for your very helpful suggestion. The new results on *Ficus* biogeographic history and comparison of estimated hybridization and host-switch events, based on your previous suggestion, have greatly improved our discussion of ancient hybridization.

As a purely organizational issue, I will note that I had to struggle quite a bit to see where the authors had made changes to the manuscript in order to address specific reviewer points. In their response to reviews the authors reference portions of the manuscript that have been revised, for example they say 'Text related to these new analyses was added in the Methods, Results and Discussion sections of the revised manuscript. '

However, the text in the corresponding sections often references specific issues brought up in review only very obliquely, and because the authors did not indicate the corresponding page or line numbers it was not always clear what text had been added.

As a frequent reviewer and journal editor, I would suggest more clearly indicating exactly where

changes have been made or text added. Without that the reviewer has to search for changes, leading to crankiness and delayed reviews.

Reply: We apologize for the difficulty you experienced in trying to follow what changes we made. In fact, the text was so extensively revised, following the reviewers' comments, that showing all track changes would have resulted in a very unwieldy document. In this revision, we have clearly indicated all changes from the last submitted version using different coloured (blue) text, and the line numbers associated with each change are indicated in our responses to comments.

##

Reviewer #2 (Remarks to the Author):

I thank the authors for their thoughtful consideration of my comments (and those of the other reviewers)! Unfortunately, I still have some significant reservations. I'll outline those somewhat broadly, and then list some more specific comments/suggestions by line number (I apologize for some repetition in my comments).

Reply: Thanks for your critical and helpful comments, which have improved our manuscript. We have made many further modifications based on your comments.

First, I am being harder on this paper than I might otherwise be, because of the visibility of this journal. In addition, the authors include many analyses (some of which they might not necessarily be that familiar with), so there are many areas where mistakes can be made. This is a drawback of an ambitious project like this one. Reviewer 1 says that this paper applies the accepted tools of the field, and does so appropriately. As I am not familiar with the norms of the cospeciation field, I will defer to their assessment, however, as the authors themselves say, "results of cophylogenetic analyses are highly dependent on the phylogenetic topology" and I am concerned that the phylogenetic components of this paper are not robust. Specifically, the narrative of this paper depends upon incongruence among phylogenetic inferences indicating hybridization, and I don't think that conclusion was adequately supported. The authors do a good job of demonstrating that ILS is not a sufficient explanation for the incongruence, but they don't rigorously investigate the slew of potential non-biological causes (most notably, a host of possible estimate errors,

contamination, alignment inference errors, hidden paralogy, systematic error due to model mis-specification, etc). For one specific example, ASTRAL attributes all incongruence to ILS (as the authors point out), so if hybridization/introgression are common, as this manuscript reports, than ASTRAL will be unreliable (and so will any method that assume a bifurcating tree).

Reply: We recognize that bold hypotheses are held to a higher standard of proof. This was our motivation for deploying such a diverse battery of analyses, all of which support our central hypotheses that hybridization resulting from host-switching of the pollinators as well as biparental (and independent) inheritance of mitochondrial and chloroplast genomes have occurred frequently throughout the evolutionary history of *Ficus*. These events are preserved and revealed in the complete *Ficus* genomes. Here, we reply in more detail to each of your specific comments below.

We first address your concern that the phylogenetic results of the study are “not robust”, which we believe is not justified. First, the Astral *Ficus* tree is based on the re-sequenced whole genome data, which we use in many subsequent analyses. It is the most robust *Ficus* phylogeny available, not only because of its consistently high support values at almost all nodes as a bifurcating tree (Supplementary Fig. 1A-D), but also because it is supported by the main tree of the *Ficus* phylogenetic network inferred with PhyloNetworks, which does not assume bifurcating relationships, as do Astral tree reconstructions (Supplementary Fig. 6) (See Results part, lines 138-143 and lines 183-189). Thus, our analyses of the reconstruction of *Ficus* evolution and our cophylogenetic analyses based on the Astral *Ficus* tree are robust. Second, we are well aware that there is disagreement about topology between published phylogenies of both figs and fig wasps. Thus, we also considered the alternative *Ficus* phylogeny generated with a Bayesian approach (e.g., primary concordance tree [PCT tree] inferred with BUCKy), for all analyses that were conducted with the Astral tree (See Results part, lines 183-189). Most important, what we point out is that (i) **neither** of the two principal published phylogenies for fig wasps are congruent with **any** of the phylogenies (based on nuclear {Astral OR PCT tree}, chloroplast, or mitochondrial data {estimated using maximum likelihood approach}) for figs (Fig. 4), and (ii) all the pollinator host-switching events inferred through the cophylogenetic analyses were matched to hybridization events detected between different host fig groups (Supplementary Table 9). One can always claim that more data are needed to make phylogenies more robust. Because the datasets consist of complete genomic information for nuclear and organelles, we suggest instead that **no phylogeny**

of figs based on strict bifurcating branching will ever be more robust, because there has been so much reticulate evolution, resulting from hybridization mediated by host-switching. The phylogeny of figs, we believe, is better represented by a web than by a bifurcating tree. Our hypothesis suggests why.

Again, we emphasize that the most powerful evidence for the conclusion of frequent hybridization in this study comes from results of the ABBA-BABA *D*-statistics. This method is not affected by the phylogenetic topology (see methods section, lines 569-583), and its robustness has been widely tested (Green et al. 2010, Durand et al. 2011, Martin et al. 2013, Árnason and Halldórsdóttir 2019). We highlighted this information in discussion part (lines 274-282) as follow: "Frequent hybridization events were inferred to have taken place within and across all six *Ficus* subgenera, based on three different types of analyses applied to nuclear genome data: ABBA-BABA *D*-statistics[46,47], PhyloNetworks[45], and BUCKy[43,44] (Fig. 2, Supplementary Fig. 7, Supplementary Table 10). In particular, ABBA-BABA *D*-statistics[46-48] suggest extensive hybridization events in all 15 sampled species (14 sections) (see Supplementary Table 6)."

Concerning potential non-biological sources of error, we described the stringent measures we took to exclude potential sources of error that we list here and present in greater detail in reply to specific questions posed by the reviewer: 1) our taxonomic background and expertise on this system enabled us to avoid fig species misidentification (see details in methods section, lines 443-454). 2) We avoid sequencing contamination via the dual-indexed Illumina markers and standard sequencing pipeline (see details in methods section, lines 459-465). 3) We avoid alignment errors and the use of hidden paralogs by using a high-quality reference genome (Supplementary Note 1). 4) Our strict data quality control pipeline enabled us to greatly reduce the risk of potential unknown systematic error (see details in methods section, lines 466-509). 5) we conducted multiple hybridization detection analyses based on multiple datasets, all described in the manuscript. 6) Furthermore, during this cycle of revision, we collected Nanopore or PacBio sequencing genomic data of nine species (eight *Ficus* and one outgroup), in which DNA samples were independently extracted, sequenced, and analysed. By showing the similar "interspecies-genomic-similarity value" based on this new dataset and the Illumina dataset used in the manuscript, we further rejected the risk of cross contamination, meanwhile further confirmed the conclusion of frequent hybridization (see our detailed reply to your next comments, lines 138-

248). The results all support the same pattern of frequent hybridization and pollinator host-switches (lines 190-257). The data quality and our procedures for avoiding errors are clearly described. The major conclusions derived from analyses of these data are all clearly supported. Perhaps most importantly, as we point out, our major conclusions generate a series of testable hypotheses, and our data will be available upon publication to all who wish to examine them.

Green, R. E. *et al.* A draft sequence of the neandertal genome. *Science* **328**, 710-722, doi:10.1126/science.1188021 (2010).

Durand, E. Y., Patterson, N., Reich, D. & Slatkin, M. Testing for ancient admixture between closely related populations. *Mol. Biol. Evol.* **28**, 2239-2252, doi:10.1093/molbev/msr048 (2011).

Martin, S. H. *et al.* Genome-wide evidence for speciation with gene flow in Heliconius butterflies. *Genome Res.* **23**, 1817-1828, doi:10.1101/gr.159426.113 (2013).

Árnason, E. & Halldórsdóttir, K. Codweb: Whole-genome sequencing uncovers extensive reticulations fueling adaptation among Atlantic, Arctic, and Pacific gadids. *Sci. Adv.* **5**, eaat8788, doi:10.1126/sciadv.aat8788 (2019).

I appreciate the clarification of the steps taken to ensure that there weren't indexing errors, etc., in the sequencing pipeline (including the example of the samples sequenced at different facilities). What were the steps taken to ensure that there was no cross-contamination during the extraction through to library prep steps? I note that the authors did not directly respond to my question of whether there are any sequences that are shared among accessions (if there are shared sequences that suggests contamination or very recent gene flow).

Reply: The dual-indexed libraries strategy with unique dual-indexes for each sample ensures that our sequencing data are not influenced by the potential risk of both index-hopping and cross-contamination during the entire sequencing process. Any sequences with a different dual-index are removed from the samples during the data separation process.

We further clarified the paragraph related to this point (lines 459-465): "To avoid potential DNA contamination during sequencing, such as that via index-swapping, we constructed dual-indexed libraries with unique indexes for each sample. The dual indexes contained a total of 16 base pairs (bp) and were inserted into the flanking regions of the target DNA fragments. Raw

reads that had any mismatch with index sequences were clustered as undetermined sequences and removed from our analysis. Meanwhile, the unique dual-index of each sample also ensured unambiguous separation of the raw sequenced data among *Ficus* species, and avoidance of potential sequence contamination among samples.” We note that the use of dual-index marker is the standard and well-accepted approach to minimize contamination issues with Illumina sequencing. But in further response to Reviewer 2’s concerns we took additional, time-consuming steps.

In order to corroborate that there is NO evidence of DNA or sequence contamination, we conducted independent Nanopore sequencing for seven species (six of which correspond to species included in the current study) and additionally collected recent published PacBio sequencing data for two other fig species (Table reply 1). For the nine species, the sampling, DNA extraction, library construction and sequencing were conducted independently, there was thus no chance for DNA contamination among them. With the independently sequenced dataset of these nine species (eight fig species, one outgroup), and the Illumina sequencing dataset of related species or sections used in our manuscript, we compare patterns of genetic similarity based on the Illumina data or on these with Nanopore-PacBio sequencing methods. The new results show that the inter-taxa genomic similarities based on the current Illumina sequencing dataset are generally similar with those based on the independently sequenced Nanopore-PacBio dataset (Table reply 2). The results strongly support lack of DNA contamination among samples of our current dataset. Detailed methods and results are shown as follows:

Table reply 1. Species sequenced with Illumina technology and Nanopore or PacBio technology. Names in red indicate different species but belonging to the same section. The new Nanopore or PacBio sequenced data come from the first author’s team with Nanopore technology or from recently published data using PacBio technology.

Species (Illumina)	Species (Nanopore-PacBio)	Subgenus	Section	Source of Nanopore-PacBio data
Antiaris toxicaria	*Antiaris toxicaria*	outgroup	outgroup	Authors unpublished (Nanopore)
F. maxima	*F. maxima*	*Pharmacosycea*	*Pharmacosycea*	Authors unpublished (Nanopore)
F. vasculosa	*F. callosa*	*Pharmacosycea*	*Oreosycea*	Authors unpublished (Nanopore)
F. microcarpa	*F. microcarpa*	*Urostigma*	*Conosycea*	Authors unpublished (Nanopore)
F. religiosa	*F. religiosa*	*Urostigma*	*Urostigma*	Authors unpublished (Nanopore)
F. hispida	*F. hispida*	*Sycomorus*	*Sycocarpus*	Authors unpublished (Nanopore)
F. gasparriniana	*F. erecta*	*Ficus*	*Ficus*	Shirasawa et al. 2020 (PacBio)
F. carica	*F. carica*	*Ficus*	*F_carica calde*	Usai et al.2020 (PacBio)
F. cyrtophylla	*F. tinctoria*	*Sycidium*	*Sycidium*	Authors unpublished (Nanopore)

Reply Table 2. Section-level inter-taxa genomic similarity based on Illumina data (left-down) and Nanopore-PacBio independently sequenced data (right-up) of nine Moraceae species. Species samples with red names indicate that different species of the same section were sampled in the two datasets. The colour within the table cells reflects the value within them, with dark green indicate lowest value (3.47) and dark red indicate highest value (34.77).

Samples	*Antiaris toxicaria*	*F. maxima*	*F. callosa*	*F. microcarpa*	*F. religiosa*	*F. hispida*	*F. erecta*	*F. carica*	*F. tinctoria*
Antiaris toxicaria		4.96	5.71	4.78	5.03	5.33	5.55	5.57	5.03
F. maxima	3.71		25.77	19.71	20.69	21.69	24.6	24.52	20.57
F. vasculosa	3.61	21.21		29.18	30.74	29.67	33.7	33.57	30.88
F. microcarpa	3.38	15.1	18.88		25.45	24.3	27.59	27.35	23
F. religiosa	3.95	19.56	25.79	20.68		25.3	29	28.93	24.68
F. hispida	4.28	19.3	24.69	19.6	23.82		30.74	30.06	27.68
F. gasparriniana	3.47	20.02	27.72	17.6	24.08	25.05		34.73	32.34
F. carica	3.65	20.54	28.17	18.19	24.9	25.64	29.04		34.77
F. cyrtophylla	4.02	21.33	28.6	20.2	26.16	28.11	29.58	33	

One can compare the inter-taxa genomic similarities based on different genomic sequencing methods, to check whether there is evidence of cross-contamination among samples that may have arisen during the whole process, as DNA contamination can generate higher genomic similarity. Eight *Ficus* genomes and one outgroup were *de novo* sequenced independently (DNA extraction and library construction were conducted independently for each sample) and assembled using long reads by either Nanopore (six fig taxa and *Antiaris toxicaria*, from our new sequence data) or PacBio (two fig species, Shirasawa et al. 2020, Usai et al. 2020) (Table reply 1). We compared the inter-taxa genomic similarity among the nine species based on both datasets. The nine species are representatives of eight section-level fig taxa and one outgroup, with six species being identical between the two datasets. The inter-taxa genomic similarity compared at section level between the two datasets does not support DNA contamination as an explanation.

Detailed method: We developed a kmer-counting-based method to investigate the genomic similarity between any two *Ficus* species. For our current Illumina sequencing samples, the Illumina short reads from each species were split into 27-bp fragments (i.e. 27-mers). Subsequently, non-redundant 27-mers were extracted from each species by merging the kmers with exactly the same sequences. We further identified shared and unique kmers between two samples and calculated the shared proportion to evaluate the sequence similarity using the following formula:

$$\text{Similarity} = S/\min(A, B),$$

where, S indicates shared non-redundant k-mers, A and B represent number of unique non-redundant kmers in sample A and sample B, respectively, and min (A, B) will extract the minimum value of A and B. For these genomes independently sequenced and assembled by either PacBio or Nanopore platforms, the 27-mers were extracted directly from the assembled genomes and the genomic similarities among the nine species were further calculated using the same formula aforementioned.

We show very similar inter-taxa genomic similarity for each species pair based on the two datasets (Table reply 2). In general, the range of inter-taxa genomic similarities of eight fig species is 15.1 – 33 for the Illumina dataset used in the current manuscript (Bottom left corner of Table reply 1), while the similarity value range is 19.71 – 34.77 for the independently sequenced Nanopore-PacBio dataset (top right corner of Table reply 2). Generally, for each species pair, the genomic similarity based on Illumina sequencing dataset is similar to or even slightly lower than that based on the independent sequenced Nanopore-PacBio dataset. This result is not consistent with the conjecture that the Illumina data we collected may have been affected by DNA cross-contamination.

We stress that this kind of additional cross-checking and use of independent sampling and analytical techniques has not previously been necessary for published empirical studies. The new Nanopore sequenced dataset was generated to provide additional evidence to Reviewer 2 and is presented in the reply letter. We plan to publish a separate study based on these results elsewhere. As all review comments and reply will be published online as supplementary files of manuscript published in the “Nature Communications”, all readers will be able to see current materials if they are interested in these points.

Usai, G. et al. Epigenetic patterns within the haplotype phased fig (*Ficus carica* L.) genome. Plant J., doi:10.1111/tpj.14635 (2020).

Shirasawa, K. *et al.* The *Ficus erecta* genome aids *Ceratocystis canker* resistance breeding in common fig (*F. carica*). *Plant J.* **102**, 1313-1322, doi:10.1111/tpj.14703 (2020).

Furthermore, analyses of the independent, Nanopore-PacBio dataset (which does not suffer cross-contamination or paralogy or misalignment, as the sampling, sequencing, de novo assembly and annotation were all conducted independently) using the same ABBA-BABA D-statistics, again detect frequent hybridization among all sampled fig species (Reply Table 3). The results further confirm our conclusion of frequent hybridization among all sampled *Ficus* clades in this manuscript.

Reply Table 3. Detailed results of four-taxon ABBA-BABA D-statistics tests for hybridization detected at different phylogenetic levels in *Ficus*, based on the newly collected Nanopore-PacBio sequencing dataset for nine species. Significant evidence of gene flows between taxa is highlighted with bold names. Z-values highlighted with green suggest significant gene flow between H2 and H3, while Z values highlighted with yellow indicate significant gene flow between H1 and H3. The same pattern was shown in Supplementary Table 6 based on the current Illumina dataset of 15 *Ficus* species.

H1	H2	H3	H4	nABBA	nBABA	D	JK-D	Z	pvalue
F. erecta	*F. hispida*	*F. carica*	*A. toxicaria*	178616.99	235848.57	-0.138	-0.138	-37.418	0.000
F. erecta	*F. hispida*	*F. microcarpa*	*A. toxicaria*	27430.97	28108.07	-0.012	-0.012	-2.150	0.032
F. erecta	*F. hispida*	*F. callosa*	*A. toxicaria*	161524.72	177911.76	-0.048	-0.048	-14.137	0.000
F. erecta	*F. hispida*	*F. maxima*	*A. toxicaria*	139175.68	159415.44	-0.068	-0.068	-20.417	0.000
F. erecta	*F. hispida*	*F. religiosa*	*A. toxicaria*	137366.35	153921.21	-0.057	-0.057	-21.630	0.000
F. erecta	*F. carica*	*F. hispida*	*A. toxicaria*	178616.99	204184.92	-0.067	-0.067	-18.600	0.000
F. erecta	*F. carica*	*F. microcarpa*	*A. toxicaria*	25587.16	25079.73	0.010	0.010	1.658	0.097
F. erecta	*F. carica*	*F. callosa*	*A. toxicaria*	164254.38	168312.09	-0.012	-0.012	-4.701	0.000
F. erecta	*F. carica*	*F. maxima*	*A. toxicaria*	143569.48	144622.14	-0.004	-0.004	-1.261	0.207
F. erecta	*F. carica*	*F. religiosa*	*A. toxicaria*	140687.39	143164.07	-0.009	-0.009	-3.149	0.002
F. erecta	*F. microcarpa*	*F. maxima*	*A. toxicaria*	59137.13	72463.01	-0.101	-0.101	-18.964	0.000
F. erecta	*F. callosa*	*F. microcarpa*	*A. toxicaria*	27983.23	33416.25	-0.088	-0.088	-12.023	0.000
F. erecta	*F. callosa*	*F. maxima*	*A. toxicaria*	157070.52	187369.14	-0.088	-0.088	-19.658	0.000
F. erecta	*F. callosa*	*F. religiosa*	*A. toxicaria*	164620.98	166898.14	-0.007	-0.007	-2.062	0.039
F. erecta	*F. maxima*	*F. microcarpa*	*A. toxicaria*	59137.16	25222.03	0.402	0.402	84.995	0.000
F. erecta	*F. maxima*	*F. callosa*	*A. toxicaria*	157070.52	219420.36	-0.166	-0.166	-37.526	0.000
F. erecta	*F. maxima*	*F. religiosa*	*A. toxicaria*	143095.84	175135.41	-0.101	-0.101	-31.293	0.000
F. erecta	*F. religiosa*	*F. maxima*	*A. toxicaria*	143095.84	271436.08	-0.310	-0.310	-65.468	0.000
F. erecta	*F. tinctoria*	*F. microcarpa*	*A. toxicaria*	28878.27	27535.96	0.024	0.024	4.460	0.000
F. erecta	*F. tinctoria*	*F. callosa*	*A. toxicaria*	163547.34	179518.06	-0.047	-0.047	-18.095	0.000
F. erecta	*F. tinctoria*	*F. maxima*	*A. toxicaria*	144484.09	155594.91	-0.037	-0.037	-13.395	0.000
F. erecta	*F. tinctoria*	*F. religiosa*	*A. toxicaria*	142906.23	153665.57	-0.036	-0.036	-11.913	0.000
F. hispida	*F. carica*	*F. microcarpa*	*A. toxicaria*	28654.92	27409.29	0.022	0.022	3.878	0.000
F. hispida	*F. carica*	*F. callosa*	*A. toxicaria*	176922.99	164102.95	0.038	0.038	10.093	0.000
F. hispida	*F. carica*	*F. maxima*	*A. toxicaria*	158881.69	139867.46	0.064	0.064	16.598	0.000
F. hispida	*F. carica*	*F. religiosa*	*A. toxicaria*	152929.88	138870.10	0.048	0.048	13.924	0.000
F. hispida	*F. microcarpa*	*F. maxima*	*A. toxicaria*	61405.22	69870.13	-0.064	-0.064	-12.308	0.000
F. hispida	*F. callosa*	*F. microcarpa*	*A. toxicaria*	29272.61	33954.29	-0.074	-0.074	-8.959	0.000
F. hispida	*F. callosa*	*F. maxima*	*A. toxicaria*	164948.16	173715.85	-0.026	-0.026	-5.358	0.000
F. hispida	*F. callosa*	*F. religiosa*	*A. toxicaria*	170486.48	155919.50	0.045	0.045	12.172	0.000
F. hispida	*F. maxima*	*F. microcarpa*	*A. toxicaria*	61405.22	26968.70	0.390	0.390	78.866	0.000
F. hispida	*F. maxima*	*F. callosa*	*A. toxicaria*	164948.16	209883.77	-0.120	-0.120	-23.831	0.000
F. hispida	*F. maxima*	*F. religiosa*	*A. toxicaria*	151642.97	167501.21	-0.050	-0.050	-13.955	0.000
F. hispida	*F. religiosa*	*F. maxima*	*A. toxicaria*	151642.97	255133.58	-0.254	-0.254	-54.613	0.000
F. hispida	*F. tinctoria*	*F. microcarpa*	*A. toxicaria*	31237.30	29341.55	0.031	0.031	6.002	0.000
F. hispida	*F. tinctoria*	*F. callosa*	*A. toxicaria*	172941.81	171932.15	0.003	0.003	0.835	0.404
F. hispida	*F. tinctoria*	*F. maxima*	*A. toxicaria*	157669.53	149387.56	0.027	0.027	8.141	0.000
F. hispida	*F. tinctoria*	*F. religiosa*	*A. toxicaria*	153371.94	147301.45	0.020	0.020	6.255	0.000
F. carica	*F. microcarpa*	*F. maxima*	*A. toxicaria*	57402.76	70684.61	-0.104	-0.104	-21.371	0.000
F. carica	*F. callosa*	*F. microcarpa*	*A. toxicaria*	27087.71	33189.60	-0.101	-0.101	-14.965	0.000
F. carica	*F. callosa*	*F. maxima*	*A. toxicaria*	154683.38	183654.95	-0.086	-0.086	-19.872	0.000
F. carica	*F. callosa*	*F. religiosa*	*A. toxicaria*	163360.18	163566.39	-0.001	-0.001	-0.209	0.834
F. carica	*F. religiosa*	*F. maxima*	*A. toxicaria*	141141.58	264895.10	-0.305	-0.305	-67.306	0.000
F. carica	*F. tinctoria*	*F. erecta*	*A. toxicaria*	176852.27	198723.89	-0.058	-0.058	-19.481	0.000
F. carica	*F. tinctoria*	*F. hispida*	*A. toxicaria*	167359.34	169518.10	-0.006	-0.006	-2.337	0.019
F. carica	*F. tinctoria*	*F. microcarpa*	*A. toxicaria*	25393.90	24534.41	0.017	0.017	2.863	0.004
F. carica	*F. tinctoria*	*F. callosa*	*A. toxicaria*	146192.73	157006.35	-0.036	-0.036	-13.966	0.000
F. carica	*F. tinctoria*	*F. maxima*	*A. toxicaria*	128325.36	137873.90	-0.036	-0.036	-13.627	0.000
F. carica	*F. tinctoria*	*F. religiosa*	*A. toxicaria*	129214.49	137560.61	-0.031	-0.031	-11.318	0.000
F. microcarp	*F. callosa*	*F. maxima*	*A. toxicaria*	71016.21	63943.10	0.052	0.052	9.700	0.000
F. microcarp	*F. religiosa*	*F. erecta*	*A. toxicaria*	81370.27	47480.57	0.263	0.263	44.063	0.000
F. microcarp	*F. religiosa*	*F. hispida*	*A. toxicaria*	78400.18	48198.64	0.239	0.239	43.115	0.000
F. microcarp	*F. religiosa*	*F. carica*	*A. toxicaria*	78538.68	47075.44	0.250	0.250	42.721	0.000
F. microcarp	*F. religiosa*	*F. callosa*	*A. toxicaria*	82434.98	43042.73	0.314	0.314	56.390	0.000
F. microcar	*F. religiosa*	*F. maxima*	*A. toxicaria*	65796.92	78086.36	-0.085	-0.085	-15.016	0.000
F. microcarp	*F. religiosa*	*F. tinctoria*	*A. toxicaria*	79607.86	49979.54	0.229	0.229	39.494	0.000
F. microcarp	*F. tinctoria*	*F. maxima*	*A. toxicaria*	71274.13	60365.00	0.083	0.083	17.779	0.000
F. callosa	*F. maxima*	*F. microcarpa*	*A. toxicaria*	63943.10	24259.44	0.450	0.450	76.708	0.000
F. callosa	*F. maxima*	*F. religiosa*	*A. toxicaria*	150001.90	180131.18	-0.091	-0.091	-31.313	0.000
F. callosa	*F. religiosa*	*F. maxima*	*A. toxicaria*	150001.90	250066.37	-0.250	-0.250	-59.333	0.000
F. callosa	*F. tinctoria*	*F. microcarpa*	*A. toxicaria*	35957.39	29261.66	0.103	0.103	13.651	0.000
F. callosa	*F. tinctoria*	*F. maxima*	*A. toxicaria*	181339.49	163431.22	0.052	0.052	11.702	0.000
F. callosa	*F. tinctoria*	*F. religiosa*	*A. toxicaria*	162364.35	170697.83	-0.025	-0.025	-8.261	0.000
F. religiosa	*F. tinctoria*	*F. maxima*	*A. toxicaria*	263570.27	149606.16	0.276	0.276	63.395	0.000

I am glad that the quartet-based analyses were removed for the organellar datasets (that was a good point of reviewer3), but am disconcerted by the differences between those earlier (quartet-based) and current phylogenies. In the mito data, for example, F.cyathistipula went from 100% support as sister to F.microcarpa to 100% support as sister to F. aurea? I disagree that these qualify as "very similar topologies." If whole-genome data are available, for a tree of this size, I would expect the topologies to be identical. To me, this suggests that there should be more attention paid to possible difficulties in the phylogenetic inference.

Reply: As requested by reviewer 3 (and endorsed by Reviewer 2), all tetrad trees were removed from our previous revision. However, the differences you note apply to species within the same subgenus (*Urostigma*), and mostly concern the mitochondrial genome, with chloroplast phylogenies being much more similar between the two methods. The chloroplast phylogenies shown by tetrad trees and ML trees are almost the same, except for the position of the *F. religiosa-F. microcarpa* clade. Furthermore, the bootstrap values of the tetrad chloroplast tree are generally high (99 or 100 at many nodes, 71 to 87 at others). However, they are still not as high as in the ML chloroplast tree, for which all nodes, except two, have bootstrap values of 100 (99 or 93 at the two other nodes).

In contrast, there are larger differences between the mitochondrial tetrad trees and ML trees. Although both mitochondrial phylogenies show section *Pharmacosycea* as sister to all other figs, and then subgenus *Urostigma* as sister to section *Oreosycea* and the gynodioecious figs, within each main clade there are many differences in topology. The relatively low bootstrap values at most nodes of the tetrad tree (values lower than 80 at five deep nodes) indicate that the tetrad tree does not perform well for inferring mitochondrial phylogeny. Therefore, as ML trees for both chloroplast and mitochondrial phylogenies have higher bootstrap values at most nodes than do tetrad trees (in the case of mitochondrial phylogenies, much higher values), we chose to use ML trees for both cytoplasmic-genome phylogenies, following reviewer 3's comments.

As Reviewer 3 noted in the preceding revision, although quartet-based tetrad methods are mainly consistent with ML methods, they typically require large numbers of SNPs to perform well for some phylogenetic problems (for example, when population sizes are very large (Wascher and Kubatko 2020, https://doi.org/10.1093/sysbio/syaa039 [Preprint:

https://www.biorxiv.org/content/biorxiv/early/2019/01/17/523050.full.pdf]). In this case, this is especially a concern for mitochondrial genomes given the low number of SNPs (4548 in average) observed in this genome (even smaller than for the chloroplast genome [5463 SNPs in average]) (Supplementary Table 2). Thus, we agree with the reviewers that the quartet-based tetrad methods used in the preceding version may not have been appropriate, and therefore we removed those analyses, and use only the trees from ML-based methods.

In comparing the rates of co-speciation with the rates of pollinator-switching, keep in mind that this is a very sparsely sampled fig phylogeny, so on any given branch there are presumably many (unconsidered) speciation events. It only takes one of those events to be a broad pollinator-switch for that signal to be detectable, whereas the co-speciation events are undetectable.

Reply: We agree that the accurate number of both cospeciation and host-switch events, as well as the real position where each event happened on the phylogeny, are influenced by the sampled species. More dense sampling (which we state must be an essential part of studies testing the hypotheses generated by this one) will be helpful for detecting more cospeciation and host-switching events, especially those within certain *Ficus* sections or clades. However, the guess that co-speciation may be underestimated with sparse samples does not fit results of the few available studies conducted with dense sampling of closely related species (Satler et al. 2019, Yang et al 2015, Renoult et al. 2009). Instead, they show a similar pattern of high levels of host switching and suggest that the underlying assumption that cospeciation is more common is incorrect. With 15 taxa representing the main sections of *Ficus* and their corresponding pollinator wasps, the cospeciation and host-switch events we inferred are mainly those that happened among main clades, e.g. at the section level. We didn't uncover the events (neither cospeciation nor host-switches) within those clades. We mainly discuss the pattern carefully at the section level, and when extending conclusions to finer taxonomic scales (e.g., within section), we simply cite previous studies at finer taxonomic levels that have found patterns of frequent host switches among closely related species using cophylogenetic analyses (Satler et al. 2019) or using phylogenetic incongruency patterns (Yang et al 2015, Renoult et al. 2009). The results from finer taxonomic scales do not support the idea that strict cospeciation is the default underlying model of co-diversification, but rather than host switches are common and probably more common than

cospeciation. Those results at finer phylogenetic scales thus support our conclusions at broader phylogenetic scales.

Here, a high frequency of pollinator host switches was consistently found no matter which fig phylogenies were used in the cophylogenetic analyses with either of the two wasp phylogenies (Fig. 4B-C, Supplementary Table 8). This inference is consistent with a recent study that found the same pattern of larger frequency of pollinator host switches in a sample of 11 closely related species from section *Americana* (Satler et al. 2019). So, although it would seem plausible that the sparse phylogenetic sampling could lead to an overestimation of the relative frequency of host switches relative to cospeciation (which would go undetected), the evidence from studies focused on closely related species suggests that host switches are indeed more prevalent than cospeciation (Yang et al 2015, Renoult et al. 2009, Satler et al. 2019). The previously unrecognized point that these data make is that hybridization events have occurred throughout the history of *Ficus* evolution and have left a clear record of reshuffling the major elements of the *Ficus* genome.

We also highlighted main above information in the Discussion part (Lines 323-332) as follow: “Importantly, the frequent pollinator host switches detected throughout *Ficus* evolution potentially provide the mechanism behind the pervasive hybridization. …… These results are also consistent with the inferred pollinator host-switching events and potential genetic exchange in studies of the relationships between closely related sympatric figs in Neotropical section *Americana*[33,34] and African section *Galoglychia*[32].”

Cruaud, A. *et al.* An extreme case of plant-insect co-diversification: figs and fig-pollinating wasps. *Syst. Biol.* **61**, 1029-1047, doi:10.1093/sysbio/sys068 (2012).

Renoult, J. P., Kjellberg, F., Grout, C., Santoni, S. & Khadari, B. Cyto-nuclear discordance in the phylogeny of *Ficus* section *Galoglychia* and host shifts in plant-pollinator associations. *BMC Evol. Biol.* **9**, 248, doi:10.1186/1471-2148-9-248 (2009).

Satler, J. D. *et al.* Inferring processes of coevolutionary diversification in a community of Panamanian strangler figs and associated pollinating wasps. *Evolution*, doi:10.1111/evo.13809 (2019).

Weiblen, G. D. How to be a fig wasp. *Annu. Rev. Entomol.* **47**, 299-330, doi:10.1146/annurev.ento.47.091201.145213 (2002).

Yang, L.-Y. *et al.* The incidence and pattern of co-pollinator diversification in dioecious and monoecious figs. *Evolution* **69**, 294-304, doi:10.1111/evo.12584 (2015).

45 I still feel that this statement is excessively conjectural (at least for the abstract). I don't think that this paper demonstrates that Ficus has an unusually high diversity to begin with (for a clade that's 75 million years old to have 800 species does not seem exceptional), or demonstrates that diversification dynamics have been influenced by hybridization or host-switching. I think the idea is interesting, just conjectural.

Reply: We have revised this statement. We suggest that the hybridization we inferred may help account for the diversity of *Ficus* species. We note that the number of *Ficus* species (about 800) is unusual for a woody genus (Frodin 2004), and the fact that it accounts for more than twice the number of species as all other 36 genera of Moraceae family combined (< 400 spp.; http://www.theplantlist.org/browse/A/Moraceae/) begs explanation. Our results suggest that explanations for the high diversification of *Ficus* (relative to other tropical trees and to Moraceae in general) need to incorporate hybridization. We modified the sentence at the end of the abstract (lines 43-47) as follows: "These findings are most consistent with frequent host-switching by the pollinators, leading to fig hybridization, even between distantly related clades. We suggest that these hybridization events are a dominant feature of fig/wasp coevolutionary history, and by generating novel genomic combinations in the figs have contributed to the remarkable diversity exhibited by this mutualism."

Frodin, D. G. History and concepts of big plant genera. *Taxon* **53**, 753-776, doi:10.2307/4135449 (2004).

53 Maybe include some supporting citations from the polyploidy literature here

Reply: We have done this. Polyploid and homoploid hybridization receive almost equal amounts of attention in the following two studies:

Mallet, J. Hybrid speciation. *Nature* **446**, 279-283, doi:10.1038/nature05706 (2007).

Abbott, R. *et al.* Hybridization and speciation. *J. Evol. Biol.* **26**, 229-246, doi:10.1111/j.1420-9101.2012.02599.x (2013).

78 Minor quibble, but I wouldn't use the phrase "morphologically defined clades." Clades are monophyletic groups, so they are defined by their shared evolutionary history. One might infer that a group is a clade based on its morphology, but morphology does not define a clade.

Reply: We replaced "morphologically defined clades" with "morphologically defined taxa". The related sentence was modified as follows: "Given the observation that morphologically defined taxa (subgenera and sections) of figs are usually pollinated by distinct genera of pollinators…"

83 Also extremely minor quibble, but I'm not sure that our genetic information has gotten more sophisticated (or what that would mean, really). It's just that we have more of it.

Reply: We think both are true. Yes, we have both more genetic information, as well as qualitatively different kinds of data that have also permitted the development of new methods more powerful than those that could have been envisaged previously. We modified the text (lines 80-82) to read "However, broader species sampling combined with increasingly detailed and comprehensive genetic analyses of both host and pollinator species have identified ever larger numbers of exceptions to the previously assumed extreme host specificity."

88 This sentence seems to say that there is evidence of discordance that is consistent with discordance? (Am I misreading this?).

Reply: Thanks. We apologize for not having been clearer. We are speaking of two DIFFERENT discordances: The "fig-fig wasp phylogenetic discordance" is consistent with the "nuclear-chloroplast phylogenetic discordance of figs". We have modified the sentence (lines 89-91) to read as follows: "Specifically, both the apparent discordance of both the fig-fig wasp phylogenetic relationships and the nuclear-chloroplast associations of figs across sections of figs is consistent with the possibility of hybridization at even these deeper taxonomic scales[15,20,32,38]."

92 Basing an inference of this sort on relatively few genes is not necessarily a weakness. Are there more specific limitations of these studies? (E.g., were they not able to get strong support for backbone nodes, etc?)

Reply: Yes, all previous *Ficus* phylogenies, which were based on a small number of genes, do

suffer from weak support for backbone nodes. We point out this weakness in the new version as follows (lines 94-98): “However, the phylogenetic inferences of much of the existing (suggestive) work are based on data from relatively few, marginally informative genes that only weakly support crucial basal nodes. Further, these studies lack both genetic depth (e.g., entire nuclear and organelle genomes) and a comprehensive sampling design (e.g., taxa that represent what are thought to be the major evolutionary transitions in the genus *Ficus*).”

106 “The inference of frequent hybridization, among even distantly related lineages of figs, is further supported by the significant lack of concordance of the topologies of the chloroplast and mitochondrial phylogenies of the 15 Ficus species with any supported nuclear phylogenies, or, indeed, with each other” – what is the other evidence, which this evidence “further” supports? Reply: We apologize for lack of clarity. The first sentence in the paragraph in question concerns the analyses based on nuclear genomes. The “further” on line 107 of the previous version refers to the additional evidence supplied by analysis of cytoplasmic genomes, which are not concordant with any supported nuclear phylogenies, nor with each other. We have modified the text to read as follows (Lines 111-1117): “We found direct evidence indicating frequent hybridization and introgression events pervasive in fig nuclear genomes. These events were detected even among distantly related, currently non-sympatric lineages of figs, suggesting a process that has been acting throughout the evolutionary history of figs. These inferences were corroborated by multiple analytical approaches (ABBA-BABA D-statistics, PhyloNetworks, and Bayesian Concordance). Furthermore, this inference is also supported by the significant lack of concordance of the topologies of the chloroplast and mitochondrial phylogenies of the 15 *Ficus* species with any supported nuclear phylogenies, or, indeed, with each other.”

129 As was raised by an earlier review, the application of ASTRAL to these sorts of arbitrary chunks is problematic (there’s no reason to think that each chunk represents a single non-recombining region, and thus no reason to think that the gene tree from that chunk will be correct). Since ASTRAL is very sensitive to gene tree accuracy, this is potentially a significant problem. Some other method that does not require gene trees, like SVDquartets, would be more appropriate. Reply: We presented the tetrad tree (same method used as for the SVDquartets tree) in our first

version. Generally, this method gives results consistent with those given by BUCKy, but we removed the results of tetrad trees owing to other potential weaknesses of this method (see Reviewer comments and our reply above, lines 249-284). The power of this method is also influenced by effective population size (Wascher and Kubatko 2019) which was hard to control in our study.

Concerning the potential weakness of the ASTRAL tree, we agree that Astral trees, or even all bifurcating trees based on available methods, including SVDquartets trees, are not perfect, as interspecies genetic exchange is increasingly proving to be a common pattern in many organisms (Taylor and Larson 2019). However, our results show that the negative influence of potential gene recombination within genomic windows on robustness of inferences from ASTRAL trees is not a serious problem in our study. We inferred Astral trees four times based on four datasets with genomic windows of different sizes, providing the opportunity to observe the effect of window size on our phylogenetic inference. Just as reviewer 3 commented on the preceding version, and as you commented this time, the risk of potential recombination or of a disproportionate influence on phylogeny inference of a small proportion of sites in each window (e.g. Shen et al. 2017), will be higher in windows of larger size. Nevertheless, we obtained a consistent topology in the four Astral trees inferred based on windows of different sizes. The results support the stability of the topology obtained in this kind of analysis, and suggest that the potential risks mentioned above are not serious in our case.

In addition, until better network-based methods become available, current species-tree-based methods are still useful to describe *Ficus* evolution, and to make inferences about biogeography and divergence times of major lineages. Here, we used the Astral tree to describe one of the two most plausible scenarios of *Ficus* evolution. The topology obtained was not only highly supported at almost all nodes, but was also consistent with the main tree of phylogenetic networks inferred with PhyloNetworks (Supplementary Fig. 6, as we constructed with PhyloNetworks, which does not assume a bifurcating tree, in contrast to the Astral tree). Nevertheless, we additionally describe the alternative evolutionary pathway for *Ficus* based on the primary concordance tree (PCT tree inferred with BUCKy).

Wascher, M. & Kubatko, L. Consistency of SVDQuartets and maximum likelihood for coalescent-based species tree estimation. *bioRxiv*, 523050, doi:10.1101/523050 (2019).

Taylor, S. A. & Larson, E. L. Insights from genomes into the evolutionary importance and prevalence of hybridization in nature. *Nat. Ecol. Evol.* **3**, 170-177, doi:10.1038/s41559-018-0777-y (2019).

Shen, X. X., Hittinger, C. T. & Rokas, A. Contentious relationships in phylogenomic studies can be driven by a handful of genes. *Nat. Ecol. Evol.* **1**, doi:10.1038/s41559-017-0126 (2017).

135 An extant group cannot be basal to another extant group (the correct term for this relationship in this case is “sister”)

Reply: Thanks, we replaced “as the basal group to all other *Ficus* species” by “as sister to all other *Ficus* species”

147 “Monophyletic clade” is redundant (if a group is not monophyletic, it is not a clade)

Reply: We agree. We replaced the phrase in question by the style of “the XXX group is monophyletic”. Here, we want to highlight that three *Ficus* groups are supported as monophyletic, contrasting with the preceding sentence where it is stated that the other two subgenera are not monophyletic groups. We modified the relevant text to read as follows (lines 149-153): “Within the gynodioecious clade, subgenus *Ficus* (part of the gynodioecious clade) also emerged as polyphyletic, While subgenus *Sycomorus,* subgenera *Ficus* + *Synoecia* group (“GTS-clade” in Figs. 1 and 2) and the subgenus *Sycidium* + *F. carica* group (“CC-clade” in Figs. 1 and 2) were recovered as monophyletic with strong support (Fig.1, Supplementary Fig. 1).”

151 As I expand on a little in comments about fig. 1 (below), the geographic reconstructions make me nervous given the sparseness of the taxon sampling. I don’t know enough about fig phylogeny to assess this concern, but for these inferences to be reliable, the taxon sample has to accurately represent the current range of the species of the genus (if major transitions are missing, that’s a problem). Since most of the included samples are Eurasian, I’m not surprised that much of the ancestry of the tree is reconstructed as Eurasian, but that doesn’t really tell us much (in my opinion). Also, existence in “eurasia” does not mean that the species were sympatric. How many

of the currently “Eurasian” species are truly sympatric?

Reply: Thanks for your comments. We improved this part by mentioning that a similar pattern (origin in Eurasia) was also supported by the most recent previous study with 200 fig species (Cruaud et al. 2012) (lines 154-156), as follow: “Consistent with a previous study that included 200 species[20], our dating and ancestral area reconstructions using the Astral tree based on the complete nuclear genomic data suggest that *Ficus* originated in Eurasia roughly 75 (62.39 - 87.41) million years ago (Mya) (Supplementary Figs.”

Yes, a sparse sample of species in such a species-rich genus could limit the stability or robustness of results of geographic reconstructions. However, we note that (1) our sample of species is geographically representative: most of the included samples are from Eurasia, but that is also where most *Ficus* species are found (550 of about 800 species total [Berg and Corner 2005]).Furthermore, (2) most fig sections are restricted to a particular region (with only four sections not in Eurasia: two sections endemic to the Neotropics, one to Africa, one to Australia; and the first three of these four sections were included in this study), so that a species is representative of the geographic region of the entire section (See Method part, lines 443-454) . Finally (3), within a given region, most fig clades (and species) are sympatric even at a quite local scale (Harrison 2005). For example, in one of our research sites, Xishuangbanna, about 100 species of figs coexist (Wu et al. 2003) representing all six subgenera and 14 of the 19 main sections of the genus *Ficus*. Thus, although sparse, our taxon sample is representative, and many Eurasian species from different sections are truly sympatric, even at the local scale.

Berg, C. C. & Corner, E. J. H. in *Flora Malesiana Series I -Seed Plants* Vol. 17 (ed H.P. Nooteboom) 1-702 (Nationaal Herbarium Nederland, 2005).

Harrison, R. D. Figs and the diversity of tropical rainforests. *Bioscience* **55**, 1053-1064, doi:10.1641/0006-3568(2005)055[1053:Fatdot]2.0.Co;2 (2005).

Wu, Z., Zhou, Z.-K. & Michael, G. G. in *Flora of China* Vol. 5 (ed Flora of China Committee) 21-73 (Scientific Press & Missouri Botanical Garden Press, Beijing&St. Louis, 2003).

161 Misused “basal”

Reply: we replaced the “the basal nodes” with “the deepest nodes”.

167 I don’t think this result—highly supported trees—is at all notable when whole-genome data are used (rather, I would expect such support).

Reply: We accepted your comments and removed the “Notably”. Yes, it is not surprising that the tree based on whole-genome data is highly supported. We used the “notably” to highlight the fact that the two organellar genomic phylogenies are strikingly different from the nuclear phylogenies. But the word was put in an unsuitable place, and we removed it in this revised version.

169 This result (the incongruence between the plastid and mito genome inferences) still seems like a warning sign to me. While I conceded that the authors’ explanation (in the response document), invoking biparental inheritance and heteroplasmy, is possible, it feels like a significant leap to attribute these results to such processes. I would want, at a minimum, some evidence that these processes occur at appreciable rates, and preferably some evidence that they do so in Ficus. Otherwise, there are a whole host of other explanations for this incongruency, including lots of opportunities for inference errors. A search of the literature for intra-linkage incongruence will reveal other cases.

Reply: We cite references showing that such processes (biparental inheritance and heteroplasmy) do indeed exist in many plant groups, and are more common than previously thought (Ramsey and Mandel 2019, Hu et al. 2008, Zhang et al. 2003). However, systematic investigation of these processes is still limited. We have not found previous evidence of these processes in *Ficus*. A recent review (Ramsey and Mandel 2019) pointed out that organellar genome heteroplasmy appears to be particularly frequent in gynodioecious plants. Interestingly, almost half of *Ficus* species (~400 spp.) are gynodioecious. We agree that further investigation of these processes is required in *Ficus*. Although the mechanism is unclear, the pattern of conflict between chloroplast and mitochondrial genomes does fit the expectation of frequent hybridization inferred by other hybridization-detection methods.

As stronger evidence on the mechanism behind this pattern is still lacking, we cancelled several strong words, and further toned down the related discussion, as follows (lines 285-290): “Both *Ficus* organelle genomes have clearly been switching nuclear backgrounds throughout their history, consistent with the results of Bruun-Lund et al.[38] (Fig. 4, Supplementary Table 8), and in

ways that suggest that hybridization can result in both maternal and paternal inheritance of the organellar genomes in *Ficus*. We note that biparental and paternal inheritance of organellar genomes, as well as organelle heteroplasmy, have been shown in an increasing number of Angiosperms[50-52]."

Ramsey, A. J. & Mandel, J. R. When one genome is not enough: organellar heteroplasmy in plants. *Annual Plant Reviews online* **2**, 619-658, doi: 10.1002/9781119312994.apr0616 (2019).
Hu, Y. C., Zhang, Q., Rao, G. Y. & Sodmergen. Occurrence of plastids in the sperm cells of Caprifoliaceae: Biparental plastid inheritance in angiosperms is unilaterally derived from maternal inheritance. *Plant Cell Physiol.* **49**, 958-968, doi:10.1093/pcp/pcn069 (2008).
Zhang, Q., Liu, Y. & Sodmergen. Examination of the cytoplasmic DNA in male reproductive cells to determine the potential for cytoplasmic inheritance in 295 angiosperm species. *Plant Cell Physiol.* **44**, 941-951, doi:DOI 10.1093/pcp/pcg121 (2003).

175 I would argue that the contrasting results from the two analysis approaches indicates a need for further analytical attention, rather than for taking one result over the other.

177 Applying the same method four times and getting high support each time is not a strong indication of the reliability of the conclusion (the fact that the data were treated differently each time helps, but those differences are relatively small).

Reply: We reply to these two questions together, as they are closely related. We agree with your view on "line 175" of the previous version regarding the conflict between the Astral tree and the primary concordance tree, and in fact we proceeded as you recommend. We conducted further analysis (inferring phylogenetic networks with PhyloNetworks) to try to determine which of these two trees was better. The main tree of phylogenetic networks supports the topology of the Astral tree. That is the most important reason why we mainly focused our subsequent discussion on results based on the Astral tree. Meanwhile, as you note, we also performed the same set of analyses based on the PCT tree. As our reply to one of your first major comments (lines 49-135 above), given the frequent gene exchange inferred here, all methods of phylogenetic reconstruction based on bifurcating trees will have difficulties in describing the real evolutionary history of *Ficus*. However, phylogeny reconstruction based on bifurcating trees is still useful in

inferring biogeographic history, at least until more powerful methods based on phylogenetic networks become available. We also further improved a related part (lines 183-189) as follow: “Analyses based on the Astral tree and PCT tree both support the interpretation that all main clades of *Ficus* co-occurred sympatrically during portions of their biogeographical histories. However, Astral and PCT trees suggest different clades as being the sister group to the rest of *Ficus* (*Urostigma and Pharmacosycea*, respectively). Given its strong agreement with PhyloNetworks[45] analyses (see below) (Supplementary Fig. 6), we will primarily focus on the inferences based on the Astral tree topology for the remaining analyses. However, we will also consider the implications of the alternative PCT tree topology.”

Concerning your comment on “line 177” of the previous version: As we replied above in our answer to your above comment (lines 422-464, above), our reason for inferring the Astral tree four times was to use four genomic windows of different size, to test for a potential artifactual effect of window size already noted in the literature (greater effect of gene recombination in larger windows). This procedure was thus not at all “applying the same method four times and getting high support each time”—rather, it allowed us to show that the result was robust to variation in window size.

186 “four-taxon”

Reply: We modified it.

191 How is was this conclusion (“at least one hybridization event”) drawn? ABBA-BABA tests provide a measure of the statistical support for introgression, rather than a count of events, right?

Reply: Here we explain how events can be counted. ABBA-BABA D-statistics test whether or not there is statistical support for introgression having occurred among members of a given “four-taxon group”. When introgression of one species with another species of the group is detected, this is deemed a hybridization event. As one species can be involved in many different “four-taxon groups” with the other three ‘partner’ taxa changed, we could find multiple hybridization events between the given species and other species. Thus, it is possible to have multiple hybridization events involving a given species or taxon.

We have explained this information in Method part (lines 590-595), as follow: "The doAbbababa2 program could calculate the *D*-statistics for all potential four-taxon combinations of species or groups, but only those four-taxon groups that followed the phylogenetic topology of the Astral species tree were considered. In a few cases, results of some alternative four-taxon groups were also kept when the phylogenetic topology among taxa was unstable (Supplementary Table 6)."

201 I am uncomfortable with the results from each of these data-clustering approaches (window size) being treated as independent tests. These are not independent—if a result is found using one window size, and a different result is found using another window size, that means that neither result is reliable, rather than that both are reliable.

Reply: It is possible that datasets differing in window size might detect different hybridization events, as we will explain below. We were particularly concerned with the potentially higher risk of gene recombination within larger genomic windows, which would influence the accuracy of hybridization detection. As a conservative strategy, we thus removed the PhyloNetworks results based on the largest windows (1000SNP windows dataset, mean window size ~1 Mb, range 438 Kb-4.16 Mb), keeping only the results based on the short windows dataset (windows size 500 kb). This window size was also used in a paper recommended by Reviewer 3 (Figueiro et al. 2017).

Here, we extend our explanation of why it is reasonable to obtain different results with PhyloNetworks based on datasets differing in window size. There is a relationship between the time passed since hybridization and the size of chromosomal blocks in hybrids (Fisher 1954, Ungerer et al. 1998, Buerkle et al. 2008). After the initial hybridization event, the size of parental chromosomal blocks within the hybrids will continuously be reduced over time, as a result of recombination during meiosis in heterozygous individuals. In other words, the introgressive genomic block due to a very ancient hybridization event will be shorter than that due to a relatively recent event. The signals of ancient hybridization events could thus be covered by derived signals if quite long genomic windows are used. Based on this explanation, we can expect that short genomic windows have an advantage in detecting more ancient hybridization events, while long genomic windows are better at detecting more recent events. Our results support this hypothesis: the PhyloNetworks analysis based on the 1000SNP windows dataset inferred only two

hybridization events, whereas the PhyloNetworks analysis based on the 500kb windows dataset inferred five hybridization events, with greater involvement of deep nodes (i.e., more ancient hybridization events) (Fig.2, Supplementary Fig. 6). Meanwhile, all hybridization events within *Ficus* clades detected with PhyloNetworks were corroborated with ABBA-BABA *D*-statistics.

Figueiro, H. V. *et al.* Genome-wide signatures of complex introgression and adaptive evolution in the big cats. *Sci. Adv.* **3**, e1700299, doi:10.1126/sciadv.1700299 (2017).

Fisher RA. A fuller theory of “junctions” in inbreeding. Heredity **8**, 187–197 (1954).

Baird, S. J. E. A simulation study of multilocus clines. *Evolution* **49**, 1038-1045, doi:10.2307/2410429 (1995).

Ungerer, M. C., Baird, S. J. E., Pan, J. & Rieseberg, L. H. Rapid hybrid speciation in wild sunflowers. *Proceedings of the National Academy of Sciences* **95**, 11757-11762, doi:10.1073/pnas.95.20.11757 (1998).

Buerkle, C. A. & Rieseberg, L. H. The rate of genome stabilization in homoploid hybrid species. *Evolution* **62**, 266-275, doi:10.1111/j.1558-5646.2007.00267.x (2008).

207 But it is not just biological processes that need to be considered here. Stochastic variation in the evolutionary process, model mis-specifications, labwork errors, etc., can all lead to discordance.

Reply: As we have already replied above in our responses to your first and second major comments (Lines 49-248), we strictly controlled the quality of our data, minimizing the likelihood of those potential artificial errors. Concordance of results from multiple standard analyses, with multiple, independently collected, and analysed datasets, also inspires our confidence in our results. Although the possibilities you mention can never be entirely discounted, as no one knows the real path that evolution followed, our analyses all point to the conclusion that hybridization was frequent during *Ficus* evolution. We feel we have presented a strong case, that our hypotheses are both testable, and that the procedures we used to test them are repeatable.

209 BUCKy doesn’t find hybridization events—need some other wording here to describe the conclusions drawn from the BUCKy inferences

Reply: We replaced “found” by “inferred”, and modified the sentence in question (Lines 212-213) as follows: “In the BUCKy analyses, all *Ficus* main clades were inferred to be involved in at least one hybridization event, with…”

211 Basal misused

Reply: we corrected the word, and modified the sentence in question (Lines 214-215) as follows: “We note that section *Pharmacosycea* (which the PCT tree suggests is sister to all other figs) currently exhibits an exclusively New-World distribution.”

215 A more compelling treatment here, in my opinion, is to only report and discuss the inferences that are supported by ALL the analytical approaches applied. That would also make the results/discussion easier to follow.

Reply: All three different methods agree that there have been many major hybridization events throughout the evolutionary history of *Ficus*, and in most cases they also agree on what the specific events were that took place between different taxa. However, they also identify (in some cases) different hybridization events. These differences present testable hypotheses that can be resolved with more data in studies where the sampling is specifically designed to address the specific differences in inference. We note that these methods infer hybridization based on different aspects of the data, theory, and assumptions, and have different limitations.

As we replied above in our responses to your first major comments, the most powerful evidence supporting our conclusion of frequent hybridization comes from results of the ABBA-BABA *D*-statistics. This method is also not affected by the whole phylogenetic topology. It identifies gene flow based on detecting the genome-wide excess of shared derived alleles using four-taxon groups, and the robustness of this method has been widely tested (Green et al. 2010, Durand et al. 2011, Martin et al. 2013, Árnason and Halldórsdóttir 2019). Our analysis with this method (and supported by others) suggests that frequent hybridization has involved all main fig clades and studied species (supplementary Table 6). Although results from two additional methods, PhyloNetworks and BUCKy, potentially suffer some risk of reduced accuracy, giving potentially less accurate estimations of topology owing to hybridization-mediated genomic recombination (as you worried), these analyses also support the conclusion of frequent

hybridization along *Ficus* evolution. We note that all hybridization events inferred with either PhyloNetworks or BUCKy were also corroborated by results of the ABBA-BABA D-statistics. Thus, the main conclusion of frequent hybridization along *Ficus* evolution seems very robust.

221 What does failure to diverge mean in this context?

Reply: “failure to diverge” is one of the five types of evolutionary events defined in the JANE program. This concept represents an ecological event in which the “parasite lineage” does not diverge following the divergence of the “host lineage”, so the same “parasite lineage” is associated with two newly diverged “host lineages” (Conow et al. 2010, Figure 1). Refer to the Jane website (https://www.cs.hmc.edu/~hadas/jane/tutorial.html), where diagrams of five events including “failure to diverge” are shown as follows: (black lines indicate the “host lineage”, while blue lines indicate the “parasite lineage”, with some necessary explanation accompanying each diagram)

Cospeciation:

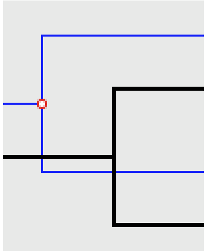

A Cospeciation is marked by a hollow colored circle. (both lineages divergence temporally and are continually associated)

Duplication:

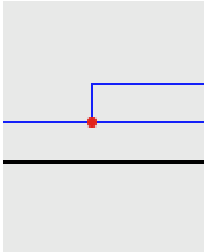

A Duplication is marked by a solid colored circle. (two divergent “parasite lineages” are associated with the same “host lineage”)

Duplication with Host Switch:

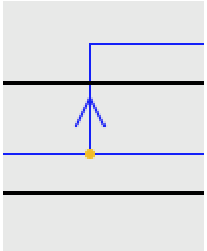

A Host Switch is marked by a duplication, with an arrow following the trajectory of the switching species. (one divergent “parasite lineage” shifted to another new “host lineage”)

Associate Loss:

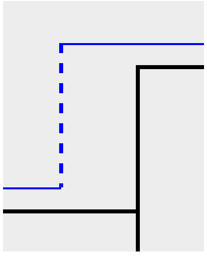

A Loss is marked by a dashed line. ("parasite lineage" only associated with one of two divergent "host lineages")

Failure To Diverge:

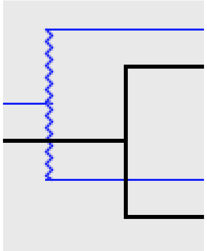

A Failure to Diverge is marked by a jagged line. ("parasite lineage" did not divergent as its "host lineage" diverged, but is continually associated with two new divergent "host lineages")

Conow, C., Fielder, D., Ovadia, Y. & Libeskind-Hadas, R. Jane: a new tool for the cophylogeny reconstruction problem. *Algorithms Mol. Biol.* **5**, doi:10.1186/1748-7188-5-16 (2010).

222 The JANE analyses attribute all incongruence to either "associate switches" or "associate losses"? What do these mean (especially "associate losses") in the context of genomic compartments within organisms? And there are many reasons for inter-compartmental incongruence that don't fall into either of those two categories.

Reply: As we stated in lines 220-225 of the preceding version of the manuscript, cophylogenetic analyses in the JANE program can infer five types of ecological events (codivergence (or cospeciation), duplication, associate switches, associate losses, and failure to diverge) to explain the current pattern of association between two phylogenies. Except for "codivergence", all the other four events could generate phylogenetic discordance. In our studies, only three of these five types of events were inferred: "codivergence" events, which produce concordance, and two other types of events ("associate switches" and "associate losses") that both produce discordance, and are consistent with the expected outcome of the process of hybridization. Meanwhile, two other kinds of events ("duplication" and "failure to diverge") could also be reasons for the phylogenetic incongruence, but were not detected by the analyses in our case. The meaning of "associate losses" has been explained in our reply above. An example of such an event would be when a

nuclear genome lineage ("host") diverges and the associated chloroplast genome lineage ("parasite" lineage) is only associated with one of the two newly divergent nuclear genomes, having lost its association with the other.

We improved a related part (Lines 226-232) as follow: "We then ran event-based cophylogenetic analysis using JANE 4.0[49] to infer the relative contributions of different potential evolutionary events in generating phylogenetic discordance (e.g., duplication, associate switches, associate losses, and failure to diverge). Notably, JANE 4.0 inferred high frequencies of both associate switch (suggestive of hybridization[50], as organellar genome lineages switch among different background nuclear genome lineages) and associate loss events ("parasite lineage" [e.g., organellar genomes] is associated with only one of two divergent "host lineages" [e.g., nuclear genomes]) (Fig. 4A, Supplementary Table 8, Appendix 8)."

241 Why are the two wasp phylogenies substantially different? As with the differences observed in this study among different analysis approaches and different datatype, this incongruence makes me nervous. If you just did a codiversification analysis of one wasp phylogeny on the other, would you find strong evidence of "host-switching" within the wasps?

Reply: The two phylogenies of pollinator wasps associated with the 15 studied fig species were not produced in this study, but were extracted from published wasp phylogenies (Cruaud et al. 2012, Machado et al. 2001). Neither of these phylogenies of pollinator wasps is as robust as those for *Ficus* trees generated here, because they are based on one or a small number of genes. They represent two alternative views, both considered plausible, of fig wasp evolution (Supplementary Fig. 5, Supplementary Table 8 [last line]).

However, to directly address your question of differences between the two published wasp phylogenies, even though the analysis is not biologically meaningful, a codiversification analysis comparing the two published wasp phylogenies using Jane did find some "host-switching" events, but their frequency was lower than in the codiversification analysis between figs and fig wasps (Table 8). This suggests that the conflict between the two wasp phylogenies is lower than that between fig wasps and host figs.

We give more detailed information on these two wasp phylogenies in Supplementary Note 3. The topological differences between them may simply be the result of the different gene matrices

used. The Machado et al. tree is based on only one mitochondrial protein-coding gene (COI) fully sequenced for all 15 wasp species that is completely sequenced for all species considered. In contrast, the Cruaud et al. tree is based on six genes (two nuclear protein-coding genes (EF1a, Wg); two mitochondrial protein-coding genes (COI, Cyt b), and two ribosomal genes (28S rRNA, 18S rRNA)) although not all genes were successfully sequenced in all of the 200 wasp species. The denser species sample and bigger gene matrix in the study by Cruaud et al. (2012) should make their phylogeny more robust, even if it is still not as robust as our fig phylogenies based on genomic data, in this study. More detailed information refers to Supplementary Note 3.

Most importantly, as we mentioned in the main text, NEITHER of the two most plausible available wasp phylogenies is congruent with ANY fig phylogeny based on nuclear, chloroplast, or mitochondrial data. Furthermore, regardless of which of the two wasp phylogenies was tested against the various nuclear and organelle-based phylogenies, pollinator host-switch events were always inferred at higher frequency than co-speciation events (Fig. 4, Supplementary Table 8). So, based on available knowledge on wasp phylogenies, our cophylogenetic reconciliation results strongly support the main conclusion that pollinator host switching has been frequent along the coevolutionary history of figs and wasps.

Machado, C. A., Jousselin, E., Kjellberg, F., Compton, S. G. & Herre, E. A. Phylogenetic relationships, historical biogeography and character evolution of fig-pollinating wasps. *Proc. R. Soc. B-Biol. Sci.* **268**, 685-694 (2001).

Cruaud, A. *et al.* An extreme case of plant-insect co-diversification: figs and fig-pollinating wasps. *Syst. Biol.* **61**, 1029-1047, doi:10.1093/sysbio/sys068 (2012).

268 This line is critical—if the methods are not identifying the same events, than I don't think their conclusions are reliable.

Reply: As we have already replied above, the three methods we used do not necessarily give the same results, because they infer hybridization based on different datasets and theory and have different limitations. Also, as we have stated, the points of difference present explicit, testable hypotheses for future studies to target and resolve. This study generates those hypotheses.

270 Demonstrating that ILS is not a sufficient explanation for the discordance does not mean that there has to be hybridization—this second part does not follow from the first (sorry to keep harping on this, but it keeps coming up).

Reply: Almost all current methods of detecting hybridization are based on testing the null model that ILS alone can explain conflicts among different parts of the genome (among loci, among different regions of the nuclear genome), or whether other biological processes (e.g., hybridization, horizontal gene transfer) must also be taken into account (Durand et al. 2011, Chung and Ane 2011, Solis-Lemus and Ane 2016). Among those biological processes, hybridization is the most common, and the "default" process invoked to explain the interspecific genetic exchange for most plants and animals. The alternative, horizontal gene transfer, is more common in microorganisms (Taylor and Larson 2019, Moran and Jarvik 2010). Our treatment of this theme is thus reasonable and reflects current practice. We expressed above information in several places of Methods parts, for examples (lines 608-612): "With the similar function of above ABBA-BABA *D*-statistics and PhyloNetworks, BUCKy provides a third way to distinguish hybridization from ILS by testing whether the distribution of gene-tree frequencies is compatible with that expected under neutral coalescent processes (ILS), or whether other biological processes must also be invoked (e.g., hybridization, horizontal gene transfer)[44,48]."

Durand, E. Y., Patterson, N., Reich, D. & Slatkin, M. Testing for ancient admixture between closely related populations. *Mol. Biol. Evol.* **28**, 2239-2252, doi:10.1093/molbev/msr048 (2011).

Chung, Y. J. & Ane, C. Comparing two Bayesian methods for gene tree/species tree reconstruction: simulations with incomplete lineage sorting and horizontal gene transfer. *Syst. Biol.* **60**, 261-275, doi:10.1093/sysbio/syr003 (2011).

Solis-Lemus, C. & Ane, C. Inferring phylogenetic networks with maximum pseudolikelihood under incomplete lineage sorting. *PLoS Genet.* **12**, e1005896, doi:10.1371/journal.pgen.1005896 (2016).

Moran, N. A. & Jarvik, T. Lateral Transfer of Genes from Fungi Underlies Carotenoid Production in Aphids. *Science* **328**, 624-627, doi:10.1126/science.1187113 (2010).

Taylor, S. A. & Larson, E. L. Insights from genomes into the evolutionary importance and prevalence of hybridization in nature. *Nat. Ecol. Evol.* **3**, 170-177, doi:10.1038/s41559-018-0777-y (2019).

426 As I said this in my earlier review, all sampled individuals should be represented by a herbarium voucher (and that voucher information provided in this manuscript). I consider this to be essential.

Reply: We added information of herbarium voucher specimens in Supplementary Table 1.

518 I don't follow how the MCMCtree analyses were done, but I haven't used this program myself. The final MCMCtree analyses used fixed substitution rates derived from the earlier PAML runs?

Reply: The final MCMCtree did not use fixed substitution rates. The fixed overall substitution rate estimated in the first step was only used in the second step to calculate the gradient vector and Hessian matrix of the branch lengths with maximum likelihood estimates under GTR+G and independent rates model. Then, in the final (third) step, MCMCtree analyses estimated the divergence time and confidence interval for each node with the approximate likelihood principle based on the values of the gradient vector and Hessian matrix of branch length files calculated in the second step. Refer to lines 530-546 for details.

544 Some model-selection and partitioning would be appropriate for the ML analyses of the concatenated organellar genomes. FastTree seems like a poor choice (from an accuracy perspective) unless slower methods were not able to run on this dataset.

Reply: We inferred phylogenies of chloroplast and mitochondrial genomic datasets with the most appropriate model (and also the most parameter-rich substitution model (GTR+GAMMA, using JmodelTest2) based on the unpartitioned whole genome sequence dataset, instead of partitioned sequences. We think this strategy is reasonable and practical. Firstly, as in many other plants, both chloroplast and mitochondrial genomes in *Ficus* are one single chromosome, and are generally maternally inherited as a unit. Meanwhile, they are generally highly conserved evolutionarily, with low mutation rates throughout almost the whole genome, which can thus be generally treated

as "one long gene". Our case was typical, with highly conserved sequences, low mutation rates, and very short branch lengths in both chloroplast and mitochondrial phylogenies (Supplementary Fig. 4), indicating that separating genes for model selection would have little effect on the phylogenies. Secondly, Bruun-Lund et al. (2017) demonstrated that the unpartitioned chloroplast genomic dataset can recover more robust phylogenies in *Ficus* compared to a dataset partitioned in genes, and also confirmed that all main regions of the chloroplast genome share the same best substitution model (GTR+GAMMA), same model were also used in our case. Moreover, many studies of organellar genomic phylogenies have shown that phylogenetic analyses with partitioned or unpartitioned datasets have no effect on the topology for the strongly supported clades/branches (e.g., Folk et al. 2017, Dell et al. 2020). In this study, therefore, both chloroplast and mitochondrial genomic phylogenies were robust in the key monophyletic clades, which can be quite stable, and partitioned analyses seem not to be necessary.

Concerning the FastTree analysis for the mitochondrial genomic phylogeny, the phylogenetic resolution of the mitogenomic tree was not as robust and has polytomies, based on bootstrap values of RAxML, which are mainly due to the relatively low number of SNPs of mitochondrial genome (supplementary Table 2). This pattern is also found in many other plants (Wolfer et al. 1987). Instead, we used the FastTree program to calculate the SH-like support values for the clades/nodes, which outperform bootstrap values when applied to polytomies of mitogenomic trees (Price et al. 2009, Simmons and Norton 2014). As mentioned above, co-phylogenetic analyses were mainly based on the topology supported with greatest confidence. Therefore, the FastTree phylogeny was more robust than the RAxML phylogeny in the mitogenomic dataset.

Furthermore, in this study, all results based on organellar phylogenies provide supportive, though more indirect, evidence for our main conclusion of frequent hybridization and pollinator host switches. Results based on the nuclear genome and on associated pollinator wasps supplied the most powerful and direct evidence for our main conclusion. Results based on the organellar genomes corroborated well the pattern demonstrated with nuclear genomic data-based analyses.

Folk, R. A., Mandel, J. R. & Freudenstein, J. V. Ancestral gene flow and parallel organellar genome capture result in extreme phylogenomic discord in a lineage of Angiosperms. *Syst. Biol.* **66**, 320-337, doi:10.1093/sysbio/syw083 (2017).

Bell, D. *et al.* Organellomic data sets confirm a cryptic consensus on (unrooted) land-plant relationships and provide new insights into bryophyte molecular evolution. *Am. J. Bot.* **107**, 91-115, doi:10.1002/ajb2.1397 (2020).

Price, M. N., Dehal, P. S. & Arkin, A. P. FastTree: Computing Large Minimum Evolution Trees with Profiles instead of a Distance Matrix. *Mol. Biol. Evol.* **26**, 1641-1650, doi:10.1093/molbev/msp077 (2009).

Wolfe, K. H., Li, W. H. & Sharp, P. M. Rates of nucleotide substitution vary greatly among plant mitochondrial, chloroplast, and nuclear DNAs. *Proc. Natl. Acad. Sci. U. S. A.* **84**, 9054-9058, doi:10.1073/pnas.84.24.9054 (1987).

Simmons, M. P. & Norton, A. P. Divergent maximum-likelihood-branch-support values for polytomies. *Mol. Phylogenet. Evol.* **73**, 87-96, doi:10.1016/j.ympev.2014.01.018 (2014).

562 A quibble, but I would say that a significant ABBA BABA result indicates that ILS is not sufficient to explain the result, but does not necessarily indicate gene flow

Reply: As we have replied in response to several similar comments above, other processes of genetic exchange, such as horizontal gene transfer (HGT), could also account for our results, but gene flow via hybridization and introgression is much more frequent among flowering plants compared to HGT, which is common in microorganisms. The pollinators are clearly the prime movers of the host fig's genes in this system, and movement is based on host recognition.

637 Basal misused

Reply: we replaced “basal clade” by “earliest-diverging clade”.

Fig1 – The response to reviews states that Antiaris is more closely related to Figus than Morus is, yet, here Antiaris and Morus are sister species—this conflict needs to be resolved. The tip states are presumably that of the tip species (so F.carica, for example, is Eurasian). But given the sparseness of the taxon sample, are there a lot of excluded range-change events? E.g., are all members of subgenus Sycidium Eurasian? If range-changes are missing, than the rates of range change will be underestimated in the model, and the reconstructions will be unreliable. This problem would be even worse if the included ranges are biased (e.g., if a disproportionately high

number of the included species are Eurasian). The scale on this figure is a little awkward – why not make the units "millions of years" and have the first labeled interval be "25", instead of making the units "100 million years" and having the first label be 0.25? The nodes on this tree that were calibrated in the divergence-time estimation should be indicated.

Reply:

1) Concerning the phylogenetic position of the newly added outgroup, *Antiaris toxicaria*.

Following your comments on our first submission, we added *Antiaris toxicaria* as an additional outgroup (the other outgroup used is *Morus alba*) of *Ficus* plants in our last revised version. *Antiaris* was recovered as closer to genus *Ficus* than is *Morus alba* in the paper by Gardner et al. (2017). That study aimed to infer the phylogeny of *Maclura*, but in fact it inferred a phylogeny for the entire family Moraceae with 18 genera from all tribes, and one outgroup from Cannabaceae family. In that study, *Antiaris* and *Ficus* are indicated lying in one clade, and *Morus* is in a very distant clade. We think the apparent discrepancy may owe to the smaller samples for other Moraceae genera and to the lack of older outgroups, for example, non-Moraceae taxa. Accurate estimation of the relationship between the genera *Morus* and *Antiaris* requires including additional older outgroups (e.g., outgroup from Cannabaceae family). However, none of this influences estimation of the relationships within *Ficus*. Once an older outgroup is included (assuming it is not too distant), relationships within the ingroup will mainly depend on the sequence data used and ingroup relationships will be quite stable. This contention is also corroborated by the consistent phylogenetic topology of our four Astral trees based on the four types of genomic windows, regardless of whether one or two outgroups were used (Supplementary Figure 1A-D).

2) Concerning the tips' distribution states and their potential influence on ancestral range reconstruction:

As we have already mentioned in our reply to one of your comments above (Lines 467-496), our taxon sample, although sparse, represents well the current geographic range of the sections to which they belong, as most *Ficus* sections and all six subgenera are sympatric and distributed in Eurasia currently. We also improved this part of the text (lines 154-156) by mentioning that a

similar conclusion (origin in Eurasia) was also supported by the most up-to-date study with 200 fig species (Craud et al. 2012).

Some fig sections centered in Eurasia do have species whose ranges also include other regions, or a few species that are only distributed in other regions. For example, 10 of 115 species of subgenus *Sycidium* are currently found only in the Afrotropics (Berg and Corner 2005, Page 170, and also: http://www.figweb.org/Ficus/Checklists/Checklist_Afrotropical_Ficus.htm). However, a previous study (Cruaud et al. 2012) found that all Eurasia-dominant groups originated in Eurasia, with their earliest-diverging lineages also currently distributed in Eurasia. So, missing a few younger species or subclades with range-change events would not influence our reconstruction of ancestral distribution of *Ficus*, because those younger taxa just represent recent range extensions or range shifts.

3) For the time scale on Figure 1:

We accepted your comments and modified the time unit to millions of years for ease of understanding. Meanwhile, we also added the information on calibration of nodes. Thank you for your helpful comments.

Berg, C. C. & Corner, E. J. H. in *Flora Malesiana Series I -Seed Plants* Vol. 17 (ed H.P. Nooteboom) 1-702 (Nationaal Herbarium Nederland, 2005).

Cruaud, A. *et al.* An extreme case of plant-insect co-diversification: figs and fig-pollinating wasps. *Syst. Biol.* **61**, 1029-1047, doi:10.1093/sysbio/sys068 (2012).

Fig2 – The subfigure labeling (A and B) should be switched. Some of the inferred events here are impossible, because they occur between an ancestor and its descendent. For example, the green line that connects the ancestral lineage of Sycomorus and the GTS clade with the F.gasparriniana branch. This further highlights to me that these incongruence analyses are picking up things other than signals of hybridization. B: again, BUCKy can’t “detect” hybridization events—you can infer hybridization events from BUCKy output, but that’s a somewhat different matter. As with figure A, to be biologically possible, both participants in a hybridization have to be contemporaneous, and I’m not sure that is true of all the inferred events on this tree (I don't see any ancestor-descendent pairs, but it’s still not clear that the branches can be rescaled to allow all those events

to have contemporaneous participants. If it is possible, it would require pretty dramatic resizing of branches, such that the divergence time estimates would have to have been wildly off).

Reply:

1) Thank you for pointing this out. Our previous Figure 2, with two subfigures (A and B) aimed to highlight the hybridization results of ABBA-BABA D-statistics (shown in Figure 2A), as ABBA-BABA D-statistics inferred all hybridization events, which inferred by PhyloNetworks or BUCKy. However, the figure needed more space for better visualization. We thus moved the previous Figure 2B (hybridization inferred with BUCKy) to supplementary files as Supplementary Figure 7. We kept the previous Figure 2A (Hybridization events within *Ficus* inferred with ABBA-BABA *D*-statistics and PhyloNetworks) in the main text because it has covered all detected hybridization events in this study.

2) Concerning your argument that the figure depicts inferred hybridization events between an ancestor and its descendant, which you consider impossible: Yes, such events would be biologically impossible if we only considered currently extant species or clades. However, the events depicted in the figure are NOT impossible if we take into account the (strong) likelihood that some lineages within these clades have gone extinct. Traces of ancient hybridization involving such "ghost" lineages can be preserved in the genomes of extant lineages. Species extinctions are very common and this kind of ancient hybridization must be considered in hybridization detection. In fact, similar cases of "the ghost of ancient hybridization" have also been found in other taxa (Árnason and Halldórsdóttir 2019 [similar cases detected with the same method as used here, PhyloNetworks], Luo et al. 2017) with some of the most well-known cases in humans (Kuhlwilm et al. 2016). Thus, when we take into account "the ghost of ancient hybridizations", all the inferred hybridization events shown in Figure 2A are plausible. Meanwhile, those "ghost lineage hybridizations" are not the case for many other hybridization events detected (Fig.2).

To avoid potential confusion, we also added one sentence in the Results section explaining the pattern as follows (Lines 280-282): "Interestingly, several hybridization events suggest ancient "ghost" hybridization events involving a now-extinct ancestor and an extant descendant[47] (Fig. 2, Supplementary Fig. 6-7)."

3) For hybridization “detected” with BUCKy, we accepted your comments and replaced “detected” by “inferred” in the new Supplementary Fig. 7.

Árnason, E. & Halldórsdóttir, K. Codweb: Whole-genome sequencing uncovers extensive reticulations fueling adaptation among Atlantic, Arctic, and Pacific gadids. *Sci. Adv.* **5**, eaat8788, doi:10.1126/sciadv.aat8788 (2019).

Kuhlwilm, M. *et al.* Ancient gene flow from early modern humans into Eastern Neanderthals. *Nature* **530**, 429-433, doi:10.1038/nature16544 (2016).

Luo, X. *et al.* Chasing ghosts: allopolyploid origin of *Oxyria sinensis* (Polygonaceae) from its only diploid congener and an unknown ancestor. *Mol. Ecol.* **26**, 3037-3049, doi:10.1111/mec.14097 (2017).

Fig3 In cases where sister species could be rotated to avoid crossed lines, that would probably be more appropriate here (otherwise the crossed lines give the impression of incongruence where none exists). So in fig D the two figs with Tetrapus pollinators could be rotated to show that that’s a congruent relationship, and same for the bottom branch in fig C.

Reply: Thanks, we accepted your comments and modified the figures.

Fig4 I’m not sure what these different events would refer to, especially in the comparisons of the different genomic compartments, but regardless, it looks like no Duplication or Fail Div. events occur. If these events are not realistic for this system, they should be removed from the model to avoid artifacts.

Reply: As we have already replied in our response to one of your comments above (lines 713-738), we explain in detail the meaning of each of the types of events. In our cophylogenetic analyses we inferred no “duplication” and “Failure to diverge” events. However, those events are possible in this system and indeed have been inferred in some very recent studies of fig-fig wasp systems (Wang et al. 2019, Satler et al. 2019), and for this reason we maintain mention of them.

Wang, A.-Y. *et al.* The nature of interspecific interactions and co-diversification patterns, as illustrated by the fig microcosm. *New Phytologist* **224**, 1304-1315, doi:10.1111/nph.16176 (2019).

Satler, J. D. *et al.* Inferring processes of coevolutionary diversification in a community of Panamanian strangler figs and associated pollinating wasps. *Evolution*, doi:10.1111/evo.13809 (2019).

##

Reviewer #3 (Remarks to the Author):

Overview:

I reviewed a previous version of this manuscript and commend the authors for the addition of many new analyses and changes to the manuscript text that have simplified the overall message. I thank the authors for the additional supplementary notes and appendices that have addressed previous questions as well. My comments are thus limited to these additional or edited sections of text.

Reply: Thanks very much for your helpful comments on the preceding version, which greatly improved the quality of our manuscript.

Major Comments:

I find discussion section “Mechanisms of hybridization: signaling and pollinator recognition of hosts” a bit disconnected from the rest of the paper. Although the speciation mechanisms outlined here seem reasonable, none of the results in the manuscript are referenced here. Are the citations regarding different profiles of volatile compounds among Ficus species and their associated pollinator species relevant to the lineages sampled here? In short, is there a way to connect any of this past research, especially those regarding when wasps can oviposit in distantly related species, to any of the introgression events detected here? If not, lines 345-353 in the second paragraph seem repetitive and vague and can probably be cut down. Some specific examples could also help make this section less vague to those who are not experts in the fig-fig wasp system.

Reply: Thanks for your helpful comments. We agree that inclusion of some specific examples that suggest speciation mechanisms would be very useful. We cited some examples from the literature, but did not describe the findings in detail in the text, owing to space considerations. Following your suggestion, we present two such cases in the Discussion section, and briefly describe them

here. In the first case, Ramirez (1994) documented hybridization events between figs of different sections (*F. religiosa* and *F. aurea*, belonging to different sections within subgenus *Urostigma*) and even of different subgenera (*F. religiosa* and *F. septica*, the latter belonging to subgenus *Sycomorus*), via pollinator switches, demonstrating the plausibility of our inferences of hybridization between distinct fig clades (including between these very same sections and subgenera). We added this case just before the section "Notably, Ramirez[36] documented hybridization events between introduced *F. religiosa* and native *F. aurea*. These species are included in our genomic and phylogenetic study and belong to different sections within subgenus *Urostigma*. Perhaps more remarkably, he documented viable hybrids between *F. religiosa* and *F. septica*, which belong to different subgenera (the latter belonging to subgenus *Sycomorus*), and exhibit different sexual systems (monoecious and dioecious, respectively). Both cases studied by Ramirez involved visits by pollinators of a native fig species into figs of another species introduced into its range by humans (*F. religiosa*) [36]. These and other[31,37] examples support the plausibility of our phylogenetic and genomic inferences of hybridization between distinct figs from these very same sections and subgenera (Fig. 2, supplementary Table 6)."

The second case, from one of our previous studies (Wang et al. 2016), documents hybridization between co-occurring naturally sympatric figs. This case shows how similarity in volatile compounds emitted by figs of different species can lead to "mistake" visits resulting in hybridization, supporting the plausibility of our hypothesis on speciation mechanisms. We added the following passage under this section (lines 359-364): "One of our previous studies[22] included *F. hainanensis* (one of the 15 species sampled in the present study) and four other figs of subgenus *Sycomorus*. We found that those fig species with the highest rates of "mistake visits" to their receptive figs by wasp species other than their most common pollinator, emitted volatile blends that were similar to those of the normal host of that wasp. We also found that hybrid trees occurred most frequently between those fig species with the most similar chemical blends."

Ramirez, W. Hybridization of *Ficus religiosa* with *F. septica* and *F. aurea* (Moraceae). *Rev. Biol. Trop.* **42**, 339-342 (1994).

Wang, G., Cannon, C. H. & Chen, J. Pollinator sharing and gene flow among closely related sympatric dioecious fig taxa. *Proc. R. Soc. B-Biol. Sci.* **283**, doi:10.1098/rspb.2015.2963 (2016).

Minor Comments:

Lines 88-90: “evidence suggesting potential discordance” … “that is consistent with potential discordance”. This sounds very weak when it looks like the evidence is quite good.

Reply: Thanks. We modified this sentence using a stronger tone in Lines 89-91, as follows: “Specifically, both the apparent discordance of both the fig-fig wasp phylogenetic relationships and the nuclear-chloroplast associations of figs across sections of figs is consistent with the possibility of hybridization at even these deeper taxonomic scales[15,20,32,38].”

Lines 222-225: The authors state there are three events when in Table S8 there are 5 events used for computing the cost scores. This is clear in the methods on lines 616-617, but not clear here. The distinction between cospeciation/codivergence events and other event types can probably be more clearly explained here, and how those non-cospeciation events could be relevant to introgression within host phylogeny.

Reply: Thank you for mentioning these points; our writing could have been clearer. We should have stated that of the five kinds of events, three were detected in our case. We also agree that we could have better explained how these different kinds of events are related to introgression. We improved the text related to this point (Lines 223-232) as follows: “Regardless of whether the Astral or PCT topology was used, we repeatedly detected significant phylogenetic discordance among the nuclear, chloroplast, and mitochondrial genomes of *Ficus*, and the hypothesis of strict codivergence (both lineages diverge at the same time and are continually associated) among them was clearly rejected (Fig. 3A-C). We then ran event-based cophylogenetic analysis using JANE 4.0[49] to infer the relative contributions of different potential evolutionary events in generating phylogenetic discordance (e.g., duplication, associate switches, associate losses, and failure to diverge). Notably, JANE 4.0 inferred high frequencies of both associate switch (suggestive of hybridization[50], as organellar genome lineages switch among different background nuclear genome lineages) and associate loss events (“parasite lineage” [e.g., organellar genomes] is

associated with only one of two divergent “host lineages” [e.g., nuclear genomes]) (Fig. 4A, Supplementary Table 8, Appendix 8).”

Lines 255-258: I do not think this is a failure of the data or should be perceived as a negative message at all. Introgression between the early diverging lineages (e.g. Urostigma or Pharmacosycea) and the more recently diverged ones was clearly demonstrated. We should expect these types of introgression events to mislead the species tree estimation (see Jiao et al. 2020), especially given the short internal branch lengths of the Astral trees in Supplementary Figure S1 relative to the Ficus MRCA height and the inferred gamma parameter is around 0.2 between F. cyanthistipula and F. triloba from PhyloNetworks in Supplementary Figure S6. The conflict in species tree relationships across methods will probably persist even with additional taxon sampling because of the difficulty of estimating a single binary species tree with both short internode distances between speciation events (high ILS) and the high levels of introgression between non-sister taxa.

Reply: Thanks very much for these very useful comments and the associated reference. Your comment not only explains the apparent conflict among species trees based on different methods, but also further corroborates the existence of frequent gene flow between *Ficus* lineages. Following your comments, we modified the part of the text in question (lines 319-325) as follows: “Despite the depth of genetic sampling, analyses of these data do not unambiguously resolve the phylogenetic relationships among the recognized *Ficus* clades, especially at important basal nodes. What these complete genomic data do unambiguously indicate is that hybridization and introgression of components of the nuclear genome, and exchange of entire mitochondrial and chloroplast genomes, have characterized *Ficus* throughout its evolutionary history.” We also highlight the potential effect of introgression events to mislead the species tree estimation in discussion part (lines303-306) as follow: “The massive *Ficus* hybridization events detected here also help reconcile the inconsistency between inferred species trees using different methods, and in general help account for previous challenges in using either morphological or molecular data to resolve robust *Ficus* phylogenies [20,57].”, and further highlight in the conclusion part (line 411-413) as follow: “The rampant hybridization that our complete nuclear, mitochondrial, and

chloroplast genome data reveal and substantiate helps to explain why resolving fig phylogenies and fig-wasp co-radiation has been so difficult and controversial[20,21,23,38,57,69,70].”

Line 517: Is there a citation for this recommendation?

Reply: The strategy of 10 partitions of the whole genome in MCMCtree analysis was recommended by Dr. Tianqi Zhu during her training course on MCMCtree, mainly based on the following reference (which were cited in Lines 625 (as ref 84) of revised version):

Zhu, T. Q., Dos Reis, M. & Yang, Z. H. Characterization of the uncertainty of divergence time estimation under relaxed molecular clock models using multiple loci. *Syst. Biol.* **64**, 267-280, doi:10.1093/sysbio/syu109 (2015).

Line 521: Cite dos Reis et al. (2011) for the approximate likelihood method.

Reply: Thanks. We cited it as ref 86.

dos Reis, M. & Yang, Z. Approximate likelihood calculation on a phylogeny for bayesian estimation of divergence times. *Mol. Biol. Evol.* **28**, 2161-2172, doi:10.1093/molbev/msr045 (2011).

Line 539: The use of “Supplementary Table” in both the Supplementary Information and Appendices is a bit confusing. I was looking for the BioGeoBears model comparison table in the SI until I realized the meaning was that it was in the excel file in the appendix. Maybe some different naming for tables in the appendices is needed. Also, both the BioGeoBears and nQuire tables are rather simple and could be included in the SI pdf rather than in the excel spreadsheet in the Appendix.

Reply: Thanks. We moved both the BioGeoBears and nQuire tables to the Supplementary Information file. Meanwhile, we also renamed the tables in the Appendix to avoid potential confusion when trying to locate them. Now, we only have Supplementary figures or tables, Appendices 1-9, and Source data 1-2.

Line 575: Delete this sentence rather than say there are results but not show them.

Reply: We removed this sentence.

Line 595 and throughout: There are several abbreviations that are defined multiple times or in some cases spelled out. These are BCA, CF, PCT. Please check these are defined at first use and be consistent with the abbreviations throughout.

Reply: Thanks. We checked these abbreviations, and have defined each of them at its first mention in the paper, and consistently used only the abbreviations in subsequent mentions.

Figure 5B: Do the pollinator switch events coincide or make sense in the context of inferred introgression events? This does not need to be addressed in the figure legend, but the pollinator switching events beg some discussion in the section "Cophylogenetic analyses and detection of associate switches using cytoplasmic genomes and pollinator phylogenies".

Reply: Thanks for your helpful suggestion. Yes, all pollinator switch events inferred with the cophylogenetic analyses were corroborated by associated inferred introgression events, and we have summarized this matching in Supplementary Table 9 in the preceding version. In this version, we followed your suggestion and added the following passage in the Methods section titled "Cophylogenetic analyses and detection of associate switches" (line 629-636) as follow: "Discordance between nuclear and organellar genomes, especially the associate (host)-switch events, indicates organellar genome exchange among *Ficus* taxa with different nuclear genomes, indirectly supporting the occurrence of hybridization between *Ficus* species. Host-switch events inferred between fig and pollinator phylogenies indicate pollinator host switches among *Ficus* taxa. Considering the close biological relationship between the fig/pollinator wasp interaction and *Ficus* hybridization, detection of corresponding host switches in both organellar genome and pollinator wasps between the same pair of *Ficus* taxa would strongly suggest that pollinator host-switches generate *Ficus* hybridization."

It is probably not appropriate to include Illumina's proprietary documents regarding index switching in the Appendices. The text in the manuscript is sufficient.

Reply: We removed Illumina's proprietary documents from the Appendices.

References:

Jiao X, Flouri T, Rannala B, Yang Z. 2020. The impact of cross-species gene flow on species tree estimation. Syst Biol. doi: https://doi.org/10.1093/sysbio/syaa001.

dos Reis M, Yang Z. 2011. Approximate likelihood calculation on a phylogeny for Bayesian estimation of divergence times. Mol Biol Evol. 28:2161-2172.

Reply: Thanks for the very helpful references.

Reviewer #2 (Remarks to the Author):

The authors have done a herculean job in responding to reviews and revising the manuscript, and have addressed some of my concerns (thank you!). My main broad concern, however, is basically the premise of the paper—that incongruence is necessarily indicative of hybridization—and I don't think it can be addressed. The other reviewers seem to be satisfied, so I am happy leaving this issue to the discretion of the editors. Below I respond to the responses, and describe my remaining concerns. I should also note that I appreciate the framing of this paper as a "hypothesis generator" that invites further study. Additional emphasis on that framing would alleviate some of my concerns about the methodology.

I think I may be being slightly unclear when I say "robust" phylogenetic analyses. This paper includes a very large molecular dataset, so I am not at all surprised that the inferences are strongly supported—that is not my issue. I am concerned, however, that systematic biases, which have the potential to become stronger with additional date, are not adequately investigated and accounted for. The ASTRAL results, for example, are not reliable, regardless of how strongly supported they are, if each input "gene" is not its own unlinked nonrecombining unit, and I don't think either criterion has been demonstrated in this paper (that each locus is free of recombination, and that the loci are not linked to each other). To reiterate—I do not think more data are needed (at least, not more character data), but I do think greater attention could be paid to potential sources of systematic error.

The authors' point—"Most important, what we point out is that (i) neither of the two principal published phylogenies for fig wasps are congruent with any of the phylogenies (based on nuclear {Astral OR PCT tree}, chloroplast, or mitochondrial data {estimated using maximum likelihood approach}) for figs"—is well-taken. But that fact (the incongruence of all published fig phylogenies with all published wasp phylogenies) is not, in my opinion, sufficient evidence for the processes that this paper is claiming to establish. To give an extreme example, given that I know nothing about fig phylogeny, I could draw a fig phylogeny that would likewise be incongruent with the wasp phylogeny, and that wouldn't teach us anything about host switching or speciation. The absence of congruence is not sufficient evidence for these evolutionary processes.

The additional details on the dual-indexing is helpful, but my question was about the extraction through to library prep. steps (and specifically the extraction). The comparison with new nanopore and PacBio datasets could be helpful in this regard, but that comparison is reduced to a single value ("inter-taxa genomic similarity"), which I can't imagine has the resolving power to detect small amounts of contamination, and won't help if the contaminating taxon isn't among the nine species compared (if the contaminant isn't one of the focal pair, than it would elevate the non-shared kmer count, rather than the shared kmer count, and would reduce the score). I note that the authors have still not addressed my original question (this is my third time asking), about whether specific sequences were shared among samples (identical sequences, which would indicate very recent hybridization, or contamination).

"Therefore, as ML trees for both chloroplast and mitochondrial phylogenies have higher bootstrap values at most nodes than do tetrad trees (in the case of mitochondrial phylogenies, much higher values), we chose to use ML trees for both cytoplasmic-genome phylogenies, following reviewer 3's comments." This point doesn't affect the ultimate decision, but higher bootstrap values are not an appropriate justification for selecting an approach—just because the support is higher, doesn't mean that the phylogeny is closer to the truth. Also, the issue with the quartet-based approach is not that there's not enough SNPs but rather that the whole mito genome is one linkage group, and the method assumes unlinked loci. This is the sort of attention to the assumptions of the methods that I am concerned about with a paper of this scope.

"We suggest that these hybridization events are a dominant feature of fig/wasp coevolutionary

history, and by generating novel genomic combinations in the figs have contributed to the remarkable diversity exhibited by this mutualism." As before, I find this to be an interesting idea, but conjectural. Even if one accepts that there is something usual about the species richness of Ficus (I am unconvinced), there is not evidence in this paper of a link between host-switching or hybridization and speciation. I suspect that if one were to poll evolutionary biologists, most would think that specialized pollinator fidelity would lead to higher speciation rates, and that hybridization would reduce diversification.

"We presented the tetrad tree (same method used as for the SVDquartets tree) in our first version. Generally, this method gives results consistent with those given by BUCKy, but we removed the results of tetrad trees owing to other potential weaknesses of this method (see Reviewer comments and our reply above, lines 249-284)." The concerns raised about the "tetrad tree" in the previous versions were about its application to organellar data, which are nonrecombining, and therefore a single linkage group. That concern does not apply to the nuclear genome, where these approaches would indeed be appropriate (and where the ASTRAL approaches, as implemented here, are not, for the reasons described previously).

"Nevertheless, we obtained a consistent topology in the four Astral trees inferred based on windows of different sizes." I appreciate this step, but am concerned that the window size still wasn't small enough to control for recombination (and there's a trade-off, because if the window is too small, there won't be enough signal to infer a reliable genetree in the first place). Note, also, that it's not just a question of what the "main" bifurcating signal is. Because this paper is interested in hybridization (and applying BuCKy etc), the minority signals, such as one might get from a subset of loci including recombination events, is also important. The cited Shen et al. paper makes a related point, which is that a handful of "bad" genes can have a disproportionate influence on the results.

ABBA BABA counts of hybridization events—so the number of significant introgression events for each four-taxon tree were added together to the total number of counts? It would be helpful to clarify more precisely how this was done, as an inference from a given four-taxon tree tests for introgression among those lineages, not just the tip species themselves. I.e., if there is a five-taxon tree -- ((A,B), C), D), E) -- and both the ACDE and BCDE four-taxon trees show an introgression, it could be because there are indeed two, or there could be just one, involving the ancestor of A and B. (I'm sorry, I'm having trouble explaining this clearly). This also relates to the comments about "ghost" lineages, later in the response to reviews). (A ghost lineage that is/was sister to A+B could result in a positive ABBABABA between A or B and another extant lineage, but not between A or B and an ancestor).

"Based on this explanation, we can expect that short genomic windows have an advantage in detecting more ancient hybridization events, while long genomic windows are better at detecting more recent events." -- I understand why the haplotype block size changes with the age of the hybridization event, but am not convinced that applying windows of a particular size blindly on the genome would capture that signal, and especially don't understand why one would expect that large genomic windows would be better at detecting recent events. The smaller windows will have greater resolving power in all cases. (Provided that they have enough phylogenetic signal). I'm also not clear that the window sizes investigated (the smallest window is 500kb?) is biologically relevant. Some rough calculations given estimates of the age of the hybridization event, the generation time, and the recombination rate would give an idea of how small the windows would need to be to have a reasonable chance of representing one ancestry or the other.

My comment about the previous ln221 (ln 700 in the current response document) was specifically about what failure to diverge means in the context of that section. I.e., when comparing organellar histories. The idea here would be that somehow the e.g., plastid genome diverges (an accumulates substitutions) but the nuclear genome stays static? What is a biological mechanism for such a pattern? Similarly, in the next section of the response, "An example of such an event would be when a nuclear genome lineage ("host") diverges and the associated chloroplast genome lineage ("parasite" lineage)

is only associated with one of the two newly divergent nuclear genomes, having lost its association with the other."—I appreciate the biological example (this is what I'm looking for—a biological scenario that would be consistent with that model component, given the context that the model is being applied to). But I'm unclear about this example—what happens to the other nuclear genome lineage (it needs a chloroplast, too). What is a mechanism by which the nuclear genome can diverge, with one of the daughter lineages getting the ancestral plastid, and the other one getting something else?

I agree with this statement -- "Almost all current methods of detecting hybridization are based on testing the null modelthat ILS alone can explain conflicts among different parts of the genome (among loci, amongdifferent regions of the nuclear genome), or whether other biological processes (e.g., hybridization, horizontal gene transfer) must also be taken into account‟. However my concern, as I've tried to make clear, is about the risk of systematic errors, rather than biological factors.

"Secondly, Bruun-Lund et al. (2017) demonstrated that the unpartitioned chloroplast genomic dataset can recover more robust phylogenies in Ficus compared to a dataset partitioned in genes, and also confirmed that all main regions of the chloroplast genome share the same best substitution model (GTR+GAMMA), same model were also used in our case." First, getting higher support values is not an indication of better model fit (or of getting a more accurate answer). Second, this is an example of a potential source of systematic error. Just because an organelle is a single linkage group does not mean that the process of evolution is uniform across it. Similarly, a set of regions all being best fit by the most complex model type (GTR+G in this case), does not mean that those regions share specific parameter _values_. So they may all be GTR+G, but they could need different GTR+G matrices. Especially if the data include coding regions. I would bet my house that if you partition the data by codon position that model will have a better fit than does the unpartitioned version. It might not make a difference, but then again, maybe it will…

Question of relationships among Ficus, Morus, and Antiaris: I agree that discrepancies in outgroup relationships may not be relevant, however, outgroup composition can have a strong effect on relationships within the ingroup, especially when the position of root attachment is uncertain. So this issue should be examined, rather than ignored. And I've lost the thread of the details behind Fig. 1 (specifically, how was this time-calibrated?), but there's possibly a bigger problem here. I'm guessing that an ASTRAL tree was used for the topology, and if that's so, I suspect that the apparent error in the relationships among these three genera could be due to inappropriately rooting that ASTRAL tree (ASTRAL outputs an unrooted tree, and if one simply roots on the bipartition separating the outgroup and ingroup, one will get a monophyletic outgroup.) In that case, that's an error that needs to be corrected (the topology is wrong, because the root was designated in the wrong place). If other information (previous studies) show that Morus is sister to the rest of the included samples (Antiaris and Ficus), then the root needs to be placed on the bipartition separating Morus from the rest of the tree, prior to time-calibrating the tree.

The additional details about the geographic coverage of the samples is very helpful, and addresses my major concerns about the sampling. And I agree in general with -- "missing a few younger species or subclades with range-change events would not influence our reconstruction of ancestral distribution of Ficus, because those younger taxa just represent recent range extensions or range shifts."—but not fully, because those more recent events will elevate the inferred rates of range shifts, etc., so could change the inference elsewhere in the tree.

"Concerning your argument that the figure depicts inferred hybridization events between an ancestor and its descendant, which you consider impossible: Yes, such events would be biologically impossible if we only considered currently extant species or clades. However, the events depicted in the figure are NOT impossible if we take into account the (strong) likelihood that some lineages within these clades have gone extinct. Traces of ancient hybridization involving such "ghost" lineages can be preserved in the genomes of extant lineages." The issue of extinction and "ghost" lineages is

important in the context of this study. But does not provide an explanation for ancestor-descendant hybridization inferences. The hybridization event has to occur between two contemporaneous lineages. If one of those goes extinct or is not sampled (is a “ghost” lineage), that does not change the fact that they had to be contemporaneous in order to hybridize. The sequences from the “ghost” lineage not be disproportionally shared with one of the descendent lineages (lineages descended from the common ancestor of the ghost and the sampled taxa), so would not be picked up in ABBA BABBA. Something else has to be going on here.

Reviewer #3 (Remarks to the Author):

I thank the authors for addressing my previous comments and those of the other reviewers. I only have a few minor points.

Line 114: Italicize the "D" in D-statistics. This is done later in the manuscript and should be consistent. Also check line 576 and other instances I may have missed.

Line 150: Change "While" to "while"

Lines 159-161: Approximate estimates are given for mean ages, but precise estimates given for the credible intervals. Report the precise means with two decimal points for the results too.

Lines 188-189: This sentence seems incomplete. What are the implications? Do the authors mean to describe that introgression may be a matter of poor estimation, or that the results (e.g. introgression at the section level) is robust to species tree uncertainty.

Lines 238-240: This statement seems a bit wordy. It is also difficult to evaluate because these geographic ranges are so large it does not necessarily show sympatry.

Line 268: Remove italics from "any"

Line 308: missing "."

Line 395: "New World" is typically not treated as a compound word as in "New-World"

Line 539: Change "confidence" to "credible"

Line 545: This is probably a skew-normal distribution? This is hard to tell, because the tree (and alignment) is missing from the MCMCtree Appendix 3. Please include those. Table S4 is also not helpful in this regard as only minimum and maximums are given and it is not clear how this would be used to make a skew-normal calibration.

Fig 1: The red arrows seem a little clunky. Maybe an "*" would be less obtrusive.

###

Point-by-point reply to reviewers' comments

###

Reviewer #2 (Remarks to the Author):

The authors have done a herculean job in responding to reviews and revising the manuscript, and have addressed some of my concerns (thank you!). My main broad concern, however, is basically the premise of the paper—that incongruence is necessarily indicative of hybridization—and I don't think it can be addressed. The other reviewers seem to be satisfied, so I am happy leaving this issue to the discretion of the editors. Below I respond to the responses, and describe my remaining concerns. I should also note that I appreciate the framing of this paper as a "hypothesis generator" that invites further study. Additional emphasis on that framing would alleviate some of my concerns about the methodology.

Reply：Thank you for recognizing our extensive work in revising in response to the constructive criticisms. We agree completely that incongruence does not necessarily show hybridization. We say so in the manuscript, by pointing out that the incongruences we document just supplies additional support for the hypothesis of frequent hybridization inferred with other methods (lines 112-116). This is why we document so many different key features of these incongruences (for example, the evidence for wasp host shifts, the nuclear – cytoplasmic incongruences, and the geographic patterning of chloroplast lineages) that strongly suggest that hybridization is more likely than any plausible alternative. More importantly, the other kinds of data we report (results of the ABBA-BABA *D*-statistics, PhyloNetworks, and Bayesian Concordance) strongly support the hypothesis of hybridization.

The hypothesis of extensive hybridization in figs due to wasp host switches can now stimulate researchers to explicitly test the multiple hypotheses that we identify as being generated. We also added multiple words, such as "likely, possibly, inferred, suggested, putative", etc. throughout the main text (with highlighted color words), to tone down some of the assertions. We also further clarified the opportunities for testing this set of hypotheses in the revised version.

I think I may be being slightly unclear when I say "robust" phylogenetic analyses. This paper includes a very large molecular dataset, so I am not at all surprised that the inferences are strongly supported—that is not my issue. I am concerned, however, that systematic biases, which have the potential to become stronger with additional date, are not adequately investigated and accounted for. The ASTRAL results, for example, are not reliable, regardless of how strongly supported they are, if each input "gene" is not its own unlinked nonrecombining unit, and I don't think either criterion has been demonstrated in this paper (that each locus is free of recombination, and that the loci are not linked to each other). To reiterate—I do not think more

data are needed (at least, not more character data), but I do think greater attention could be paid to potential sources of systematic error.

Reply: Thank you for raising this important point. First, estimates of decay of linkage disequilibrium (LD) from several studies strongly suggest that conservatively LD decays by half after 10 kb and decays to background levels after 50 kb (unpublished work by Jie Gao, Gang Wang and Carlos Machado). For the coalescent-based species tree analyses (Astral) we used three window sizes: 50 kb, 100 kb and 500 kb. The distance between the 50-kb windows is 50 kb, while the 100- and 500-kb windows are non-overlapping but do not have gaps (see Supplementary Table 3). Based on estimated LD decay the 50-kb windows are effectively independent of each other, and the phylogenetic results from those windows do not differ dramatically from those from 100- or 500-kb windows, suggesting that LD does not seem to affect the analyses. Second, a recent Astral phylogeny (Rasplus et al. 2020 : https://www.biorxiv.org/content/10.1101/2020.04.15.042259v1.full) is strikingly similar in topology to the phylogeny we present, even though their data provide a much sparser genomic representation (ddRAD) and are thus more likely to be composed of unlinked loci.

In the revision, we emphasize that potential errors due to linkage or window sizes do not influence our main conclusion. First, as we noted previously in the reply accompanying our last revision, the most powerful evidence for the conclusion of frequent hybridization in this study comes from results of the ABBA-BABA analyses and *D*-statistics. There is broad agreement that this method is not affected by the phylogenetic topology (see methods section, lines 577-584), and its robustness has been widely tested (Green et al. 2010, Durand et al. 2011, Martin et al. 2013, Árnason and Halldórsdóttir 2019).

The conclusion of frequent pollinator host-switching is also robust, as it was supported by cophylogenetic analyses between all pairs of currently available fig and wasp phylogenies. These phylogenies are based on different methods (Astral and primary concordance trees, and even the tetrad analyses presented in our original manuscript) that use all currently available datasets (nuclear, chloroplast and mitochondrial genomes). No fig phylogeny is consistent with a strictly bifurcating branching pattern (i.e. species that do not hybridize).

Green, R. E. et al. A draft sequence of the neandertal genome. *Science* 328, 710-722, doi:10.1126/science.1188021 (2010).

Durand, E. Y., Patterson, N., Reich, D. & Slatkin, M. Testing for ancient admixture between closely related populations. *Mol. Biol. Evol.* 28, 2239-2252, doi:10.1093/molbev/msr048 (2011).

Martin, S. H. et al. Genome-wide evidence for speciation with gene flow in *Heliconius* butterflies. *Genome Res.* 23, 1817-1828, doi:10.1101/gr.159426.113 (2013).

Árnason, E. & Halldórsdóttir, K. Codweb: Whole-genome sequencing uncovers extensive reticulations fueling adaptation among Atlantic, Arctic, and Pacific gadids. *Sci. Adv.* 5, eaat8788, doi:10.1126/sciadv.aat8788 (2019).

The authors' point—"Most important, what we point out is that (i) neither of the two principal published phylogenies for fig wasps are congruent with any of the phylogenies (based on nuclear {Astral OR PCT tree}, chloroplast, or mitochondrial data {estimated using maximum likelihood approach}) for figs"—is well-taken. But that fact (the incongruence of all published fig phylogenies with all published wasp phylogenies) is not, in my opinion, sufficient evidence for the processes that this paper is claiming to establish. To give an extreme example, given that I know nothing about fig phylogeny, I could draw a fig phylogeny that would likewise be incongruent with the wasp phylogeny, and that wouldn't teach us anything about host switching or speciation. The absence of congruence is not sufficient evidence for these evolutionary processes.

Reply：We agree that absence of congruence between any two genetic datasets is not sufficient evidence to conclude hybridization, and exclude other possible explanations. However, there are multiple lines of evidence that reinforce each other and support the inference of hybridization. There is discordance of phylogenetic topologies based on nuclear versus cytoplasmic genes across the fig species. There is discordance even among components of the fig nuclear genomes. Finally, there is discordance between any supported phylogeny of the fig hosts and the phylogeny of the pollinators that serve as the primary vehicles for gene flow in figs.

The scenario described by the reviewer could be better invoked for a host-parasite system, where parasites switching hosts can lead to incongruent phylogenies with no need to invoke hybridization (or gene flow mediated by the parasites). More broadly, previous studies assume that co-speciation is the dominant process in the diversification of figs and wasps, and this assumption leads to the hypothesis that their phylogenies will be congruent. Our findings based on whole nuclear, chloroplast, and mitochondrial genome analyses (in addition to accumulating evidence from many other studies conducted by many other research groups) are inconsistent with this fundamental assumption. Because strict co-speciation appears to provide an inadequate description of the co-evolutionary process in figs and wasps, we suggest a series of more detailed geographical, genetic and experimental data to be collected and used to test these emerging hypotheses.

The additional details on the dual-indexing is helpful, but my question was about the extraction through to library prep. steps (and specifically the extraction). The comparison with new nanopore and PacBio datasets could be helpful in this regard, but that comparison is reduced to a single value ("inter-taxa genomic similarity"), which I can't imagine has the resolving power to detect small amounts of contamination, and won't help if the contaminating taxon isn't among the nine species compared (if the contaminant isn't one of the focal pair, than it would elevate the non-shared kmer count, rather than the shared kmer count, and would reduce the score). I note that the authors have still not addressed my original

question (this is my third time asking), about whether specific sequences were shared among samples (identical sequences, which would indicate very recent hybridization, or contamination).

Reply: The simple answer is that we found no signals of contamination, or of very recent hybridization. This result is expected, as the sampled fig species represent different subgenera and sections. A more complex reply is that all plant species share some sequences, as they all share a common ancestor. However, sequences shared from a common ancestor do not influence the results of hybridization-detection tests based on the three methods used here (the ABBA-BABA *D*-statistics, PhyloNetworks, and Bayesian Concordance), because they are considered in these analyses as an effect of ILS. Further, with respect to the comparison between the new Nanopore-PacBio datasets and the other datasets we used, any DNA contamination arising before and during library preparation will lead to a large increase in the measure of “inter-taxa genomic similarity”. However, we found no such signal among any of the nine tested species. Thus, we feel that DNA contamination is extremely unlikely.

Concerning the use of ML trees for both cytoplasmic-genome phylogenies, following reviewer 3’s comments. This point doesn’t affect the ultimate decision, but higher bootstrap values are not an appropriate justification for selecting an approach—just because the support is higher, doesn’t mean that the phylogeny is closer to the truth. Also, the issue with the quartet-based approach is not that there’s not enough SNPs but rather that the whole mitochondrial genome is one linkage group, and the method assumes unlinked loci. This is the sort of attention to the assumptions of the methods that I am concerned about with a paper of this scope.

Reply：Considering all the potential weaknesses of quartet-based approach methods that both you and reviewer 3 mentioned, we have discarded any mention of them and used the ML trees, beginning with the previous revision. However, we note that the central inference of hybridization remains unchanged.

“We suggest that these hybridization events are a dominant feature of fig/wasp coevolutionary history, and by generating novel genomic combinations in the figs have contributed to the remarkable diversity exhibited by this mutualism.” As before, I find this to be an interesting idea, but conjectural. Even if one accepts that there is something unusual about the species richness of Ficus (I am unconvinced), there is not evidence in this paper of a link between host-switching or hybridization and speciation. I suspect that if one were to poll evolutionary biologists, most would think that specialized pollinator fidelity would lead to higher speciation rates, and that hybridization would reduce diversification.

Reply：We are not asking for a poll of evolutionary biologists, we are asking that they consider the interpretations of the extensive data analyses we present, and further test them. We view your argument as in fact supporting the novelty of our study. The greatest value of our study in terms of its impact on future work may be precisely its challenging of a well-entrenched paradigm, by supplying

data that call the paradigm into question and that also suggest an alternative hypothesis that can now be subjected to further tests.

“We presented the tetrad tree (same method used as for the SVDquartets tree) in our first version. Generally, this method gives results consistent with those given by BUCKy, but we removed the results of tetrad trees owing to other potential weaknesses of this method (see Reviewer comments and our reply above, lines 249-284).” The concerns raised about the “tetrad tree” in the previous versions were about its application to organellar data, which are nonrecombining, and therefore a single linkage group. That concern does not apply to the nuclear genome, where these approaches would indeed be appropriate (and where the ASTRAL approaches, as implemented here, are not, for the reasons described previously).

Reply：Thanks for the clarification. It seems that we misunderstood your criticism in removing the tetrad tree results. We emphasize that we have used multiple methods that are consistent with the topology of the tetrad tree we first presented. None of the analyses undermine the fact that a history of hybridization in figs appears to present a better explanation than any plausible alternative for the observed patterns across multiple analyses of multiple fig and wasp datasets.

“Nevertheless, we obtained a consistent topology in the four Astral trees inferred based on windows of different sizes.” I appreciate this step, but am concerned that the window size still wasn’t small enough to control for recombination (and there’s a trade-off, because if the window is too small, there won’t be enough signal to infer a reliable gene tree in the first place). Note, also, that it’s not just a question of what the “main” bifurcating signal is. Because this paper is interested in hybridization (and applying BuCKy etc), the minority signals, such as one might get from a subset of loci including recombination events, is also important. The cited Shen et al. paper makes a related point, which is that a handful of “bad” genes can have a disproportionate influence on the results.

Reply：Please see our above reply to your second comment. Estimates of the decay of linkage disequilibrium in several fig species (unpublished studies by Jie Gao, Gang Wang and Carlos Machado) suggest that LD decays by roughly half after 10 kb and decays to background levels after 50 kb. The results from 50-kb windows do not seem to differ from those from 100- or 500-kb windows, suggesting that LD does not seem to affect the analyses.

ABBA BABA counts of hybridization events—so the number of significant introgression events for each four-taxon tree were added together to the total number of counts? It would be helpful to clarify more precisely how this was done, as an inference from a given four-taxon tree tests for introgression among those lineages, not just the tip species themselves. I.e., if there is a five-taxon tree -- ((A,B), C), D), E) -- and both the ACDE and BCDE four-taxon trees show an introgression, it could be because there are indeed two, or there could be just one, involving the ancestor of A and B. (I’m sorry, I’m having trouble explaining this clearly). This also relates to the comments about “ghost” lineages, later in the response to

reviews). (A ghost lineage that is/was sister to A+B could result in a positive ABBABABA between A or B and another extant lineage, but not between A or B and an ancestor).

Reply：Thanks for this comment and question. We further explained this point by adding the following (underlined text) in the manuscript (lines 598-601). “The doAbbababa2 program could calculate the *D*-statistics for all potential four-taxon combinations of species or groups. This means that for each species or clade one could potentially infer multiple hybridization events with other taxa if the species was included in multiple four-taxon groups. However, only those four-taxon groups that followed the phylogenetic topology of the Astral species tree were considered.”

“Based on this explanation, we can expect that short genomic windows have an advantage in detecting more ancient hybridization events, while long genomic windows are better at detecting more recent events.” -- I understand why the haplotype block size changes with the age of the hybridization event, but am not convinced that applying windows of a particular size blindly on the genome would capture that signal, and especially don’t understand why one would expect that large genomic windows would be better at detecting recent events. The smaller windows will have greater resolving power in all cases. (Provided that they have enough phylogenetic signal). I’m also not clear that the window sizes investigated (the smallest window is 500kb?) is biologically relevant. Some rough calculations given estimates of the age of the hybridization event, the generation time, and the recombination rate would give an idea of how small the windows would need to be to have a reasonable chance of representing one ancestry or the other.

Reply： Beginning with the last revised version, we only kept the PhyloNetworks results based on the 500-kb window dataset (for other analyses we also used 50-kb and 100-kb datasets). The PhyloNetworks results based on the larger windows (1000SNP windows dataset, mean window size ~1 Mb, range 438 Kb-4.16 Mb) were removed, as we were concerned about the potentially higher risk of recombination within larger genomic windows, which would influence the accuracy of hybridization detection. However, we agree that even the 500-kb window size is still very large, and may not be ideal for detecting older introgression events. However, just as you mentioned in your comments above, there is a trade-off for window size chosen. **This is precisely why we tested using different window sizes.** While short windows could conceivably be biologically more relevant, and permit more accurate gene tree inferences, or both, using them requires much greater computational resources. A window size of 500 kb was also used in the paper (Figueiro et al. 2017) recommended by Reviewer 3, which addressed the mentioned trade-offs. Fortunately, all hybridization events within *Ficus* clades detected with PhyloNetworks were corroborated with ABBA-BABA *D*-statistics, which do not suffer from the potential risk of recombination within genes.

For your questions about the effect of window size on hybridization detection with more ancient or relative recent events, the logic is similar to that in your understanding of how the most appropriate haplotype block size for hybridization detection depends on the age of the hybridization

event. There is a relationship between the time that has passed since hybridization and the size of chromosomal blocks in hybrids (Fisher 1954, Ungerer et al. 1998, Buerkle et al. 2008). After the initial hybridization event, the size of parental chromosomal blocks within the hybrids is expected to be continuously reduced over time, as a result of recombination during meiosis in heterozygous individuals. In other words, the introgressive genomic block due to a very ancient hybridization event is expected to be shorter than that due to a more recent event. Therefore, we expect that short genomic windows have an advantage in detecting more ancient hybridization events, while long genomic windows are better at detecting more recent events. Our results support this hypothesis: the PhyloNetworks analysis based on the 1000SNP windows dataset inferred only two hybridization events, whereas the PhyloNetworks analysis based on the 500-kb windows dataset inferred five hybridization events, with greater involvement of deep nodes (i.e., more ancient hybridization events) (Fig. 2, Supplementary Fig. 6).

Figueiro, H. V. et al. Genome-wide signatures of complex introgression and adaptive evolution in the big cats. Sci. Adv. 3, e1700299, doi:10.1126/sciadv.1700299 (2017).

Fisher RA. A fuller theory of "junctions" in inbreeding. *Heredity* 8, 187–197 (1954).

Baird, S. J. E. A simulation study of multilocus clines. *Evolution* 49, 1038-1045, doi:10.2307/2410429 (1995).

Ungerer, M. C., Baird, S. J. E., Pan, J. & Rieseberg, L. H. Rapid hybrid speciation in wild sunflowers. *Proceedings of the National Academy of Sciences* 95, 11757-11762, doi:10.1073/pnas.95.20.11757 (1998).

Buerkle, C. A. & Rieseberg, L. H. The rate of genome stabilization in homoploid hybrid species. *Evolution* 62, 266-275, doi:10.1111/j.1558-5646.2007.00267.x (2008).

My comment about the previous ln221 (ln 700 in the current response document) was specifically about what failure to diverge means in the context of that section. I.e., when comparing organellar histories. The idea here would be that somehow the e.g., plastid genome diverges (and accumulates substitutions) but the nuclear genome stays static? What is a biological mechanism for such a pattern? Similarly, in the next section of the response, "An example of such an event would be when a nuclear genome lineage ("host") diverges and the associated chloroplast genome lineage ("parasite" lineage) is only associated with one of the two newly divergent nuclear genomes, having lost its association with the other."—I appreciate the biological example (this is what I'm looking for—a biological scenario that would be consistent with that model component, given the context that the model is being applied to). But I'm unclear about this example—what happens to the other nuclear genome lineage (it needs a chloroplast, too). What is a mechanism by which the nuclear genome can diverge, with one of the daughter lineages getting the ancestral plastid, and the other one getting something else?

Reply： We agree that all viable fig nuclear genomes will have a corresponding chloroplast or mitochondrial genome. In some cases, hybridization will lead to novel nuclear-chloroplast associations.

I agree with this statement -- “Almost all current methods of detecting hybridization are based on testing the null model that ILS alone can explain conflicts among different parts of the genome (among loci, among different regions of the nuclear genome), or whether other biological processes (e.g., hybridization, horizontal gene transfer) must also be taken into account“. However my concern, as I’ve tried to make clear, is about the risk of systematic errors, rather than biological factors.

Reply： We agree completely that we should always be aware of the risk of systematic errors. As we have highlighted in our reply to your comments on the previous revision, we have gone to great lengths to avoid potential systematic errors (whole Methods part). We have done this by using strict data quality control (lines 467-510) and multiple analyses. Multiple commonly used procedures to detect hybridization supported the inference of frequent hybridization among *Ficus* plants. The agreement between different methods suggests that the potential risk of systematic errors should be very limited.

“Secondly, Bruun-Lund et al. (2017) demonstrated that the unpartitioned chloroplast genomic dataset can recover more robust phylogenies in Ficus compared to a dataset partitioned in genes, and also confirmed that all main regions of the chloroplast genome share the same best substitution model (GTR+GAMMA), same model were also used in our case.” First, getting higher support values is not an indication of better model fit (or of getting a more accurate answer). Second, this is an example of a potential source of systematic error. Just because an organelle is a single linkage group does not mean that the process of evolution is uniform across it. Similarly, a set of regions all being best fit by the most complex model type (GTR+G in this case), does not mean that those regions share specific parameter values. So they may all be GTR+G, but they could need different GTR+G matrices. Especially if the data include coding regions. I would bet my house that if you partition the data by codon position that model will have a better fit than does the unpartitioned version. It might not make a difference, but then again, maybe it will…

Reply：We agree completely with your comments. As you said, it may not make a large difference in this study, considering the relative stability of phylogenetic topology we found using different methods. Having established the potential influence of hybridization (and organelle exchange), this is exactly the kind of next step study and hypothesis testing that is needed.

Question of relationships among Ficus, Morus, and Antiaris: I agree that discrepancies in outgroup relationships may not be relevant, however, outgroup composition can have a strong effect on relationships within the ingroup, especially when the position of root attachment is uncertain. So this issue should be examined, rather than ignored. And I’ve lost the thread of the details behind Fig. 1 (specifically, how was this time-calibrated?), but there’s possibly a bigger problem here. I’m guessing that an ASTRAL tree was

used for the topology, and if that's so, I suspect that the apparent error in the relationships among these three genera could be due to inappropriately rooting that ASTRAL tree (ASTRAL outputs an unrooted tree, and if one simply roots on the bipartition separating the outgroup and ingroup, one will get a monophyletic outgroup.) In that case, that's an error that needs to be corrected (the topology is wrong, because the root was designated in the wrong place). If other information (previous studies) show that Morus is sister to the rest of the included samples (Antiaris and Ficus), then the root needs to be placed on the bipartition separating Morus from the rest of the tree, prior to time-calibrating the tree.

Reply：This is a fair point, but in all our analyses outgroup choice does not influence relationships within the ingroup. In fact, ingroup relationships were similar (and even the same basal clade was identified), regardless of whether one or two outgroups were used (Supplementary Fig. 1). To avoid potential confusion, we simplified the outgroup clade as an unresolved position in the revised Fig. 1 and Fig. 2.

The additional details about the geographic coverage of the samples is very helpful, and addresses my major concerns about the sampling. And I agree in general with -- "missing a few younger species or subclades with range-change events would not influence our reconstruction of ancestral distribution of Ficus, because those younger taxa just represent recent range extensions or range shifts."—but not fully, because those more recent events will elevate the inferred rates of range shifts, etc., so could change the inference elsewhere in the tree.

Reply：We thank you for your comments on the value of additional details about geographical sampling. Future studies will explicitly test precisely these more detailed questions.

"Concerning your argument that the figure depicts inferred hybridization events between an ancestor and its descendant, which you consider impossible: Yes, such events would be biologically impossible if we only considered currently extant species or clades. However, the events depicted in the figure are NOT impossible if we take into account the (strong) likelihood that some lineages within these clades have gone extinct. Traces of ancient hybridization involving such "ghost" lineages can be preserved in the genomes of extant lineages." The issue of extinction and "ghost" lineages is important in the context of this study. But does not provide an explanation for ancestor-descendant hybridization inferences. The hybridization event has to occur between two contemporaneous lineages. If one of those goes extinct or is not sampled (is a " ghost" lineage), that does not change the fact that they had to be contemporaneous in order to hybridize. The sequences from the "ghost" lineage not be disproportionally shared with one of the descendent lineages (lineages descended from the common ancestor of the ghost and the sampled taxa), so would not be picked up in ABBA BABBA. Something else has to be going on here.

Reply：Yes, we agree that hybridization has to happen between contemporaneous taxa, and that is the case here, as has also been demonstrated in previous studies (e.g., Durand et al. 2011, Yang et al. 2020). The confusion may due to the misunderstanding on the definition of the "ancestor", which is just the now-extinct clade, and close to the ancestor of extant clade, so it was represented by related ancestor in Figures. But this does not conflict with our explanation of hybridization between an extant taxon and a now-extinct clade, which should be picked up in ABBA-BABA type analyses, and cross-checked with other data and other analyses. To avoid potential confusion, we also clarified the definition of "ghost" hybridization in this revised version (lines 281-283) as: "Interestingly, several hybridization events suggest the possibility of ancient "ghost" hybridization events involving an extant taxon and a now-extinct clade[47] (Fig. 2, Supplementary Fig. 6-7)."

Durand, E. Y., Patterson, N., Reich, D. & Slatkin, M. Testing for ancient admixture between closely related populations. *Mol. Biol. Evol.* 28, 2239-2252, doi:10.1093/molbev/msr048 (2011).

Yang, Y. et al. Prickly waterlily and rigid hornwort genomes shed light on early angiosperm evolution. *Nature Plants*, doi:10.1038/s41477-020-0594-6 (2020).

##

Reviewer #3 (Remarks to the Author):

I thank the authors for addressing my previous comments and those of the other reviewers. I only have a few minor points.

Reply：Thank you very much for all your previous and current comments, which have been very useful to us in considerably improving our analyses and presentations in this manuscript.

Line 114: Italicize the "D" in D-statistics. This is done later in the manuscript and should be consistent. Also check line 576 and other instances I may have missed.

Reply：Done.

Line 150: Change "While" to "while"

Reply：Done.

Lines 159-161: Approximate estimates are given for mean ages, but precise estimates given for the credible intervals. Report the precise means with two decimal points for the results too.

Reply：Done.

Lines 188-189: This sentence seems incomplete. What are the implications? Do the authors mean to describe that introgression may be a matter of poor estimation, or that the results (e.g. introgression at the section level) is robust to species tree uncertainty.

Reply：Thanks. The sentence in question was reworded to read as follows (Lines 184-187): “Given its strong agreement with PhyloNetworks[45] analyses (see below) (Supplementary Fig. 6), we will primarily focus on the inferences based on the Astral tree topology for the remaining analyses. However, similar analyses were also conducted based on the alternative PCT tree topology and yielded similar results that suggest prevalent hybridization events.”

Lines 238-240: This statement seems a bit wordy. It is also difficult to evaluate because these geographic ranges are so large it does not necessarily show sympatry.

Reply：we reworded it as“The chloroplast genomes of sympatric (same geographical region), but apparently distantly related (clearly different subgenera) fig species often clustered in the chloroplast genome phylogeny.”

Line 268: Remove italics from "any"

Reply：Done.

Line 308: missing "."

Reply：Done.

Line 395: "New World" is typically not treated as a compound word as in "New-World"

Reply： “New-World” was replaced by “New World”.

Line 539: Change "confidence" to "credible"

Reply：Done.

Line 545: This is probably a skew-normal distribution? This is hard to tell, because the tree (and alignment) is missing from the MCMCtree Appendix 3. Please include those. Table S4 is also not helpful in this regard as only minimum and maximums are given and it is not clear how this would be used to make a skew-normal calibration.

Reply： Thanks. Both nuclear trees and aligned sequences used for MCMCtree analyses were added in the new Appendix 3. Only “log-normal distribution” was used in the MCMCtree program.

Fig 1: The red arrows seem a little clunky. Maybe an "*" would be less obtrusive.

Reply：Done. Thanks.
